# Impact of Microgravity on Cytoskeletal Dynamics, Protein Transport, and Signaling Networks: Potential Therapeutic Opportunities for Skin Health

**DOI:** 10.3390/ijms27177689

**Published:** 2026-08-27

**Authors:** Jamison M. Ballard, Rishitha Chiguru, Matthew B. Deeb, Rebecca B. Swatsburg, Ada Y. Lau, Grace H. Davis, Mevlana Demiri, Jewelia T. Keller, Beder Nourachi, Tasnim A. Abu Shihadeh, Rylin Schneider, Ericka Articulo, Naja M. Daye, Tasneem A. Awwad, Brianna Lamko, Samantha M. Bailey, Bella L. Guerra, Shayna L. Guerra, Corissa Quarterman, Meera Nanjundan

**Affiliations:** Department of Molecular Biosciences, University of South Florida, 4202 East Fowler Avenue, ISA2015, Tampa, FL 33620, USAjeweliak@usf.edu (J.T.K.); beder@usf.edu (B.N.); taabushihadeh@usf.edu (T.A.A.S.); awwad.tasneem@yahoo.com (T.A.A.); bailey123@usf.edu (S.M.B.); bellaloveg526@gmail.com (B.L.G.); shaynag26@gmail.com (S.L.G.);

**Keywords:** cytoskeleton, signaling, protein trafficking, skin, dermatology, space medicine, microgravity

## Abstract

Astronauts encounter several challenges on spaceflight missions, including space radiation and microgravity. Over the past several decades, studies conducted in the field of space medicine have uncovered broad impacts on human physiology, including the integumentary system, which serves as a protective barrier to environmental factors. Dermatological alterations in astronauts are commonly observed during spaceflight missions. Advancing our understanding of the intracellular mechanisms impacted in skin within the context of microgravity may uncover potential therapies for maintaining and improving skin health. Towards this goal, we utilized PubMed to survey current findings relevant to microgravity effects on cytoskeletal components, intracellular protein trafficking, and signaling cascades in mammalian model systems, in addition to identifying gaps in current knowledge. Concurrently across these studies, we focused on the specific cell culture technologies that were utilized during spaceflight missions and in microgravity simulated conditions. Altogether, we envision that these mechanistic insights will contribute to a better understanding of skin disorders such as atopic dermatitis and psoriasis, for which current treatment regimens are associated with adverse responses. Furthermore, the identified knowledge gaps will unveil new avenues for future spaceflight research to further propel scientific advancements in the field of space medicine.

## 1. Introduction

### 1.1. Space Exploration

Exploration of space is motivated by the human drive for discovery, towards the goal of uncovering benefits to support life on earth in addition to gaining new insights into aspects of the solar system and beyond. The “Space Race” started after World War II, and in 1961, Yuri Gargarin was the first human to venture beyond Earth [1,2]. This was followed by several missions that culminated in the first moon landing of Apollo 11 in 1969 [2,3]. Since the 1970s, there have been multiple manned spaceflights as well as the development of the International Space Station (ISS). The ISS represents a partnership spanning 15 countries and has now been in orbit for 25 years with over 280 visitations by astronauts (https://www.nasa.gov/international-space-station/, accessed 12 June 2026). The ISS provides the platform to network the diverse backgrounds of scientists towards common goals of space exploration. Space tourism is another objective of spaceflight, and these flights have been made with SpaceX, Virgin Galactic, Blue Origin, amongst others (https://www.nasa.gov/humans-in-space/leo-economy-frequently-asked-questions/#hds-sidebar-nav-4, accessed 12 June 2026). While the majority of astronauts have participated in spaceflights that have been in low-earth orbit, which is defined as a maximum altitude of 1200 miles (https://www.nasa.gov/humans-in-space/leo-economy-frequently-asked-questions/#hds-sidebar-nav-4, accessed 12 June 2026) and includes the ISS, engagement on cislunar travel flights have also been made [2]. Multiple cislunar missions are planned over the next decade, with the long-term objective of establishing a lunar base to support long periods of human habitation as well as propel future missions into deep space [4,5]. The lengths of implemented missions are largely variable, from minutes to months, with missions of far longer lengths (i.e., planetary missions to the moon and Mars) planned in the future [2].

### 1.2. Impact of Spaceflight on Health

Despite the immense societal and scientific advances from spaceflight, there are numerous reports of detrimental physiological effects on the human body. Although we direct the reader to detailed reviews of the physiological consequences, spaceflight effects include those on the musculoskeletal system [6], dermatological system [7], cardiovascular system [8], immune system [9], microorganismal behavior [10], kidney function [11], as well as changes in brain physiology [12], psychology, and cognition [13]. These effects are likely attributed to the combination of spaceflight elements such as microgravity, ionizing radiation, social isolation, confined living and working areas, distance from Earth, spaceflight time, light–dark cycles, noise, elevated carbon dioxide (CO_2_) levels, and long working hours [2,14,15,16,17].

### 1.3. Purpose of Article

**Figure 1:**
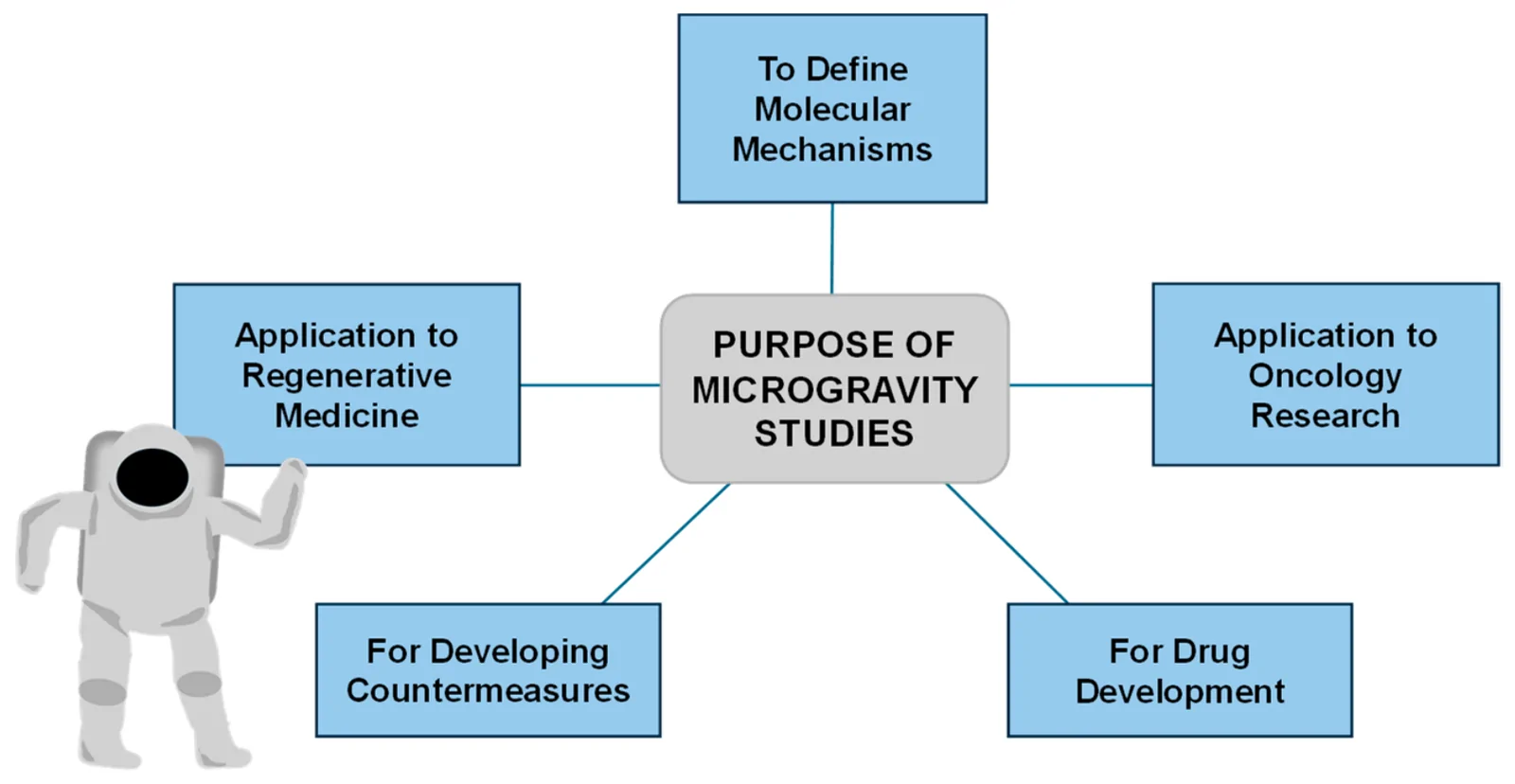
Purpose of microgravity studies and research applications. One goal of microgravity studies is to define the molecular mechanisms. Findings from these investigations may support drug development while also enabling the development of countermeasures to improve the health of astronauts venturing into space. Microgravity has been applied in various fields including cancer research and regenerative medicine.

Examination of the intracellular consequences of the above-described elements is ongoing and has yet to be fully defined. As shown in Figure 1, the knowledge acquired from these studies is essential to advance our understanding of the molecular mechanisms, develop countermeasures, and design effective medical treatments in the field of regenerative medicine and oncology. Earth gravity provides the constant and essential stressor necessary to sustain human bodily functions and those of other life forms [2]. However, when subjected to spaceflight, living beings will endure extreme changes in gravity, such as those encountered during the acceleration (i.e., launch), deceleration (i.e., landing), and weightless phases [2]. The key objective of this review is to provide the current foundation of knowledge on the effects of real and simulated microgravity, a major spaceflight hazard that has been well investigated [2], on major intracellular components (i.e., cytoskeleton, protein trafficking, signaling), with a focus on models relevant to skin health. Another objective is to identify gaps in knowledge and offer perspectives on future research directions. While this work sets forth the foundation for future research investigations, long-term real microgravity studies are necessary to validate these scientific findings [18]. In satisfying these objectives, an emphasis is placed on skin due to its critical role in mediating protection to environmental factors (i.e., spaceflight elements), thus bringing attention to developing improved treatment regimens to oppose its deterioration during spaceflight and improve quality of life for those who venture on these flights.

## 2. Applied Searches and Research Models

Generative artificial intelligence (GenAI) was not used in this paper to any extent (e.g., to generate text, data, or figures, or to assist in study design, data collection, analysis, or interpretation).

### 2.1. PubMed Search Methods and Analyses

To uncover the array of relevant research publications pertaining to the effects of microgravity on the cytoskeleton, protein trafficking, and major signaling cascades, various search terms presented in Supplementary File S1 (performed 14 June 2025), Supplementary File S2 (performed 14 June 2025), and Supplementary File S3 (performed 16 June 2025) were applied using PubMed. Search terms for cytoskeleton may have included “human”, “skin”, “microgravity”, or “spaceflight” along with “cytoskeleton”, “kinesin”, “dynein”, “keratin”, vimentin”, “neurofilament”, “actin”, “microtubules”, “tubulin”, “plectin”, “myosin”, and “lamin”. Search terms for protein trafficking may have included “human”, “skin”, “microgravity”, or “spaceflight” along with “protein trafficking”, “endoplasmic reticulum”, “golgi”, “endosome”, “lysosome”, “endocytosis”, “exocytosis”, “vesicle budding”, “vesicle”, “vesicle fusion”, “SNARE”, “Rab”, “tethering protein”, “glycosylation”, and “protein translocation”. Search terms for cell signaling may have included “human”, “skin”, “microgravity”, or “spaceflight” along with “cell signaling”, “MAPK”, “PI3K”, “AKT”, “JAK”, “STAT”, “GPCR”, “EGFR”, “PLC”, “PKC”, “adenylyl cyclase”, and “PKA”. Supplementary File S4 presents search terms (e.g., OMICS, Cell Culture Methods) that were applied to identify pertinent background material on microgravity methods as well as high throughout profiling technologies applied in the context of microgravity.

**Figure 2:**
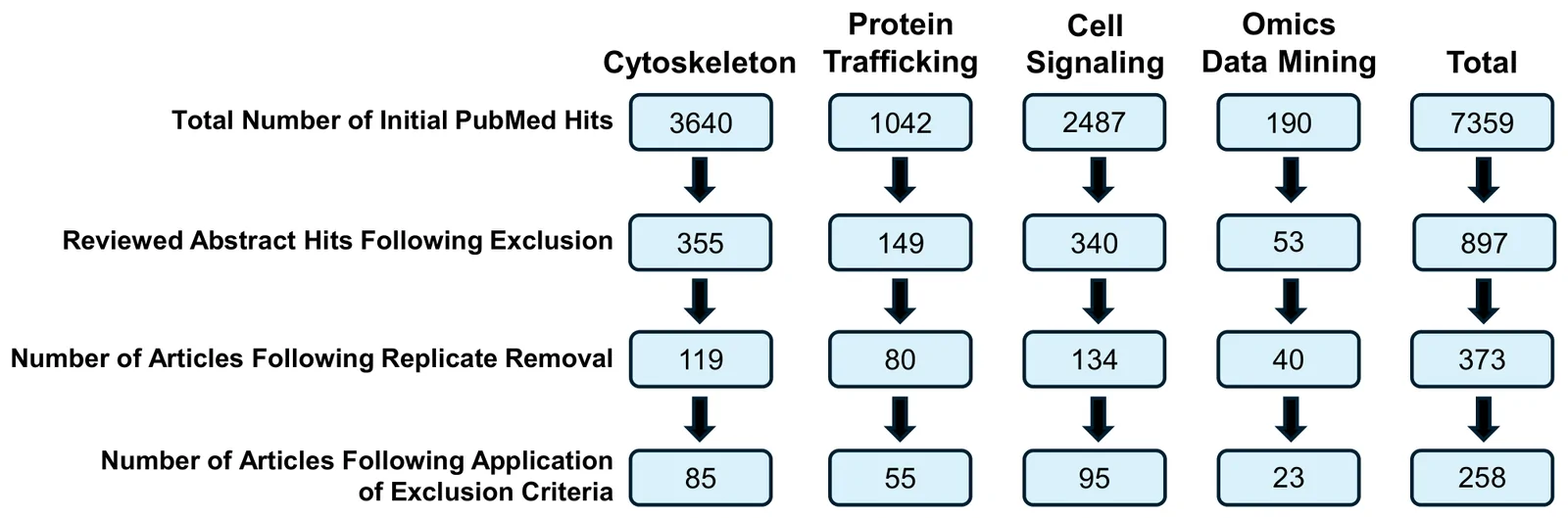
Overview of article selection methodology. The steps that were applied to identify the relevant articles for the four major areas reported in this review (i.e., cytoskeleton, protein trafficking, cell signaling, and OMICS data mining) are presented. The steps include the number of initial articles that were identified from PubMed using specific search terms, the number of abstracts for specific search terms that returned 50 or less hits from the cell signaling and protein trafficking segments were used, the “Myosin” search terms utilized in the cytoskeleton segment were eliminated due to their distant relationship to the skin, and, apart from this, the number of abstracts for specific search terms that returned 66 or less hits was used, the number of articles following removal of replicates, and the final number of articles following application of exclusion criteria.

Once the final relevant PMID list was generated within the above Supplementary Files, replicate PMIDs were removed from each and articles reviewed. Exclusion criteria included articles that were deemed irrelevant, not accessible (i.e., through the USF Library, Illiad, or in English), associated with quality concerns (i.e., data presentation, visualization, methodologies, duplicative sections across more than one article), or were associated with corrections or retractions (2 independent reviews were performed, specifically on 6 September 2025 and 15 November 2025). A schematic of the applied methodology and number of articles reviewed that were deemed relevant is depicted in Figure 2.

### 2.2. Microgravity Research Models

Microgravity has been generally associated with detrimental impacts on human health. However, it should be noted that there are reports of improved cellular outcomes under simulated microgravity environments such as increased viability, elevated proliferative capacity, and increased differentiation in specific cell types including stem cells or progenitor cells [19]. Thus, experimental application of microgravity is viewed as one with high value to advancing the biomedical field [20]. Specifically, the formation of 3D multicellular spheroids under microgravity conditions supports tissue engineering (i.e., joint repair, corneal tissue, adipose tissue [21]) and tissue regeneration [20,22]. The formation of 3D spheroids is also relevant to the field of cancer biology since the multicellular aggregates closely recapitulate the in vivo tumor, in contrast to a 2D culture, and could support efforts in drug discovery [22,23]. Finally, the application of microgravity is relevant to the stem cell engineering field [20].

Since spaceflight research is costly, most microgravity studies performed have been aboard short-term spaceflight missions or have utilized simulated microgravity equipment at ground-base [18]. Cell experiments on spaceflight missions are complex; a number of steps are necessary prior to initiating the experiment, including (a) preparing the cells at an appropriate density in a laboratory at ground-base, (b) transporting the cells to the launch site followed by their loading into a rocket prior to launch, (c) launching the cells aboard the rocket in an environment under different temperatures and CO_2_ conditions which may negatively impact cellular viability, (d) installing the experiment aboard the spacecraft, and (e) controlling for physical factors such as vibration and hypergravity phases [18].

Common models utilized across all three areas (i.e., cytoskeleton, protein trafficking, and cell signaling) include (a) immune and blood cells, (b) stem cells, (c) endothelial cells, (d) bone cells, (e) breast cells, (f) thyroid cells, (g) rodent models, and (h) human subjects. While only the cytoskeleton and cell signaling sections report skin-specific models, all segments utilize cell types that are relevant to skin composition and function. This includes immune cells, endothelial cells, mesenchymal cells, stem cells, amongst others (i.e., neurons, adipocytes, and fibroblasts) [24].

With respect to specific simulated and real microgravity methods applied along with their limitations, we direct the reader to a series of reviews that delve into the details of the engineering and physical aspects of these methods [21,23,25,26,27]. Table 1 (cytoskeleton, supported by Supplementary File S5), Table 2 (protein trafficking, supported by Supplementary File S6), and Table 3 (signaling, supported by Supplementary File S7) present a composite of the microgravity methods that were applied across all studies reviewed.

Details of the length of the microgravity application across these studies are included, which have uncovered a diverse range of times. For simulated microgravity studies, the range is across a time frame as short as 3 min to >6 weeks. Of note, the Random Positioning Machine (RPM) method was ranked as the most common simulated microgravity method applied. Other simulated microgravity methods that were applied include the Rotating Wall Vessel (RWV), Rotary Cell Culture System (RCCS), Clinostats (2D and 3D), Clinorotation, Clinochips, Gravite, and Magnetic Levitation. Further, a large array of rodent studies, specifically the hindlimb unloading/tail suspension model, were heavily applied throughout the cell signaling research segment. For real microgravity studies, the range is across a time frame as short as 22 s to >30 days.

As shown in Supplementary File S8 (cytoskeleton), Supplementary File S9 (protein trafficking), and Supplementary File S10 (signaling), the location of the launch site and author affiliations for the studies described herein are summarized. This includes studies aboard parabolic flights (majority organized by the French Novespace, on behalf of the German Aerospace Center), sounding rockets (ESRANGE space center in Sweden), the ISS, various spaceflight missions (majority launched from Kennedy Space Center in Florida, U.S.), and satellites (launched from various sites including Kazakhstan, Plesetsk base, and China National Space Science Center).

### 2.3. Separate Literature Search to Broaden Comprehensiveness of Review

Upon review of the selected articles and author affiliations, a pronounced imbalance was noted, favoring Western European and American research groups. However, it should be noted that there are other substantial bodies of research that have been conducted elsewhere (e.g., by Russian scientists), which were not identified through the search terms applied above. To broaden the comprehensiveness of the review, an independent series of searches was performed to include research performed by these other groups. These search terms are found in Supplementary File S11 (9 July 2026, 22 additional articles were analyzed from a total of 31), Supplementary File S12 (9 July 2026, 3 additional articles were analyzed from a total of 8), and Supplementary File S13 (9 July 2026, 10 additional articles were analyzed from a total of 19). The same exclusion criteria discussed earlier (e.g., English language accessibility issues) were applied. While these findings are not included in Figure 2 and Table 1, Table 2 and Table 3, these are discussed in Section 5.

## 3. Skin Health and Dermatoses

### 3.1. Skin Function and Health

Skin, the largest organ, elicits a broad array of roles. These include functions in (a) thermoregulation, (b) protection from trauma, (c) the endocrine system, (d) metabolism, such as production of vitamin D, (e) sense perception, (f) inflammation, (g) wound healing, and (h) microbial defense [28,29]. Due to its multifunctionality, the skin can impact organismal health at a systemic level. Parameters of skin health can be measured at the biophysical level [30]. These include assessment of epidermal hydration [31], pH [32], elasticity [33], and transepidermal water loss (TEWL) [34].

### 3.2. Skin Structure and Cellular Composition

**Figure 3:**
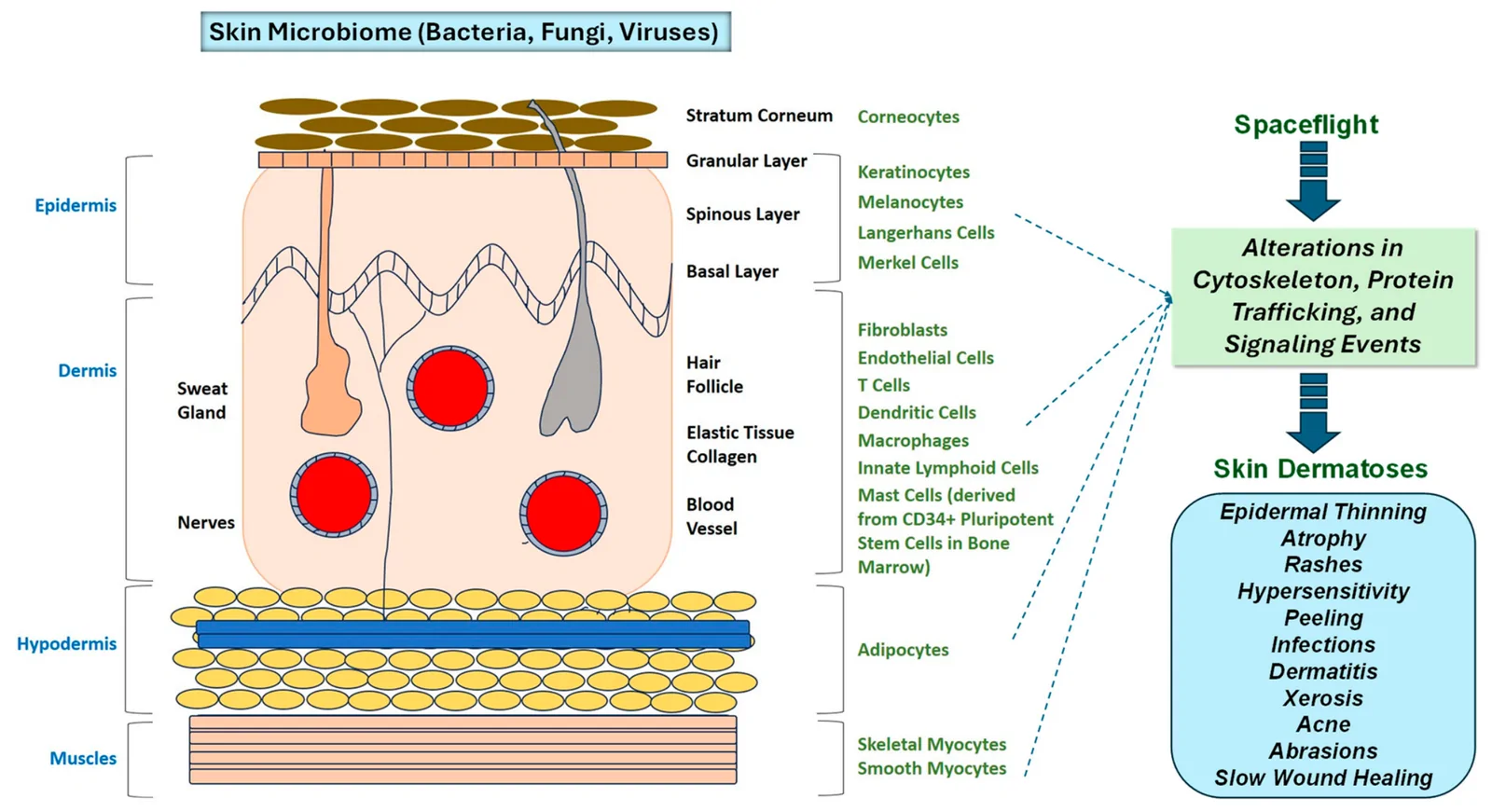
Skin structure, cellular composition, and dermatoses associated with spaceflight. The layers of the skin tissue are shown, along with the cell types found in each layer. Alterations in the cytoskeleton, protein trafficking, and signaling events across these cell types may contribute to the observed dermatoses noted during spaceflight.

As shown in Figure 3, the skin is organized into several layers, including epidermis, dermis, and hypodermis [28,35]. Beneath the hypodermis lies the deep fascia composed of muscles. The epidermal layer is primarily composed of keratinocyte cells, which undergo division in the basal layer [28,35]. These cells move outward through the spinous layer as they differentiate but no longer proliferate [28,35]. Keratinocytes reach the granular layer where they are terminally differentiated and eventually become corneocytes in the stratum corneum [28,35]. Other cell types present in the epidermis include melanocytes (melanin-generating dendritic cells), Langerhans cells (dendritic-like cells), and Merkel cells (keratinocyte-derived involved in touch responses) [28,35]. Within the dermal layer, there is a high abundance of extracellular matrix (e.g., collagen, elastin) generated by fibroblasts, which is necessary to maintain skin elasticity and stability [28,35]. In addition, there are blood vessels, thermoreceptors, mechanoreceptors, hair follicles, sweat glands, sebaceous glands, nerves, as well as immune cells (e.g., mast cells, macrophages, and lymphocytes) [28,35]. Adipocytes and connective tissue are found within the hypodermal subcutaneous layer [28,35].

### 3.3. Intracellular Pathways Regulating Skin Health

It is well established that inflammation of the skin can be induced through mechanical stimuli, infection, irritants, as well as ultraviolet radiation [35]. Inflammation of the skin can be accompanied by deregulated skin barrier function, immune cell infiltration into the dermal and epidermal layers, production of pro-inflammatory cytokines, alteration in keratinocyte functions, as well as changes in the composition of the extracellular matrix via altered fibroblast activities [35]. Diseases of the skin that are well established to be associated with inflammation include psoriasis, atopic dermatitis, hidradenitis suppurativa [36], amongst many others. The characteristics associated with psoriasis include severe inflammation, red skin plaques, breakdown of the skin barrier, along with a hyperproliferative epidermal layer [7]. Regrettably, psoriasis is associated with broader health impacts including metabolic syndrome, heart problems, depression, and arthritis [7]. Atopic dermatitis is another inflammatory skin condition in which the clinical features include severe itching, cutaneous infections, and dry scaly patches [37]. This disease is also characterized by abnormal skin barrier function [37].

With respect to the extracellular matrix (ECM) components in the dermal layer of the skin, the fibroblasts are key to its production [35]. The ECM can be regulated by the activities of matrix metalloproteinases (MMPs), which are secreted from several cell types (e.g., keratinocytes, macrophages, endothelial cells, as well as fibroblasts) [35,38]. Receptors that interact with the ECM include integrins, which function as mechanosensors [35,38]. Their activation can promote cells to survive as well as proliferate through activation of key signaling cascades [35]. In addition to integrins, mechanotransduction is regulated by cell–cell adhesion mediators such as those in adherens junctions, tight junctions, desmosomes, gap junctions, and the cytoskeleton [35,39]. Other regulators include β-catenin (involved in Wnt signaling, which is reduced in psoriasis [40]) as well as YAP1 (involved in Hippo signaling, with altered trafficking in psoriasis) [35,41].

Disruptions in the cytoskeleton deteriorate the skin barrier function and activate inflammation [35]. This process can be regulated by Rho GTPases, which modulate the activities of signaling pathways [35]. One cytoskeleton element, namely actin, is tightly connected to the integrity of cell–cell junctions and adhesion [35]. While intermediate filaments are well established to support the mechanical strength of cells, they are linked to actin filaments, integrin, and the ECM [35].

Physiological levels of endoplasmic reticulum (ER) stress are necessary to maintain skin health [42,43]. For example, mediators of ER stress (i.e., XBP1, CHOP, BiP) are elevated during differentiation of keratinocytes [42,43]. However, when ER stress becomes deregulated due to stressors, the unfolded protein response pathway (UPR) is activated, leading to cell death, as noted in various skin diseases [43].

Signaling cascades are essential to regulate gene expression of multiple targets, which modulate functional outcomes in the skin [29]. For example, the JAK–STAT pathway regulates the production of over 50 cytokines, including interleukins and interferons in multiple cell types (i.e., keratinocytes, dendritic cells, mast cells, T-cells, fibroblasts) [29]. The NFkB pathway also regulates cytokine gene expression as well as those involved in adhesion via the Toll-like receptors (TLRs) [29]. MAPK is a central pathway that has been shown to regulate the integrity of the skin barrier as well as contribute to wound healing, keratinocyte differentiation, and cytokine gene expression [29]. PI3K/AKT contributes to cell survival and proliferation in response to cytokine stimulation and can also promote factors to be secreted [29].

**Figure 4:**
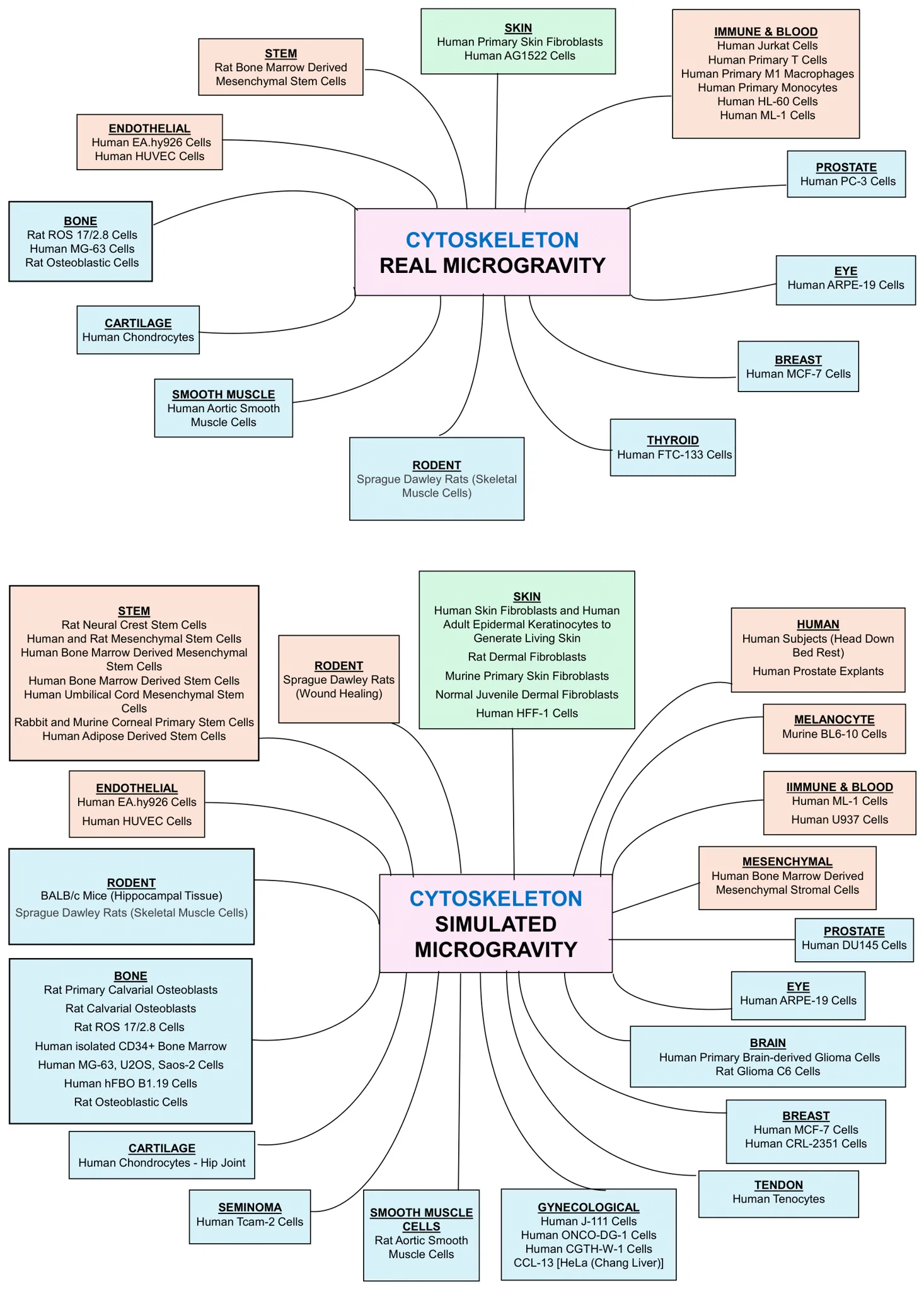
Real and simulated microgravity studies pertaining to the cytoskeleton. Model systems are organized according to real microgravity and simulated microgravity studies in the top and bottom panels, respectively. Specifically, model systems pertaining to the skin are displayed in green. Model systems displayed in orange are cell line models that are relevant to skin tissue, while those displayed in blue are not relevant to skin tissue.

The factors that induce inflammation are akin to the types of exposures encountered by astronauts on spaceflights; these include changes in microbe distribution, gravity, and pressure, as well as exposure to cosmic radiation and cosmic dust [7]. As detailed in Figure 4, skin dermatoses observed during spaceflight include epidermal thinning, skin atrophy, rashes, hypersensitivity, peeling, infections, dermatitis, xerosis, acne, abrasions, and diminished rate of wound healing [7]. The following section serves to lay the foundation of knowledge for changes that occur in various intracellular pathways (i.e., cytoskeleton, protein trafficking, and signaling events) impacted by microgravity in both skin and non-skin models. Furthermore, connections to alterations observed in skin disorders are included.

## 4. Effects of Microgravity on Cellular Outcomes

Cellular outcomes can be regulated by external stimuli including chemical and mechanical cues [44]. Specifically, mechanical stimuli may include alterations in the stiffness of the extracellular matrix (ECM), shear, pressure, and other forces [45,46]. Cells sense such mechanical cues through cell surface receptors, ion channels, integrins, and focal adhesions, which are then conveyed to the cytoskeletal network and signaling cascades; this process is referred to as mechanotransduction [45]. Further, the nucleoskeleton is well established to respond to such changes in the cytosolic compartment via LINC (linker of nucleoskeleton and cytoskeleton complex [47]), which can then alter gene expression patterns [45]. It is expected that changes in the cytoskeleton (i.e., intermediate filaments, actin filaments, and microtubules) and associated elements (e.g., motor proteins such as dyneins and kinesins) will impact the cell at multiple levels, including its tensile strength, protein trafficking events through vesicular transport, organellar structure and movement, as well as cell morphology, cell division, and cell motility. Furthermore, the endoplasmic reticulum (ER), the unfolded protein response (UPR), and autophagy contribute to mechanosensing [48,49]. Signaling pathways including Hippo and Wnt are known to be involved in the mechanotransduction process. Key mechanosensors in these cascades include YAP, β-catenin, and NFκB [47]. Other notable pathways with roles in mechanotransduction include MAPK, JAK–STAT, PKC [44,50], and PI3K/AKT [51].

While these events are part of the normal cellular process, abnormal mechanical cues (e.g., in time, in direction, and in strength) including those generated under microgravity conditions can lead to the pathogenesis of various diseases, including cardiovascular fibrosis, pulmonary fibrosis, renal fibrosis, amongst others [45]. As discussed below in the array of reviewed studies, a range of microgravity methodologies along with variable experimental findings were uncovered. Furthermore, only one aspect or a subset of the above components appears to have been investigated across many of these studies. Therefore, a more comprehensive analysis of multiple components of the mechanotransduction network could be assessed in the future to more broadly advance our understanding of how microgravity impacts the connection between the cytoskeleton, trafficking events, and signaling networks.

### 4.1. Cytoskeleton

A subset of studies that were uncovered through our initial PubMed searches investigated the impact of microgravity on cytoskeletal outcomes using skin-relevant models. Cytoskeletal elements that were investigated in response to microgravity (real and simulated) included actin filaments, intermediate filaments, and microtubules as well as associated cytoskeletal elements. Details of these studies are summarized in Supplementary File S14, and a schematic of the experimental models used is presented in Figure 4, along with the relevant citations presented in Supplementary Table S1.

#### 4.1.1. Integumentary System

As discussed above in Section 3, there are a number of cell types that exist in the skin tissue and contribute important functions to maintenance of skin health. The studies discussed below describe the effects of microgravity on various skin-derived fibroblast cells as well as melanocytes and rodent models.

*Mixed—Simulated Microgravity using Cells:* A series of skin-derived fibroblasts were utilized to investigate the effect of simulated microgravity on the cytoskeleton. For example, in rat dermal fibroblasts, RPM caused strengthening of F-actin bundling (at 30 min), which was then followed by thinning out along with increased RhoA GTPase protein (a cytoskeleton-associated element, via Western analysis) and reduced RNA levels of β3-integrin and collagen (via qPCR) at 48 h [52]. When RPM (for 14 days) was applied to murine primary skin fibroblasts, Affymetrix arrays uncovered reduced RNA transcript levels of ACTG2 (actin gamma 2) and ACTA1 (actin alpha 1) [53].

With human-derived cells, specifically normal human juvenile dermal fibroblasts, 3-day application of RPM led to the appearance of both adherent (AD) and multicellular spheroid (MCS) cultures [54]. While there was no change in the RNA or protein levels of tubulin beta (TUBB) and actin, vimentin (an intermediate filament) protein increased in AD cultures [54]. With respect to cytoskeletal-associated elements, vinculin protein diminished in AD and MCS cultures, as determined by immunofluorescence staining [54]. Under simulated microgravity conditions with the NASA bioreactor (RCCS, for 1 to 5 days), living skin equivalents (developed using human foreskin fibroblasts and human normal epidermal keratinocytes from adults) displayed no changes in the intermediate filament, cytokeratin [55]. With the 2D clinostat, human foreskin fibroblasts (HFF-1) elicited reduced migration (24–36 h), which coincided with depolymerization of F-actin filaments [56]. Lysophosphatidic acid (LPA, a potent mitogen) was found to oppose these changes by recovering the cellular migratory capacity [56]. Furthermore, jasplakinolide (JASP), which induces polymerization and stability of actin, recovered the F-actin network and cellular migration [56].

Altogether, various simulated microgravity conditions (RPM, 2D clinostat, RCCS) were applied for different lengths of time (30 min, 14 days, 1–5 days, 24–36 h) across skin fibroblasts derived from different model systems. Furthermore, the cytoskeletal elements assessed and methodologies applied (e.g., qPCR, Western analyses, immunofluorescence) varied across the studies. Since protein-level changes in the cytoskeleton were noted (in content and organization), these alterations are expected to impact skin functions, including the integrity of the skin barrier. LPA and JASP were also demonstrated to oppose the cellular changes, which could be further studied as therapeutic interventions for various skin dermatoses encountered during spaceflight.

Other skin-relevant cell types that were investigated include melanocytes, which exist in the epidermal layer of the skin and are responsible for producing the pigment, melanin. Studies have reported cytoskeletal alterations in these cells in response to simulated microgravity. Murine BL6-10 melanoma cells subjected to RPM for 24 h displayed disrupted microtubule networks as well as loss of actin filaments at the cell surface [57]. These alterations were associated with reduced p-p65 NFκB protein and its diminished nuclear translocation [57]. In an independent study, BL6-10 melanoma cells subjected to RPM for up to 3 days displayed reduced F-actin stress fibers and lamellipodia (at 1 day) with reduced lamin A protein expression and localization at the nuclear membrane (at 2 days) [58]. Along with these changes, focal adhesions were reduced (i.e., paxillin and vinculin at 1 day) along with reduced Rho GTPases, specifically RhoA, Rac1, and Cdc42 [58]. In addition, reduced p-NFκB protein and its cytoplasmic retention were reported [58]. Further, reduced p-ERK1/2 and p-FAK (Y937) levels were noted [58]. These alterations could be reversed by treatment with CNF1 toxin from *E. coli*, which could also elicit a positive outcome on cellular survival [58]. Since the cytoskeleton has been linked to melanosome transport [59], conditions of microgravity may alter skin pigmentation; this aspect was lacking in the above studies, but further research in this area may be worthwhile.

*Simulated Microgravity using Rodent Models:* Using wounded Sprague Dawley rats that were subjected to hindlimb suspension, reduced healing was associated with reduced fibroblasts in the wounded region during the early phase (day 4 to 6), with recovery by day 8 [56]. LPA treatment was able to improve both the healing and migration outcomes in this model system [56]. The simulated microgravity study using HFF-1 cells by this same research group supports this in vivo data and offers some mechanistic insight into the altered wound-healing response [56]. The use of LPA (and the receptors to which it binds) could be studied further as a therapeutic intervention in skin dermatoses associated with reduced wound-healing responses [60].

*Mixed—Real Microgravity:* In a MASER 5 sounding rocket mission wherein the real microgravity phase lasted ~6 min, no visible changes on the F-actin organization (via phalloidin staining) were noted using human primary skin fibroblasts [61]. Aboard the ISS, 14 days of real microgravity did not induce changes in alpha-tubulin or fibronectin cell staining using human AG1522 foreskin cells [62]. Across these two studies with different time frames of microgravity exposure, no changes were uncovered in the cytoskeletal elements studied at the protein level. Further work could be conducted to examine a more comprehensive array of cytoskeletal molecules at both the RNA and protein level.

#### 4.1.2. Stem Cells

Mesenchymal stem cells are multipotent and have the potential to differentiate into skin-relevant cell types under specific conditions, with potential in skin healing [63]. Other types of stem cells that were assessed for potential cytoskeleton changes included corneal primary stem cells and adipose-derived stem cells.

*Actin—Simulated Microgravity using Cells:* Application of human mesenchymal stem cells to the RCCS (HARV) simulated microgravity device for 7 days led to disruption of the actin network, which was characterized by the lack of stress fibers along with reduced levels of activated RhoA GTPase and p-cofilin protein levels [64]. However, actin stress fibers as well as p-cofilin and p-FAK levels were recovered upon expression of RhoA-V14, in which the GTPase activity is constitutively activated [64]. In an independent study, these stem cells were applied to Clinochips (for up to 8 h), which caused reduced cell spreading and migration in response to NGF [65]. Further, using rat mesenchymal stem cells subjected to 2D clinostat for 24 h, reduced migration and increased thickening of the F-actin network along with cellular stiffness were noted [66]. These alterations could be hindered using Y-27632, a Rho-associated kinase (ROCK) inhibitor [66]. Across these studies using mesenchymal stem cells, the cytoskeleton is disrupted (at the protein and organization level) under microgravity, leading to the functional outcome of reduced migration.

*Intermediate Filaments—Simulated Microgravity using Cells:* A subset of studies using other types of stem cells investigated changes in intermediate filaments, notably vimentin. Rabbit and murine corneal primary stem cells, isolated from corneal stromal tissues, were subjected to simulated microgravity (RCCS) for up to 7 days [67]. The cells formed reduced-sized spheroid structures that were devoid of vimentin, compared to controls, as noted via cell staining [67]. With human adipose-derived stem cells subjected to 2D clinostat for up to 7 days, vimentin RNA expression was unchanged [68]; however, in an independent study, vimentin RNA levels were reduced when the cells were subjected to the clinostat for 7 days [69]. When these cells were subjected to simulated microgravity using RWV for 14 days, no change in vimentin staining was noted relative to cyclic hydrostatic pressure (CHP, as a form of cyclic loading) in which it was elevated [70]. Across these studies using either corneal stem cells or adipose-derived stem cells, the outcomes were varied, ranging from decreased to no change in vimentin. This varied depending on the type (and time) of simulated microgravity exposure as well as experimental method to assess changes in the cytoskeleton (e.g., RNA or protein levels or localization).

*Mixed—Simulated Microgravity using Cells:* Rat neural crest stem cells subjected to up to 48 h of simulated microgravity using the RCCS device displayed disorganized and shortened F-actin filaments along with reduced vimentin protein levels [71]. In addition, CXCR4 (a chemokine receptor) and RhoA proteins were increased at the cell surface and cytoplasmic compartments; further, ROCK1 protein was elevated at the cell surface along with increased p-p38 (T180/Y182) protein levels [71]. However, these changes could be reversed using pertussis toxin (PTX) or small inhibitory RNA targeting CXCR4 [71]. These also demonstrate that the cytoskeleton is disrupted and adds other components (e.g., signaling) that may be contributing to these outcomes, which are upstream. Specifically, the involvement of a CXCR4/CXCL12 axis has been reported in melanoma, in which it participates in regulating motility and proliferative capacity [72]. Thus, targeting this pathway may offer a therapeutic opportunity.

Using the Gravite simulated microgravity device for up to 3 days, human mesenchymal stem cells displayed an array of cytoskeletal alterations including enlarged cortical actin fibers in filopodia and lamellipodia, elevated paxillin enriched at leading edges, elevated activated p-paxillin protein, and enriched lamin B at the nuclear periphery with decreased lamin A/C at the apical side [73]. This microgravity device was also applied to human bone marrow-derived stem cells for up to 15 days, which also caused enlarged actin fibers in the filopodia and lamellipodia regions with reduced lamin A/C levels [74]. In human bone marrow-derived mesenchymal stromal cells subjected to RPM, actin (24 h, with recovery at 120 h) as well as vinculin (6 to 120 h) were both reduced in intensity and redistributed to the cell center from the plasma membrane region [75]. In contrast, there were no changes in the organization of the microtubule network under these conditions [75]. Human umbilical cord mesenchymal stem cells subjected to clinostat for up to 72 h resulted in reduced beta-actin and alpha-tubulin expression at both the RNA and protein levels; these changes were associated with an absence of F-actin bundling and microtubule networks [76]. Across these studies using mesenchymal stem cells, there are changes in actin networks, nuclear intermediate filaments, focal adhesions, as well as microtubules.

*Mixed—Real Microgravity using Cells:* Using rat bone marrow-derived mesenchymal stem cells, real microgravity applied via the SJ-10 satellite for 2 and 10 days resulted in reduced levels of beta-actin, increased levels of bundled alpha-tubulin, and altered localization of vimentin to perinuclear regions [77]. In addition, the associated cytoskeletal element Rho GTPase, RhoA, was increased while Rac1, Cdc42, and ROCK were reduced at day 10 [77]. This real microgravity study demonstrates that the cytoskeleton is disrupted and also adds the Rho/ROCK signaling cascade, which may contribute as a regulator of these outcomes. The skin epidermal layer is well established for its renewing properties; further, the Rho/ROCK network is essential to this process [78]. Thus, deregulated Rho/ROCK signaling is expected to disrupt the functional integrity of the skin tissue.

#### 4.1.3. Immune System

It is well established that the immune system is suppressed in spaceflight [9]. Furthermore, the skin, which plays an important role in antimicrobial defense, undergoes a microbial shift in spaceflight [79]. As indicated below, a range of immune cells were assessed for changes in cytoskeletal elements, including CD34+ bone marrow cells, U937 pro-monocytic cells, monocytes, Jurkat T cells, and HL-60 leukemia cells.

*Actin—Simulated Microgravity using Cells:* A subset of studies using immune cells were examined for changes in actin. Simulated microgravity with the RWV device for 1–3 days caused isolated human CD34+ bone marrow cells to fail to undergo erythroid differentiation [80]. This coincided with reduced F-actin expression and diminished cellular migration [80]. While the PKC activator, PMA, did not markedly alter the migratory capacity, it increased the cellular proliferation [80]. In another study, human U937 pro-monocytic cells subjected to RWV elicited reduced proliferative capacity at 48–192 h, which was associated with reduced actin protein levels at 72 h [81]. The outcomes of these studies may be relevant to immune cells present within the skin dermal layer; such immune cells may undergo similar cytoskeletal alterations in response to simulated microgravity, leading to various skin dermatoses.

*Actin—Real Microgravity using Cells:* In a real microgravity study performed on the TEXUS-54 sounding rocket mission (~6 min microgravity) with human primary monocytes, no change in actin protein was noted via use of the FLUMIAS live cell microscopy system [82]. These findings contrast the simulated microgravity outcomes for actin but may be due to the comparatively short exposure to microgravity.

*Intermediate Filaments—Real Microgravity using Cells:* Human Jurkat T cells, subjected to real microgravity (~12 min) aboard the MAXUS-2 sounding rocket mission, displayed increased vimentin bundling along with disorganized networks [83]. It is unclear how these changes relate to changes in other cytoskeletal elements under these experimental conditions.

*Microtubules—Real Microgravity using Cells:* To assess changes in microtubule organization, real microgravity studies aboard the STS-67 shuttle mission were performed using human promyelocytic HL-60 leukemia cells for 3 days [84]. Cell staining for tubulin identified reduced microtubule branches [84]. In another real microgravity study aboard the Space Shuttle using human immortalized Jurkat T lymphocytic cells, the microtubule organizing center (MTOC) was not well defined and was associated with shortened filaments at 4 to 48 h, as noted via tubulin staining [85]. Altogether, the microtubule network was disrupted under these real microgravity conditions and is expected to impact vesicular movement, cell division, amongst other intracellular functions.

*Associated Cytoskeletal Elements—Real Microgravity using Cells:* Human immortalized Jurkat T lymphocytic cells subjected to real microgravity aboard the STS-95 mission for 24 and 48 h showed an array of cytoskeletal alterations including plectin (a cytoskeleton crosslinker) and MYL5 (a myosin motor protein component), as determined via cDNA microarray [86]. Plectin is a linker cytoskeletal protein, and its disruption is expected to elicit broad effects. Alterations in plectin are associated with skin dermatoses including epidermolysis bullosa simplex [87].

*Mixed—Real Microgravity using Cells:* Activated human primary T cells were subjected to real microgravity aboard the MASER-12 suborbital mission (~6 min of microgravity) [88]. In this study, there was no significant change in the vimentin and beta-tubulin proteins compared to in-flight 1 g controls [88]. In another real microgravity study aboard the ISS, human primary M1 macrophages displayed no change in actin or vimentin staining patterns at 11 days; however, both fibers and filaments were reduced at 30 days [89]. From these two independent studies using macrophages or primary T cells, the short time exposure (few minutes up to 11 days) to real microgravity appears to result in no change in the cytoskeletal elements analyzed, whereas the longer exposure time (30 days) resulted in alterations.

#### 4.1.4. Cardiovascular System

It is well known that spaceflight causes atrophy of myocardial tissue and alterations in the endothelium [90]. To understand the impact of microgravity exposure to the microvasculature of the skin which harbors blood vessels, the cytoskeletal changes acquired in endothelial cells were reviewed.

*Actin—Simulated Microgravity using Cells:* In human EA.hy926 hybrid cells (human umbilical vein cells fused to A549 lung adenocarcinoma cells) subjected to clinostat for 2 h, elevated cellular motility was observed; furthermore, filopodia and lamellipodia-like elements (which contain actin filaments) were observed via cell staining [91]. These changes were, however, opposed by cytochalasin D, an inhibitor of actin polymerization [91]. The increased endothelial cell migration noted under these simulated microgravity conditions contrasts to the reduced migratory capacity of skin fibroblasts and stem cells. These varied effects of microgravity may be cell type-dependent.

*Actin—Real Microgravity using Cells:* In contrast to the simulated microgravity outcomes, subjecting EA.hy926 cells to real microgravity through parabolic flight campaigns aboard the Airbus A300 ZERO-G (~22 s of microgravity) did not alter beta actin (*ACTB*) RNA expression [92]. The relatively short microgravity exposure period may explain the lack of change in beta actin RNA, suggesting future work under real, prolonged microgravity conditions to study the actin changes at the protein level in endothelial cells.

*Microtubules—Simulated Microgravity using Cells:* Human umbilical vein endothelial (HUVEC) cells subjected to simulated microgravity using the RPM device for 24 h displayed increased tubulin protein expression (e.g., TUBA1A, TUB4B, TUBB1, TUBB2A, and TUBB8), as determined by mass spectrometry of the secretome [93]. In addition, cytoskeletal-associated elements in the secretome (e.g., KAT-NAL2, DNAH11, DYNCLl1) were increased; these molecules are involved in regulating the activity of microtubules [93].

*Microtubules—Real Microgravity using Cells:* HUVEC cells were also assessed aboard the ISS in a real microgravity study [94]. After-mission HUVEC cells elicited a flattened phenotype with blebbing, while readapted HUVEC cells were enlarged with disrupted microtubules [94]. Across both simulated and real microgravity, microtubule disruption is evident and may thus contribute to skin tissue healing and regeneration [95].

*Mixed—Simulated Microgravity using Cells:* Using HUVEC cells subjected to 24 h of simulated microgravity with the RPM device, reduced levels of actin and tubulin proteins along with disorganized networks of the MTOC were noted [96]. Using human endothelial EA.hy926 cells subjected to simulated microgravity using RPM, increased cytokeratin proteins with redistribution to the perinuclear region and microtubular perinuclear localization were observed at 24 h [97]. While elevated levels of vascular endothelial growth factor receptor (VEGFR) protein (at 4 to 12 h) as well as extracellular matrix (ECM) components such as collagen I, laminin, chondroitin sulfate, and fibronectin at 4 h were noted, VEGF treatment diminished these ECM components [97]. In an independent study using the same cell line subjected to RPM, perinuclear localization of actin and microtubules was observed at 10 days following an earlier reduction in F-actin between 30 min and 2 h [98]. In these endothelial cells subjected to RPM for 28 days, formation of branched structures that stained positively for F-actin, α-tubulin, collagen I, and collagen III were noted [99]. Across these studies with different microgravity exposure times, the cytoskeleton is altered consistently and is associated with changes in the ECM components.

*Mixed—Real Microgravity using Cells:* Under real microgravity conditions applied via parabolic flight campaigns (~22 s of microgravity), EA.hy929 cells showed changes in beta-tubulin organization with its redistribution to the perinuclear region [100]. Aboard the SJ-10 satellite for 10 days as a real microgravity study, these cells displayed reduced beta-actin and alpha-tubulin protein levels that were associated with a depolymerization phenotype [101]. Other changes included elevated vimentin, RhoA, and ROCK proteins at 10 days with unchanged Rac-1 levels and reduced Cdc42 proteins [101]. Similar to stem cells, changes in the Rho/ROCK signaling pathway are noted in endothelial cells with exposure to real microgravity conditions.

#### 4.1.5. Muscular System

Muscular atrophy is a well-established outcome of spaceflight [6]. With respect to skin, the dermal sheath in the dermal layer contains smooth muscle that lines the hair follicle [102]. To uncover the effect of microgravity on the cytoskeletal network in this context, studies using human smooth muscle cells were assessed.

*Actin—Real Microgravity using Cells:* In a study aboard the ISS real microgravity environment for 3 days, the actin cytoskeleton network in human aortic smooth muscle cells was examined. Specifically, RNA sequencing of these muscle cells uncovered diminished contractile vascular function due to the reduction in RNA expression of alpha actin 2 (ACTA2), talin 2 (TLN2), and filamin A (FLNA) as well as elevated RNA levels of alpha actin 3 (ACTN3) and calponin 1 (CNN1) [103]. It is unclear whether these RNA-level changes are associated with protein-level changes across these cytoskeletal mediators or even changes in cytoskeletal organization. Further work is needed to examine actin alterations in smooth muscle cells derived from dermal skin upon microgravity exposure.

*Intermediate Filaments—Simulated Microgravity using Cells:* With human tenocytes subjected to a 9-day simulated microgravity period using RPM, RNA levels of vimentin were unchanged in both 2D and 3D cultures [104]. Tenocytes are found in tendons with a role in ECM maintenance, such as regulating the levels of collagen type I [105]. While skin tissue itself does not harbor tenocytes, the cells are similar in function to fibroblasts, which also produce ECM [105]. In this regard, further work can be performed in these cells to examine alterations in other cytoskeletal components and compare the outcomes to dermal fibroblasts.

*Associated Cytoskeletal Elements—Real Microgravity using Rodent Models:* Sprague Dawley rats (8-day newborn males) were subjected to real microgravity conditions aboard the STS-90 mission, and results were compared to a ground-based tail suspension model or a denervation model [106]. These studies enabled a comparison of the molecular alterations in gastrocnemius muscles across these three experimental systems via DNA microarrays followed by validation by real-time PCR [106]. The spaceflight study identified the most down-regulated transcripts that were also unique, in comparison to the tail suspension and denervation studies, which included 13 cytoskeletal markers such as alpha-actin and cytoplasmic dynein [106]. The study findings suggest that simulated and real microgravity are different with respect to the resulting cytoskeletal alterations.

*Mixed—Simulated Microgravity using Cells:* Using rat aortic smooth muscle cells subjected to a roller culture apparatus to simulate microgravity, the F-actin networks were disorganized (144 h), while vimentin RNA expression that was initially reduced at 24 h later recovered at 48 h [107]. These changes were associated with reduced motility [107]. The intermediate filament, vimentin, recovered its RNA expression over the simulated microgravity time frame. However, it remains unclear whether the protein expression of vimentin was also altered.

#### 4.1.6. Endocrine System

The thyroid gland is part of the endocrine system and is well established to control an array of bodily systems (e.g., cardiovascular, immune, amongst others) which can be altered under microgravity exposure [108]. Interestingly, the skin is also considered to be an endocrine organ since it is a site of hormone production as well as where hormones can elicit their actions [109]. To uncover the impact of microgravity on endocrine cells, studies investigating changes in the cytoskeletal network were reviewed in relevant cell types.

*Actin—Real Microgravity using Cells:* While the microgravity periods on parabolic flights aboard the Airbus A300 ZERO-G did not alter the RNA levels of actin beta 1 (ACTB) in human FTC-133 thyroid cells, actin filaments were broken down aboard the TEXUS-52 sounding rocket mission (~390 s of microgravity), as noted using the FLUMIAS live-cell imaging microscope system [110]. It is presently unclear whether protein-level changes occurred aboard the parabolic flight, and, thus, this could be examined in future efforts. In addition, whether changes in function (hormone production and/or secretion) may be altered due to changes in the actin network in these or skin cells exposed to microgravity needs further examination.

*Intermediate Filaments—Simulated Microgravity using Cells:* In human ML-1 follicular thyroid carcinoma cells subjected to RPM for up to 72 h, multicellular spheroids (MCSs) and increased vimentin protein expression along with disorganized networks were noted [111]. This finding appears similar to that from Jurkat T cells subjected to real microgravity aboard a sounding rocket [83] but differs to corneal stem cells or adipose-derived stem cells in which vimentin was decreased or elicited no change [67,68,69]. Therefore, the changes in vimentin may be cell type-specific.

*Mixed—Simulated Microgravity using Cells:* Human ML-1 follicular thyroid cancer cells subjected to simulated microgravity using RPM for up to 72 h formed multicellular spheroids (MCSs) and displayed elevated cytokeratin disorganization as well as increased alpha/beta-tubulin protein levels [112]. These results show that multiple components of the cytoskeleton are altered in the thyroid cells with simulated microgravity.

*Mixed—Real Microgravity using Cells:* Upon application of real microgravity during parabolic flight campaigns (~22 s of microgravity) aboard the Airbus A300 ZERO-G, actin localization in ML-1 thyroid cells became perinuclear, while protein levels of vimentin, tubulin, and cytokeratin 8 were increased [113]. Furthermore, the RNA levels of actin beta 1 (ACTB1) and keratin 80 (KRT80) were elevated [113]. Using human FTC-133 follicular thyroid cancer cells, real microgravity aboard the TEXUS-53 sounding rocket mission (~6 min) reduced the RNA levels of actin beta 1 (ACTB), tubulin beta (TUBB), and vimentin (VIM) [114]. While the results in the parabolic flight campaigns support the simulated microgravity study using the ML-1 thyroid cells, the sounding rocket data shows the opposite result. Despite the apparent discrepancy, the protein concentrations and localization were not addressed, preventing the opportunity for an appropriate comparison. A future study could include the relevant protein data for more precise conclusions on the effects of microgravity conditions.

#### 4.1.7. Nervous and Sensory Systems

The nervous system (i.e., function of the brain and plasticity) is markedly affected by spaceflight [115,116,117]. Specific cellular alterations include altered neuroinflammation, release of neurotransmitters, neuropeptides, changes in morphology of dendritic spines, amongst others [115,116,117]. The skin tissue contains peripheral sensory nerves which release the above-described neural factors to regulate the tissue to stressors; this pathway is termed the “brain-skin connection” [118]. In skin dermatoses such as in psoriasis and atopic dermatitis, these pathways may be altered [119]. Receptors (e.g., TRPA1) present on these nerves regulate sensations to pain, itch, and temperature changes [120]. To uncover whether microgravity impacts the nervous system (and the sensory system, i.e., vision and hearing) from a molecular standpoint, studies examining changes in the cytoskeletal network were reviewed in this context.

*Intermediate Filaments—Real Microgravity using Cells:* Aboard the ISS, application of real microgravity to human ARPE-19 retinal pigment epithelial cells for 3 days markedly altered vimentin organization from the plasma membrane to the perinuclear region [121]. This finding is consistent with altered vimentin organization in thyroid cells. Further work is needed to examine changes in vimentin across other nervous system cell types.

*Microtubules—Simulated Microgravity using Cells:* Using human primary brain-derived glioma cells, application of the 3D clinostat caused the loss of the microtubule organizing center (MTOC) with enriched microtubule perinuclear localization, as noted via cell staining for beta-tubulin [122]. This data is consistent with changes reported in other cell types.

*Microtubules—Simulated Microgravity using Rodent Models:* BALB/c mice were subjected to tail suspension for 7 days followed by hippocampal protein analyses via 2-dimensional mass spectrometry [123]. These analyses identified reduced protein levels of beta-tubulin in the hippocampi [123]. This data is also consistent with microtubule changes reported in other cell types.

*Mixed—Simulated Microgravity using Cells:* In rat glioma C6 cells subjected to RPM for up to 20 h, disorganization of actin, microtubule, and intermediate filaments increased along with elevated nuclear membrane blebbing [124]. However, at 32 h, the cytoskeletal organization was restored [124].

When human retinal pigment ARPE-19 epithelial cells were subjected to simulated microgravity using RPM for 5 and 10 days, they formed adherent monolayers (ADs) as well as multicellular spheroids (MCSs) [125]. With respect to changes in the cytoskeleton, F-actin filaments were diminished intracellularly but were prominent at the plasma membrane at 5 days; however, these changes were restored to control cell levels at 10 days [125]. Further, the RNA levels of actin beta (ACTB), tubulin beta (TUBB), vimentin (VIM), and keratin 80 (KRT8) were reduced at 5 to 10 days of simulated microgravity [125]. These findings demonstrate a recovery of the actin filament organization with long-term simulated microgravity exposure.

#### 4.1.8. Skeletal System

Bone loss is a common outcome of spaceflight [126]. This is relevant to the health of the integumentary system since they are tightly interconnected (i.e., skin–bone axis), with similarities at various levels of function [127]. Skin dermatoses can also affect the health of the bone (and vice versa) [127]. To uncover the impact of microgravity on bone cells, studies investigating changes in the cytoskeletal network were reviewed.

*Actin—Simulated Microgravity using Cells:* Simulated microgravity using the clinostat for 6 to 24 h on rat osteoblast ROS 17/2.8 cells resulted in increased cell rounding, reduced cellular viability, and elevated apoptosis [128]. Other changes included actin filament disorganization with enrichment of integrin-β1 at the perinuclear region [128]. Further work is needed to demonstrate a connection between these changes and the cellular outcomes.

*Actin—Real Microgravity using Cells:* In ROS 17/2.8 osteoblasts subjected to real microgravity aboard the Foton 11 and Foton 12 satellites, increased cell rounding (at 4 days), ablation of F-actin stress bundles (at 4 days), and vinculin disorganization (at 2 days) were observed [129]. Altogether, these study findings support the in vitro simulated microgravity study outcomes. However, how these changes mediate impact at the organismal level at the level of both bone and skin remains unclear, and how it impacts skin dermatoses needs further investigation.

*Microtubules—Simulated Microgravity using Cells:* Simulated microgravity using the RWV device for 72 h across a series of bone cell lines (i.e., human MG-63, U2OS, Saos-2, hFOB1.19, and rat osteoblasts) caused a marked reduction in the proliferative capacity with apoptotic features in hFOB1.19 and rat osteoblasts [130]. Across all these bone cell lines, altered microtubule spindles and centrosome numbers were reported [130]. In addition, in MG-63 and U2OS cells subjected to 72 h of microgravity, the cytoskeletal-associated elements BUB1 and MAD2 were increased and associated with the appearance of multipolar spindles [130]. Knockdown of BUB1 or MAD2 using shRNA reduced the multipolar spindles and recovered the proliferative capacity [130]. Both BUB1 and MAD2 are critical to support the proper segregation of chromosomes into the daughter cells during cell division; they act by stabilizing kinetochore microtubules [131]. Since these molecules are part of the basic mechanism of cell division, it would be worthwhile to examine their expression in skin cell types in response to microgravity.

*Microtubules—Real Microgravity using Cells:* In a real microgravity study aboard the Space Shuttle for 4 to 5 days, rat osteoblastic cells displayed reduced alpha-tubulin RNA expression [132]. Whether the RNA-level changes in tubulin correlates with changes in its protein expression, and organization is unclear.

*Associated Cytoskeletal Elements—Simulated Microgravity using Cells:* Using rat calvarial osteoblastic cells subjected to simulated microgravity using RPM from 6 to 24 h, cytoskeletal alterations included reduced numbers of cilia and acetylated alpha-tubulin (one major component of cilia) protein expression [133]. Recovery of these changes occurred at varying levels with lithium chloride (LiCl), cytochalasin D (cyto D), or siRNA targeting Dynlt1 (dynein light chain 1, which contributes to dynein-2 motor protein function), leading to extension of cilia lengths and increased acetylated alpha-tubulin protein levels [133]. The recovery induced by cytochalasin D, however, was opposed by siRNA targeting the intraflagellar transport 88 protein (IFT88), which shortened the cilia lengths [133]. Cilia contribute to sensing and respond to external stimuli. Further, the acetylated form of tubulin is considered to elicit increased stability and is a component of cilia [134]. With respect to skin dermatoses, loss of cilia has been reported to be associated with melanoma disease [135].

*Associated Cytoskeletal Elements—Real Microgravity using Cells:* Real microgravity application via the Foton-M3 satellite for 69 h to human MG-63 osteosarcoma cells resulted in an array of cytoskeletal alterations [136]. These changes include reduced vinculin staining and stress fibers [136]. However, these alterations dissipated upon treatment with siRNA targeting the Rac1 (a member of Rho GTPase family) but not with siRNA targeting RhoA or Cdc42 Rho GTPases [136]. Vinculin is a mechanosensor and contributes to the integrity of focal adhesions, which are needed to mediate cell–cell contacts [137]. Since targeting Rho GTPase enabled recovery from the microgravity effects, it is interesting that in skin dermatoses such as atopic dermatitis, RhoA activation is increased [138], and thus targeting this pathway may improve the health of these patients.

*Mixed—Simulated Microgravity using Cells:* In rat primary calvarial osteoblasts subjected to RPM for 24 h, the actin network remained unchanged while the microtubules were markedly depolymerized [139]. However, cellular treatment with docetaxel trihydrate (DOC) prevented depolymerization of the microtubules without changing the organization of the cilia [139]. Further, treatment with chloral hydrate (CH) or siRNA targeting the intraflagellar transport protein 88 (IFT88) reduced the cilia as well as alpha-acetylated tubulin protein levels without impacting microtubular organization [139]. Using human MG-63 osteosarcoma cells aboard the ISS, application of real microgravity for 34 h resulted in increased alpha-tubulin bundling with elevated levels of acetylated tubulin [140]. These changes occurred in the absence of F-actin alterations [140]. Further, the location of the lysosomes changed from the perinuclear region (location of the minus ends of microtubules) to throughout the cytosolic compartment [140]. The changes across these cell lines with respect to actin and microtubule organization appear consistent. The finding of lysosomal localization could be examined further to uncover its role in regulating the stability of the cytoskeletal network. In support, a prior report links microtubule targeting agents to hindering the trafficking of lysosomes [141].

Human chondrocytes isolated from healthy patients’ hip joint cartilage were also subjected to RPM for up to 24 h [142]. While there was no morphological change, beta-tubulin changed its localization pattern to the perinuclear region [142]. Vimentin was also enriched around the perinuclear region while also localized at the cell surface [142]. F-actin fibers lengthened and were thinner; the protein levels of beta-actin and beta-tubulin were increased (at 4 h), while vimentin was elevated (at 24 h) [142]. Although chondrocytes do not exist in the skin tissue, there are stem cells in the dermal layer which may differentiate into this cell type [143]. As such, it is expected that communication between them would regulate a need to generate more chondrocytes and, hence, impact the health of the skin and the cartilage tissues.

*Mixed—Real Microgravity using Cells:* When human chondrocytes were subjected to real microgravity during parabolic flight campaigns aboard the Airbus A300 ZERO-G, discontinuous regions were observed in the cytosolic compartment for tubulin, vimentin, and cytokeratin along with elevated levels of RNA and protein for tubulin beta (TUBB), vimentin (VIM), and keratin 8 (KRT8) at P31 [144]. Further, F-actin organization was rearranged, and actin beta (ACTB) protein was elevated at P31 [144]. Across the simulated and real microgravity studies, consistent impacts to a wide array of cytoskeletal targets were noted.

#### 4.1.9. Reproductive System

It has been reported that simulated microgravity can alter the outcome of pregnancy, possibly due to the failure of implantation [145]. There is also evidence that male germ cells (i.e., spermatozoa) move more slowly [146]. To uncover the impact of microgravity on cells of the reproductive system, studies focused on cytoskeletal alterations were reviewed.

*Intermediate Filaments—Simulated Microgravity using Cells:* In human breast CRL-2351 cells subjected to RPM for 5 days, increased vimentin RNA and protein were noted in both the adherent (AD) and multicellular spheroid (MCS) cultures [147].

*Intermediate Filaments—Human Models:* Human prostate explants were subjected to RWV simulated microgravity (up to 28 days) [148]. While morphological alterations included edema, condensed stroma with increased smooth muscle components with glandular elements, cytokeratins were retained throughout the acini with intense vimentin expression in stromal, acinar, and epithelial components [148].

*Mixed—Simulated Microgravity using Cells:* Using breast cancer MCF-7 cells, simulated microgravity under clinorotation for up to 7 days caused increased cell rounding, reduced F-actin bundling and microtubule networks, showed an absence of lamellipodia, and decreased vinculin regions [149]. Using prostate cancer DU145 cells, simulated microgravity applied up to 14 days using the RWV (HARV) device increased cytokeratin 8 and 18, actin, and vimentin staining [150]. While human seminoma TCam-2 cells were also subjected to simulated microgravity using the RPM device for 24 h [151], microgravity did not induce any changes in actin organization, but the microtubule organization dissipated and could be recovered at 48 h [151]. Across these cell types, varied cytoskeletal mediators were investigated, with reduced F-actin, reduced microtubule networks, and focal adhesions in one study and increased intermediate filaments in another or no change in actin but with reduced microtubules, which recovered. These varied findings may be due to the different cell types, different simulated microgravity devices, or different exposure times to microgravity.

Human J-111 cells (N.B. ICLAC database reports this line as a HeLa cell contamination) were subjected to simulated microgravity using RPM, which resulted in increased cellular roundedness. In addition, reduced F-actin networks with disorganization, microtubular reorganization to the perinuclear region, clustering of vinculin under the cell periphery (at 1 and 24 h), and diminished cellular motility (at 1 h) were noted [152]. However, restoration of F-actin organization was visible at 24 h [152]. With this same cell line, 24 h microgravity simulation with RPM increased cellular contraction with reduced movement [153]. These features were associated with reduced F-actin networks, reduced and disorganized beta-tubulin with thickened perinuclear staining, elevated vinculin staining in clusters under the cell surface, and diminished focal adhesions [153]. Using CCL-13 cells (N.B. ICLAC database also reports this line as a HeLa cell contamination) subjected to clinostat for 72 h, the cytoskeletal changes included diminished levels of beta-actin and beta-tubulin proteins in addition to accumulated microtubules at the perinuclear region and F-actin disorganization [154]. With human ONCO-DG-1 cells (N.B. ICLAC database reports this line as ovarian cancer OVCAR-3 cells) subjected to RPM, multicellular spheroids (MCSs) formed and changes in cytoskeletal elements included microtubule disorganization with loosened cytokeratin networks as early as 30 min [155]. These cytoskeletal changes were, however, recovered at 48 h [155]. Across these cell types (notated as HeLa cell contaminants), the cytoskeletal outcomes appear consistent, along with features of recovery.

*Mixed—Real Microgravity using Cells:* In prostate cancer PC3 cells, real microgravity was applied via parabolic flight campaigns aboard the Airbus A300 ZERO-G [156]. While F-actin stress fibers were increased and were associated with increased pseudopodia and lamellipodia, the RNA levels of tubulin beta (TUBB) and actin remained unchanged [156]. When MCF-7 breast cancer cells were subjected to parabolic flight campaigns aboard the Airbus A300 ZERO-G, elevated RNA levels of keratin 80 (KRT8) along with reduced vinculin (VCL) RNA and protein were observed in the absence of changes in actin beta (ACTB) or tubulin beta (TUBB) [157]. It is unclear whether these RNA-level alterations would translate into protein-level expression or changes in their localization patterns. When breast cancer MCF-7 cells were subjected to real microgravity aboard the TEXUS-54 sounding rocket mission (~6 min of microgravity), filopodia and lamellipodia structures were visible in live cells (via the FLUMIAS imaging system) along with elevated F-actin-stained regions in fixed cells [157]. When analyzed, the protein-level alterations and patterns were similar across the studies.

#### 4.1.10. In Vitro Assay

Using either magnetic levitation (3 min) or clinorotation (15 min) to investigate the effect of microgravity on self-organization of microtubules using an in vitro assay, formation of microtubules was not observed, suggesting that self-assembly under simulated microgravity conditions is disrupted [158]. While this experimental approach is an in vitro assay, the microtubule findings are consistent throughout the above organ systems using cells.

### 4.2. Protein Trafficking

**Figure 5:**
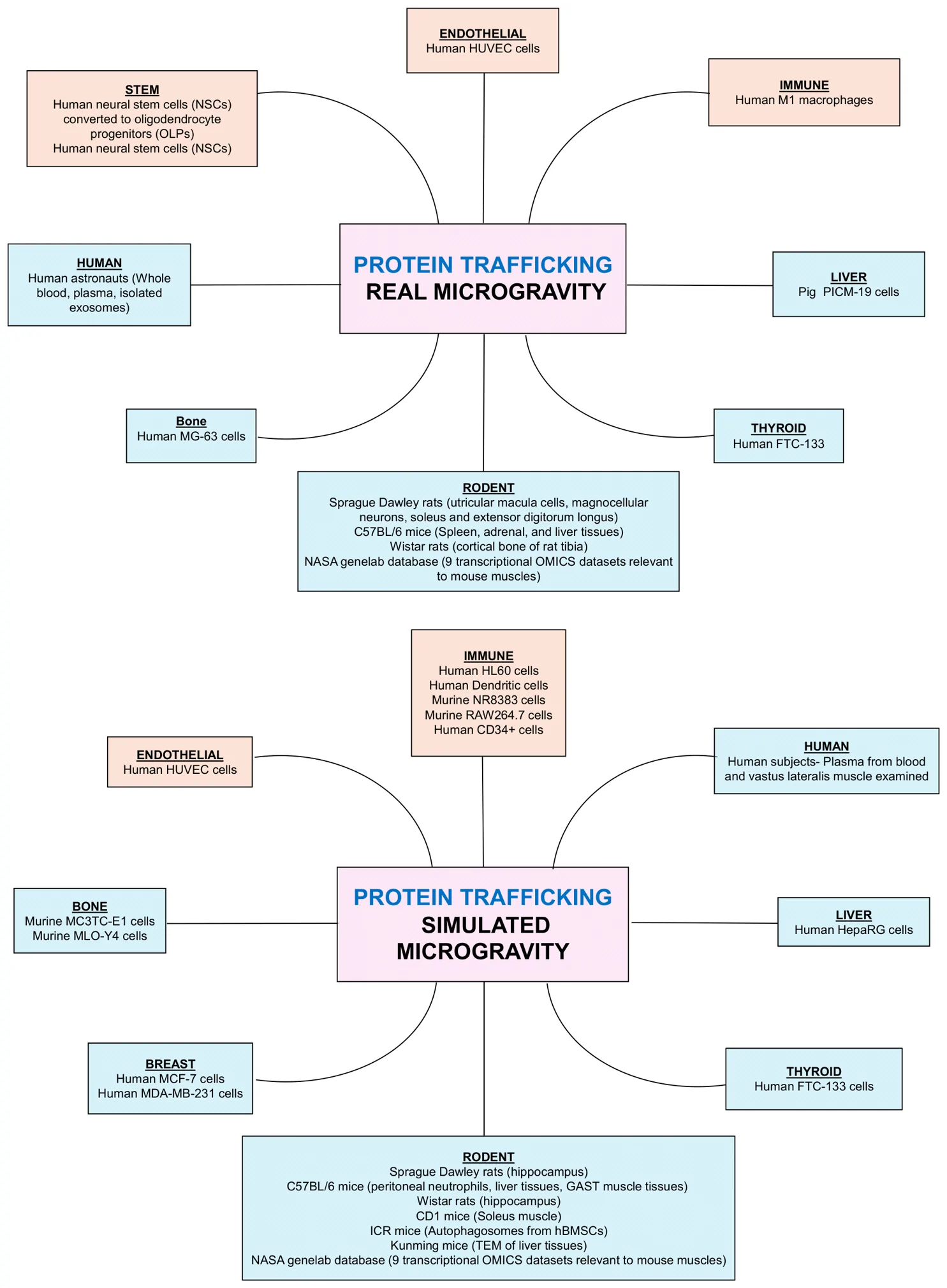
Real and simulated microgravity studies pertaining to protein trafficking. Model systems are organized according to real microgravity and simulated microgravity studies in the top and bottom panels, respectively. Specifically, model systems pertaining to the skin are displayed in green. Model systems displayed in orange are cell line models that are relevant to skin tissue, while those displayed in blue are not relevant to skin tissue.

A subset of studies uncovered through our initial PubMed searches investigated the impact of microgravity on protein trafficking, though there were no results found in skin-specific models. However, findings using skin-relevant and other models are expected to advance our understanding of how microgravity alters protein trafficking events in skin tissue. Details are summarized in Supplementary File S15, and a schematic of the experimental models used is presented in Figure 5, with the relevant citations presented in Supplementary Table S2.

#### 4.2.1. Organellar Alterations

A subset of real microgravity studies using cell lines did not show organellar alterations. For example, no notable ultrastructural changes were identified in the ER, Golgi, or mitochondria in pig liver stem cells, namely PICM-19, when subjected to 16 days of real microgravity aboard the STS-126 mission [159]. Furthermore, human M1 macrophages did not show any notable changes in the lysosomes, as noted via the FLUMIAS real-time imaging fluorescence platform, when subjected to real microgravity (~353 s) aboard the TEXUS-54 sounding rocket mission [82]. However, lysosomal changes were noted in human MG-63 osteosarcoma cells subjected to real microgravity aboard the ISS for 34 h [140]. Specifically, LAMP2 (a regulator of lysosomal pH) redistributed from the perinuclear region (at minus ends of the microtubules) to the cytosolic compartment (which includes the plus ends of microtubules); this was determined via immunofluorescence staining to assess lysosomal localization patterns [140].

In simulated microgravity studies using rodents, organellar alterations were noted in male CD1 mice subjected to simulated microgravity via hindlimb suspension up to 48 h; specifically, they displayed heterophagosomes in the soleus muscle fibers [160]. In real microgravity studies using rodent models, some alterations were noted. Male Wistar rat tibia under 12.5 days of real microgravity aboard the Cosmos 1887 spaceflight showed organellar changes in the tibial osteoblastic cells, including increased Golgi intermediate vesicles along with NADPase activity in the proximal tibia [161]. However, aboard the STS-51B (Spacelab 3) mission, in Sprague Dawley male rats subjected to real microgravity for a period of 7 days to examine the ultrastructural alterations due to muscular atrophy, the soleus fibrils did not contain autophagosome-lysosomal characteristics that could explain the myofibril degradation [162]. In another study, female Sprague Dawley rats subjected to 16 days of real microgravity aboard the STS-90 mission showed increased numbers of endoplasmic reticulum, Golgi, lysosomes, and mitochondria with reduced numbers of neurosecretory vesicles, specifically within the supraoptic neurons [163]. Further, type I and type II cells of the inner ear tissues from Sprague Dawley rats contained disorganized, rough endoplasmic reticulum (RER) upon landing of the SLS-2 14-day spaceflight; however, these changes were absent on day 13 post-landing [164]. Together, simulated and real microgravity studies identified organellar changes across cell lines and rodent models, which in one study were shown to return to normal conditions upon landing.

Since these organellar changes included the ER and the Golgi apparatus, which are involved in post-translational modifications such as glycosylation and are actively involved in the secretory pathway (e.g., vesicular transport), the effect of microgravity on these components was reviewed (Section 4.2.2 and Section 4.2.5, respectively). Furthermore, while the mechanisms that underlie the changes across the above-described studies remain unclear, alterations in the ER stress/autophagic response and/or phagocytosis could contribute to these changes, which are addressed in Section 4.2.3 and Section 4.2.4. In addition, since none of the above organellar alterations were examined in skin tissue, this research area can be addressed in the future.

#### 4.2.2. Glycosylation

A number of proteins in skin tissue involved in skin barrier function are post-translationally modified via palmitoylation [165]. However, this area appears to be one that is understudied with respect to microgravity exposure. As such, other types of modification, such as glycosylation, were instead reviewed. To address the effect of real microgravity on glycosylation, nine transcriptional datasets available through the Genelab Database derived from the NASA Rodent Research 1 (RR1) mission were investigated for genes relevant to glycosylation, specifically for extensor digitorum longus, quadriceps, soleus, tibialis anterior, longissimus dorsi, and gastrocnemius [166]. These studies identified that the soleus muscle contains the highest levels of differentially expressed genes (DEGs) relevant to glycogenes involved in N-glycosylation and glycosaminoglycan metabolism [166]. However, further work is needed to validate the impact on the glycosylated targets and cell function.

Glycosylation was also assessed in simulated microgravity studies using cell lines. Human thyroid FTC-133 and human breast MCF-7 cancer cells were subjected to RPM for 3 and 14 days, respectively, followed by mass spectrometry [167]. While further experimental validation is needed, the analyses uncovered proteins with potential glycosylation features, including cofilin-1 and Rab27B [167]. In another study using these cells, proteins identified included those involved in adhesion, which contain potential sialylation features (i.e., CDH1, NCAM-1, CD44, ITGB1, ITGB4, ITGB3, and ITGB5) [168]. In addition, molecules that were identified in the analyses that contribute to this post-translational modification included SLC35A1, ST3GAL1, CAV1, amongst others [168]. However, further experimental effort is needed to validate whether changes in these post-translational modifications actually occur on specific targets during microgravity as well as examine the functional impact of these changes on cellular and tissue health.

#### 4.2.3. ER Stress and Autophagy

The ER is a key subcellular location for protein production, protein folding, and protein processing, which are regulated by signaling pathways [169]. The cell can counter the accumulation of misfolded proteins by inducing the ER stress pathway, which engages the unfolded protein response (UPR) [169]. Sensors to detect misfolded proteins are located on the ER membrane (i.e., IRE1α, PERK, and ATF6), which normally are kept inactive by BiP, an ER chaperone [169]. Stimuli that may activate these pathways include ultraviolet radiation, psychological stressors, temperature changes, as well as irritants of the skin [169]. ER stress can be connected to autophagy, a lysosome-degradative pathway that is involved in clearing misfolded proteins as well as damaged organelles [170]. Autophagy is recognized to play an essential role in skin homeostasis, including skin barrier function [171]. Several skin dermatoses are characterized by ER stress, including rosacea and epidermolysis bullosa simplex [169,172]. Alterations in the autophagy pathway have also been reported in skin dermatoses, such as psoriasis and atopic dermatitis [171]. To uncover the impact of microgravity on ER stress and associated pathways, studies utilizing an array of model systems were reviewed.

##### Cardiovascular System

*Simulated Microgravity using Cells:* HUVECs subjected to clinorotation for 48 h displayed changes in autophagy, including increased GFP-LC3 punctae, elevated numbers of double-membrane autophagosomes via transmission electron microscopy (TEM), increased lipidated LC3-II via Western, as well as increased beclin-1 RNA and protein expression [173]. In another study, these cells that were subjected to 2D clinostat for 48 h displayed elevated autophagic flux characterized by increased LC3-II and reduced p62 proteins as well as increased apoptosis and features of ER stress (i.e., increased protein levels of p-PERK, GRP78, CHOP, p-EIF2S2) [174]. Inhibition of autophagy using 3-methyladenine (3-MA) under these conditions increased the apoptotic response, suggesting that autophagy mediates protection to support cell survival [174]. In support, knockdown of ATG5 using siRNA also increased ER stress, unfolded protein response (UPR), as well as apoptotic features [174]. Furthermore, inhibition of ER stress using 4-phenylbutyric acid (4-PBA) increased autophagic flux and reduced apoptotic response [174]. In an independent study, endothelial HUVEC cells subjected to 2D clinostat for 72 h also displayed markers of ER stress (i.e., GRP78 and CHOP), and use of 4-PBA or tauroursodeoxycholic acid (TUDCA) reduced ER stress features and apoptosis [175]. Altogether, the autophagy pathway appears to elicit protection against the ER stress-induced apoptosis to support the cell survival response in endothelial cells. Moreover, targeting the ER stress pathway improves cell survival.

##### Muscular System

*Simulated Microgravity using Rodent Models:* In C57BL/6J mice with plasminogen gene deficiency (Plg-/-) that were subjected to simulated microgravity (hindlimb unloading for 21 days), beclin-1 RNA as well as LC3B RNA and protein were elevated in their gastrocnemius muscles [176]. This supports prior findings of an association between fibrinolysis and muscle atrophy [176]. In another study, hindlimb unloading applied to ICR mice for 14 days resulted in elevated numbers of autophagosomes as well as autophagy markers (i.e., Beclin-1, BNIP3, ATG7) in soleus muscles [177]. These autophagy changes could be reversed by extracellular vesicle (EV) treatment during hindlimb unloading; these EVs were derived from human bone marrow mesenchymal stem cells [177]. Across these rodent studies, it is unclear whether ER stress was induced and whether autophagy is activated as a protective measure.

*Human Participants:* In humans subjected to dry immersion as a form of simulated microgravity, reduced RNA expression of autophagy markers including ATG5, GABARAPL1, and BNIP3 in the right vastus lateralis muscle was reported [178]. These autophagy changes were also associated with reduced signaling, specifically in p-4EBP1, p-RPS6, and RPS6 protein levels [178]. Since the autophagy markers were only measured at the RNA level, it is unclear whether the protein levels of these autophagy mediators were altered. Furthermore, the status of the ER stress pathway is unclear.

##### Digestive System

*Simulated Microgravity using Rodent Models:* Kunming mice were tail suspended to simulate microgravity for 2 and 4 weeks [179]. Their liver tissues were characterized by increased autophagosomes and reduced numbers of mitochondria [179]. When male C57BL/6J mice were subjected to hindlimb unloading for 21 days, ER stress features (i.e., ATF-4, CHOP, XBP1) increased at the RNA level in their liver tissues [180]. However, treatment with the ER stress inhibitor 4-PBA reversed the increase in these ER stress markers, including detrimental effects on liver health [180]. The ER stress and autophagy findings presented for liver tissues support those reported for endothelial cells. Further, targeting the ER stress pathway could improve liver health.

##### Nervous System

*Real Microgravity using Cells:* Human neural stem cells converted to oligodendrocyte progenitors (OLPs) were subjected to real microgravity aboard the ISS (BioScience-4 mission) for 20 h [181]. Cellular changes included increased autophagy as well as markers of ER stress (i.e., SPARC and HSP90) in the secretome of the OLPs [181]. In a separate study, human neural stem cells subjected to real microgravity aboard the ISS for 39.3 days displayed increased autophagy-like features (1 week after return to Earth) along with elevated ER stress markers in its secretome (i.e., SPARC and HSP90β) [182]. These findings using neural stem cells also demonstrate that ER stress and autophagy are elevated with microgravity exposure.

##### Skeletal System

*Simulated Microgravity using Cells:* Towards the goal of elucidating simulated microgravity-induced changes during osteoclastogenesis, murine RAW264.7 macrophages (considered as pre-osteoclasts) were applied to RCCS for 24 h under RANKL stimulation [183,184]. Autophagy activity, as measured using acridine orange, was elevated with RANKL stimulation, compared to control 1g cells, and was associated with increased localization of syncytin-A, which elicits a fusogenic role in osteoclast formation [184]. Targeting syncytin-A with siRNA prevented autophagic activity in addition to osteoclast formation [184]. While microgravity with RANKL cellular stimulation led to elevated levels of p62 protein and lysosomes (and their colocalization), they were both reduced by MG132 (a proteasome inhibitor) in these pre-osteoclasts [183].

Murine MC3T3-E1 pre-osteoblasts subjected to clinostat between 24 and 72 h displayed increased LC3-II protein, which colocalized with LAMP2, a lysosomal marker [185]. In addition, simulated microgravity caused reduced activation of signaling pathways (i.e., p-ERK, p-AKT, p-mTOR) with elevated ER stress markers (i.e., GRP78 and p-PERK) [185]. The antioxidant melatonin (used at doses of 100–200 nM) supported recovery from these microgravity-induced cellular alterations [185]. This data is supported by the findings reported above in other tissue systems.

#### 4.2.4. Phagocytosis

Interestingly, the process of autophagy can influence phagocytosis [186]. Immune cells such as macrophages are common residents of skin tissues but also infiltrate the skin in response to external stimuli such as cytokines [187]. Deregulated macrophage functions are well established to contribute to skin dermatoses [187]. Specifically, macrophages play roles in eliminating microbes but also are essential players in the inflammatory response [187]. With respect to the former function, macrophages are involved in engulfing matter via the process of phagocytosis. As such, macrophages can not only clear microbes but also dead and dying cells [188]. Other cells of the immune system which undergo phagocytosis include neutrophils as well as dendritic cells.

*Simulated Microgravity using Cells:* Human dendritic cells were subjected to simulated microgravity using the RCCS device for 9–12 days [189]. The cells displayed reduced phagocytotic response towards *A. fumigatus* [189]. Further, the dendritic cells expressed reduced expression of IL-12, HLA-DR, as well as CD80, CD86, and CD54 proteins in response to *C. albicans* [189]. When rat NR8383 macrophages were subjected to 2D clinostat, the cells elicited reduced phagocytotic response within 30 min of exposure to fluorophore (FITC)-labeled zymosan; however, the activity was recovered after 60 to 180 min [190]. Both independent studies, using either dendritic cells or macrophages, support simulated microgravity-induced reduction in phagocytotic activity.

*Simulated and Real Microgravity using Rodent Models:* In neutrophils isolated from male C57BL/6 mice subjected to tail suspension for 14 days, phagocytosis activity towards *E. coli*, *S. aureus*, and zymosan remained unchanged [191]. However, in a real microgravity study aboard the STS-135 space shuttle Atlantis, splenocytes isolated from C57BL/6 mice elicited elevated phagocytotic activity to bead microspheres in addition to increased IFN-γ secretion and reduced IL-2 levels [192]. In contrast, aboard the space shuttle Atlantis for 13 days, splenocytes isolated from female C57BL/6J mice displayed reduced phagocytotic activity towards *S. aureus* [193]. Across these three rodent model studies, there was no change under the simulated microgravity study using neutrophils isolated from tail suspended mice, and splenocytes in one real microgravity study showed increased phagocytosis, while the other had the opposite response. However, since the microgravity exposure time and stimuli varied, this may have impacted the functional outcomes reported.

*Human Participants:* Human males were subjected to parabolic flights aboard the Airbus A300 ZERO-G for 30 campaigns [194]. Although polymorphonuclear leukocytes (PMNs) were isolated at various time points and their numbers were elevated, they did not display a change in their phagocytotic activity in response to fluorophore-labelled zymosan [194]. However, the PMNs did increase their levels of secreted IL-8 and G-CSF proteins after the parabolic flight [194]. In this study, microgravity exposure time is short and may not be long enough to induce changes in the phagocytotic response. In the future, phagocytosis could be examined in immune cells isolated from astronauts on lengthier spaceflights immediately post-landing or across a time course.

#### 4.2.5. Intracellular Movement of Targets, Vesicles, and Secretion

Since organelle, ER stress, and autophagy alterations were noted with microgravity exposure, it is expected that intracellular movement of macromolecules, movement of vesicles, and exocytosis events may also be altered. Thus, microgravity-induced changes across these trafficking events were reviewed across different model systems. Further, a subset of studies included exosomes or extracellular vesicles (EVs). These are reported to contribute to skin disease pathogenesis as well as elicit therapeutic benefit, such as improving skin barrier function and remodeling damaged tissue [195].

##### Immune System

*Simulated Microgravity using Cells:* In human promyelocytic HL60 leukemia cells subjected to RWV for 48 h as a mode of simulated microgravity, vascular endothelial growth factor receptor-2 (VEGFR-2) became re-localized from the nuclear compartment to the cell surface [196]. In human hematopoietic stem progenitor CD34+ cells that were also subjected to RWV (for 72 h), VEGFR-1, VEGFR-2, and VEGFR-3 were all re-localized from the nuclear compartment to the plasma membrane [197]. In both studies, these protein trafficking changes were associated with reduced cellular survival [196,197]. Future work can address the underlying mechanism for VEGF receptor re-localization, its activation status, and the downstream signaling cascades that lead to diminished cell survival.

*Human Participants:* Exosomes were isolated from astronauts 10 days prior to launch and 3 days after landing from a 14-day spaceflight [198]. Within exosomes at 3 days post-landing, oxidative stress and RNA expression of markers of inflammation were noted to be elevated (i.e., TNF-α, IL-1α, and IL-1β) [198]. Whether these RNA changes in cytokine expression translate into changes in protein expression and secretion is unclear.

##### Cardiovascular System

*Real Microgravity using Cells:* Human endothelial HUVEC cells were subjected to real microgravity aboard the ISS for 10 days [199]. Secretome analysis of these cells uncovered increased levels of IL-1α and IL-1β in the culture media along with reduced trends of IL-10 and VEGF levels [199]. Based on this data, we expect that the secretome may be altered in endothelial cells derived from skin.

In another study, the EV proteome was assessed across pre-flight and post-flight times from the SpaceX Inspiration 4 (i4) 3-day mission [200]; the findings showed shared commonalities to the plasma proteome and included increased PRDX2 and SCNA with reduced levels of ICAM3 post-flight [200]. Additional studies could be performed to assess the functional implications of EV presentation on skin cells.

##### Nervous System

*Simulated Microgravity using Rodent Models:* Male Wistar rats subjected to hindlimb unloading for 3 days displayed increased levels of hippocampal p-synapsin I, which is involved in vesicle exocytosis in its activated form; increased p-synapsin I was associated with elevated p-PTEN and reduced p-AKT (Ser473) protein levels [201]. Application of dynamic foot stimulation (DFS) to this simulated microgravity model improved hippocampal function [201]. This simulated microgravity study supports increased exocytotic outcomes in the hippocampus.

*Simulated Microgravity Using Rodent Models:* The levels of neurotransmitters in hippocampi were altered in male Sprague Dawley rats subjected to simulated microgravity via tail suspension for 21 days [202]. In addition, proteins involved in vesicular transport were altered, including reduced levels of VAMPA3, Rab3A, syntaxin-1A, and syntaxin-binding protein 5, with increased levels of PSD-95 and VGLUT1/2 [202]. Thus, changes in vesicular transport appear associated with alterations in neurotransmitter secretion.

##### Endocrine System

*Simulated Microgravity using Cells:* Increased numbers of exosomes were noted in human FTC-133 thyroid cancer cells subjected to real microgravity aboard the ISS for 12 days; the exosomes were characterized by elevated CD63 and CD81 tetraspanins [203]. The functional outcomes of increased exosomes remain to be determined.

##### Digestive System

*Simulated Microgravity using Cells:* Human immortalized hepatic HepaRG cells that were subjected to clinostat for 3 days with the rotating flat chamber device elicited increased differentiation features characteristic of hepatocyte-like cells including albumin (ALB), cytokeratin 18 (CK18), and alpha-fetoprotein (AFP) at the RNA and protein levels [204]. A compound library containing 206 mechanical receptor-sensitive inhibitors identified ML-SI1, which could revert this response by targeting TRPML1, a cation channel [204]. In terms of signaling pathway changes, simulated microgravity increased YAP translocation and β-catenin movement into the nuclear compartment [204]. Further, β-catenin movement could be opposed by BAPTA-AM (a calcium chelator) and YM201636 (a PI(3,5)P_2_ inhibitor) [204]. These studies show that protein trafficking is altered by simulated microgravity. YAP and β-catenin are part of the Hippo and Wnt pathways, respectively, and elicit roles as mechanosensors in skin tissue [205]. Further, mechanical stimuli can activate currents in skin cells (i.e., keratinocytes) via ion channels such as TRP channels [205]. While this study utilized HepaRG cells, studies could be performed to examine similar outcomes in cells relevant to skin tissue and in various skin disease conditions.

##### Skeletal System

*Simulated Microgravity using Cells:* Murine MLO-Y4 osteocytes were subjected to RPM for 2 h and found to elicit reduced expression of connexin 43 (Cx43, involved in mechanical sensing via formation of gap junctions) at the cell surface with a corresponding increase in the Golgi apparatus, while the total Cx43 protein levels were unchanged [206]. These changes are relevant to skin health, as connexin has been shown to contribute to the differentiation of keratinocytes, modulation of healing of wounds, as well as skin disease pathogenesis [205]. Thus, similar studies could be performed using skin-relevant cell types to examine the impact of Cx43 and other connexins on cellular health.

##### Reproductive System

*Simulated Microgravity using Cells:* Exosomes from human MCF-7 breast cancer cells subjected to RPM for 5 and 10 days displayed increased size (at 5 days) along with increased detection of tetraspanin; CD9 was dominant with colocalization with CD63 and CD81 [207]. In another study, human breast cancer MDA-MB-231 cells were subjected to simulated microgravity using the Gravite device (from 96 h up to 2 months). The cells showed increased cell roundedness, which was associated with increased doubling time and reduced migratory capacity [208]. While CD81 and TSG101 were elevated at the cell surface, these markers were reduced in extracellular vesicles (EVs), which became larger in size [208]. Further, increased levels of GTPases (i.e., RalA, RalB, RhoA, RhoC, Cdc42, and Rab13) were identified at the protein level via a proteomics analysis of the EVs [208]. While protein-level changes were noted in exosomes and EVs, further work would be valuable to determine their functional relevance.

### 4.3. Signaling

**Figure 6:**
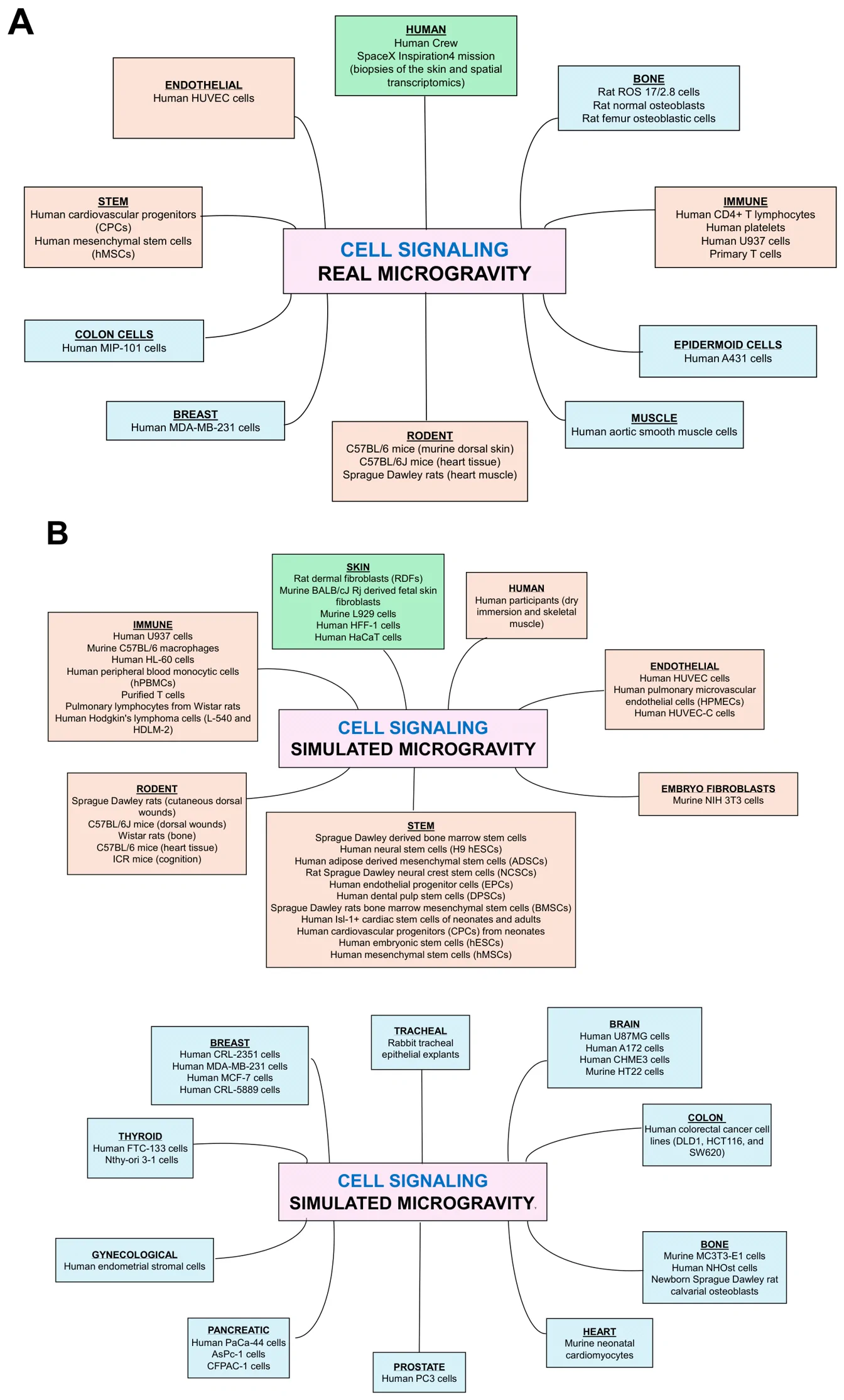
Real and simulated microgravity studies pertaining to cell signaling. Model systems are organized according to real microgravity and simulated microgravity studies in panel (**A**) and (**B**), respectively. Specifically, model systems pertaining to the skin are displayed in green. Model systems displayed in orange are cell line models that are relevant to skin tissue, while those displayed in blue are not relevant to skin tissue.

A subset of studies uncovered through our initial PubMed searches investigated the impact of microgravity on specific cell signaling cascades in skin-specific models. As for cytoskeleton and protein trafficking, findings using skin-relevant and other models are also expected to advance our understanding of how microgravity alters signaling events. Furthermore, these findings can improve our understanding of the pathogenesis of various skin dermatoses. Details of these studies are summarized in Supplementary File S16, and a schematic of the experimental models used is presented in Figure 6, with the relevant citations presented in Supplementary Table S3.

#### 4.3.1. Integumentary System

*Simulated Microgravity using Cells:* Rat dermal fibroblasts were subjected to simulated microgravity using the RPM device from 30 min to 48 h [52]. The cells acquired surface ruffles with spindle-like features while the F-actin displayed increased bundling (within 30 min) with thinning and increased focal adhesions at 48 h [52]. Other cytoskeletal element alterations included elevated levels of Rho family GTPases such as RhoA, Cdc42, and Rac1 proteins [52]. With respect to signaling events, p-ERK1/2 and total ERK1/2 were reduced, while p-JNK1/2, total JNK1/2, p-p38, and total p38 were increased [52]. This study reports findings that demonstrate alterations in signaling pathways as well as cytoskeletal changes. Knockdown and overexpression studies could be performed in the future to determine the contribution of the signaling events to the cytoskeletal alterations.

In another study, murine fetal skin fibroblasts were subjected to RPM for 65 h and found to display increased RNA expression of oxidative stress genes (i.e., Gsta1, Gsta2, Hmox1), reduced RNA levels of cytoskeletal markers (i.e., Actg2, Acta1, Cnn1, and Fhl1), and elevated RNA expression of MAPK signaling negative regulatory mediators (i.e., Dusp4) [53]. While the RNA-level changes in cytoskeletal mediators and MAPK signaling pathways are reduced, it is unclear whether the protein-level changes across these molecules would reflect a similar pattern. However, in another study, RNA and protein expression changes were correlated. Murine L929 fibroblasts, derived from subcutaneous tissue, were subjected to RCCS (HARV) up to 5 days and found to elicit reduced viability that was associated with G1 arrest and apoptotic features [209]. These outcomes were associated with reduced RNA and protein expression of Col I, Col III, and TGFβ1 [209].

The effect of simulated microgravity on the differentiation capacity of rat-derived bone marrow stem cells to dermal fibroblasts was tested via the 2D clinostat for 7 days [210]. Differentiation markers that were reduced at the RNA level included collagen I (Col I), collagen III (Col III), desmin, and fibroblast-specific protein 1 (FSP-1) [210]. Further, reduced levels of signaling mediators such as β-catenin and p-ERK1/2 proteins (relative to total ERK1/2) were noted [210]. However, cellular treatment with lithium chloride (LiCl) recovered these differentiation markers and β-catenin expression while tert-butylhydroquinone (tBHQ) treatment recovered p-ERK1/2 and β-catenin protein levels [210]. While these drugs recovered some aspects of the simulated microgravity-induced change, it is expected that overexpression studies for these signaling mediators may also reverse the microgravity-induced changes.

Human HFF-1 foreskin fibroblasts subjected to 2D clinostat from 24 to 36 h elicited reduced migratory capacity in the absence of proliferation changes [56]. Signaling changes included reduced YAP protein with increased p-YAP levels, which trafficked to both the cytoplasmic and nuclear compartment instead of only the nucleus (as noted in 1 g controls) [56]. The mitogenic lipid, LPA, restored cellular migration in wound healing assays and hindered the microgravity-induced changes in YAP [56]. JASP, an inducer of actin polymerization and stability, restored cellular migration and YAP protein under these microgravity conditions [56]. The mechanisms that underlie the reduced YAP and increased p-YAP levels could be investigated in a future study.

*Simulated and Real Microgravity using Rodent Models:* Sprague Dawley female rats with dorsal cutaneous wounds were subjected to simulated microgravity via hindlimb suspension for a period of up to 6 days; they showed reduced healing response with diminished migration of fibroblasts into the wounded region [56]. While the wound healing response was restored at 8 days, LPA promoted wound healing with increased fibroblast numbers in the wounded area [56].

Healing time from dorsal wounds in C57BL/6J male mice subjected to hindlimb unloading for 7 to 14 days was increased, and wounds were characterized by reduced epithelialization, proliferation (assessed via Ki67), and neovascularization (assessed via CD31) [211]. Further, RNA sequencing uncovered altered expression of genes involved in skin development and skin barrier function, including PI3K/AKT, MAPK, JAK/STAT, amongst others [211]. However, low-level light therapy (LLLT) improved the healing response in this mouse model subjected to simulated microgravity as well as improved cellular viability and migratory response of human HaCaT and murine NIH 3T3 cells when applied to RSOC (HARV) simulated microgravity conditions for 48 h [211].

C57BL/6 female mice, flown on the STS-135 mission for 13 days, displayed elevated RNA expression of antioxidant genes (e.g., Gsr, Ptgs2, Prdx6-ps1) and oxidative markers (i.e., Fmo2, Cat, Als2), which contribute to regulation of signaling cascades in the dorsal skin [212]. In another study, Sprague Dawley rats subjected to hindlimb unloading for 14 days followed by wound formation and infection with *E. coli* showed improvement in health when treated topically with extracts derived from *Dracontomelon dao* (*D. dao*), which included reduced activation of p-p65, p-ERK1/2, p-p38, p-SAPK/JNK, and p-AKT in the absence of change in total ERK1/2 and AKT [213].

Altogether, the use of both cellular and rodent models identified several potential countermeasures to the effects of microgravity exposure, which may lead to maintaining skin health in spaceflight and improving clinical outcomes of skin dermatoses. These include LiCl, tBHQ, LPA, JASP, and LLLT, some of which could be applied to improving wound healing responses.

*Human Participants:* Aboard the SpaceX Inspiration 4 (i4) mission, human crew were subjected to real microgravity for 3 days, in which skin biopsies were collected pre-flight and post-flight from the deltoid region [79]. While there was a lack of architectural tissue change across the outer and inner epidermis, outer dermis, and vasculature, there was elevated KRAS signaling components and reduced gene signatures of cells relevant to the skin and immune system (i.e., T cells, pericytes, fibroblasts, and melanocytes) [79]. Pathway analysis for skin barrier function uncovered reduced RNA expression of filaggrin (FLG), occludin (OCLN), claudin (CLDN), and transglutaminase 2 (TGM2) across the outer epidermis, which coincided with increased gene signatures for vascular remodeling events [79]. While protein-level expression was not addressed for these genes, these mediators play critical roles in skin health, and, thus, their deregulation may contribute to skin dermatoses.

#### 4.3.2. EGFR

Only a few studies investigated microgravity-induced changes on EGFR, a key receptor found on the surface of epithelial cells. For example, human MIP-101 colon cancer cells subjected to real microgravity aboard the STS-70 and STS-85 missions for 10 and 12 days, respectively, did not display changes in EGFR expression, although reduction in EGFR was noted in a rotation control compared to 1 g controls and could be rescued upon removal of rotation [214]. In another study, rat ROS 17/2.8 osteosarcoma cells subjected to real microgravity via parabolic flight aboard the Airbus A300 ZERO-G (~22 s of microgravity) were analyzed for changes in EGF ligand binding to its cognate receptor, EGFR; however, the cells did not display any change in EGF receptor binding to the EGF ligand [215]. In an independent study, normal rat osteoblasts subjected to real microgravity aboard the Shuttle Spacelab mission for 4–5 days also did not display changes in EGFR expression at the RNA level, while PDGF-βR, Shc, and c-fos RNAs were reduced [216]. Although these studies did not identify EGFR changes at the RNA or protein level, the activation status and localization pattern of EGFR could be examined in the future. Furthermore, it is well established that EGFR is critical to keratinocyte function [217]. Thus, microgravity-induced changes in EGFR and downstream signaling cascades could be studied using skin-relevant cell types.

#### 4.3.3. AC-PKA

In melanocytes, the melanocortin 1 receptor (a G-protein coupled receptor) signals through adenylyl cyclase (AC) to regulate intracellular cAMP levels [218]. This pathway contributes to the production of melanin, which is essential for skin pigmentation [218]. In addition, prolonged phosphodiesterase (PDE), which serves to reduce cAMP levels, can sustain the inflammatory response, contributing to the pathogenesis of psoriasis and other skin diseases [219]. Furthermore, AC (soluble forms) can promote inflammation and has been suggested as a target for therapy of psoriasis [220]. As such, mediators such as AC and PDE have been reviewed with respect to the effect of microgravity on their expression and activities across various tissue systems.

##### Cardiovascular System

*Simulated Microgravity using Rodent Models:* Ventricular myocytes were isolated from male Sprague Dawley rat heart tissue after being subjected to tail suspension for 4 weeks [221]. Gs-α protein expression remained unaltered, while adenylyl cyclase (AC) activation via forskolin stimulation led to reduced cAMP levels and contraction of the myocytes [221]. In another study, male Wistar rats subjected to hindlimb unloading for 7 days showed changes in transcript levels of AC (i.e., AC6, AC1, AC3, AC4, AC5, AC9 increased; AC7 reduced) and phosphodiesterase (i.e., PDE1A, PDE1B, PDE1C, PDE3B, PDE4D, PDE5A, PDE7A, PDE7B, PDE9A reduced; PDE1A, PDE4A, PD4B, PDE4C, PDE6D, PDE8A, PDE10A increased) in ventricular cardiomyocytes, as determined via RNA sequencing [222]. While the latter study comprehensively analyzed various transcript levels of AC and PDE, the protein-level alterations were not assessed. Thus, the functional impact of the RNA-level changes remains unclear.

*Real Microgravity using Rodent Models:* Male Sprague Dawley rats aboard the STS-51B mission (Spacelab-3) elicited reduced PDE activity (with high cyclic Km) in the heart muscle tissue [223]. Since reduced PDE activity was noted, it is expected that there may be a change in the level of cAMP, which may alter downstream signaling (via protein kinase A (PKA)) and function.

##### Digestive System

*Real Microgravity using Rodent Models:* Ultrastructural analysis of sublingual salivary glands, however, did not identify significant differences in PKA immunoreactivity in C57BL/6 mice aboard the STS-131 or STS-135 missions (15-day and 13-day, respectively) [224]. The levels of cAMP and PDE as well as AC activity could be assessed to advance our understanding of this signaling pathway.

##### Nervous System

*Simulated Microgravity using Cells:* Human neural stem cells subjected to RCCS for up to 72 h were analyzed for alterations in AC–PKA signaling [225]. Specifically, increased levels of cAMP and PKA/CREB RNA as well as protein (including p-CREB levels) were noted under these conditions [225]. These changes were associated with increased viability and mitochondrial mass [225]. The changes identified in this study suggest that the cAMP–PKA signaling may contribute to increased viability. A future study could be designed to genetically manipulate these signaling molecules to define their contributions to regulating cell survival and metabolic alterations.

##### Skeletal System

*Simulated Microgravity using Rodent Models:* Analysis of the bone matrix of femurs from female Wistar rats that were subjected to hindlimb suspension for 28 days uncovered reduced levels of p-PKA and p-CREB (with no change in their total proteins) and reduced adenylyl cyclase isoform 10 (AC10) protein [226]. Further, these molecular changes were associated with physiological alterations including reduced body weights, reduced bone mineral density (BMD), and increased adipocytes in the femurs and vertebrae [226]. Additionally, pulsed electromagnetic fields (PEMFs) recovered changes in the AC–PKA–CREB pathway as well as BMD and adipocyte levels [226]. These changes differ to those reported using neural stem cells, and, thus, stimulated microgravity may exert different outcomes in different cell types.

#### 4.3.4. JAK–STAT

The JAK–STAT signaling cascade is essential in the cellular response to cytokines, which are involved in the pathogenesis of skin diseases [227,228]. JAK inhibitors have been approved as therapy for atopic dermatitis, psoriasis, as well as other skin conditions [227,228]. However, only one study was uncovered examining changes in the JAK–STAT pathway due to microgravity. Specifically, human endothelial HUVEC cells subjected to real microgravity aboard the ISS for 12 days elicited sustained levels of STAT RNA expression up to 3 months post-landing, in addition to elevated IL-6 and ICAM-1 proteins [229]. Protein-level changes in STAT were not addressed, however. Nonetheless, further research in this area would be valuable with respect to microgravity-induced changes in skin tissues.

#### 4.3.5. MAPK

Microgravity-induced changes in the MAPK signaling network were reviewed since this pathway plays an important role in skin health. External stimuli can overstimulate the MAPK cascade, leading to breakdown of the ECM and overproduction of pro-inflammatory cytokines [230]. Such changes can lead to altered skin barrier function, reduced wound healing, and skin cancers [230].

##### Stem Cells

*Simulated Microgravity using Cells:* Human adipose-derived mesenchymal stem cells were subjected to simulated microgravity using the RCCS device for 21 days [231]. In response to TGFβ, chondrogenesis differentiation was elevated, as noted via increased RNA expression of collagen type II alpha 1 chain (COL2A1), aggrecan, Sox9, and collagen type X alpha 1 chain (COL10A1) [231]. Further, in response to TGFβ stimulation, p-p38 levels were increased under simulated microgravity in the absence of change in its total protein [231]. However, these changes could be opposed by a p38 MAPK inhibitor, SB203580, which not only inhibited p-p38 activation but reduced expression of markers of chondrogenesis [231].

Neural crest stem cells isolated from Sprague Dawley rats subjected to RCCS for 12 to 48 h showed reduced expression of neural crest markers including FoxD3, Sox10, and Snail with increased expression of the Tuj1 neurite marker [71]. Further, apoptotic features were increased (i.e., cleaved caspase) along with cytoskeletal disorganization (i.e., F-actin filaments, reduced vimentin, and increased beta-actin levels); these changes occurred alongside elevated p-p38 (T180/Y182) levels at 24 h [71]. However, pertussis toxin (PTX) or siRNA targeting CXCR4 (a regulator of neural crest cell migration) opposed these changes [71].

When human dental pulp stem cells were subjected to 3D clinostat for 3 days, functional changes included increased S phase population, proliferation, colony formation, and reduced senescence [232]. Further, increased differentiation outcomes were noted, including those for osteogenic, adipogenic, chondrogenic, and neurogenic alterations [232]. Elevated p-ERK and p-p38 levels were also observed in the absence of changes in p-JNK (and no change in their total proteins) under these simulated microgravity conditions [232].

Across all the above studies, the increase in the p-p38 levels appears consistent across the varied simulated microgravity studies and stem cell types. The contribution of this pathway to the functional outcomes, however, could be addressed by knockdown studies. In addition, since there is cross-communication between the MAPK and the autophagy pathway [230], further studies could be performed to address whether autophagy is induced alongside the activated p38–MAPK signaling network.

##### Immune System

*Simulated Microgravity using Cells:* In human U937 cells infected with enteropathogenic *E. coli* (EPEC) and subjected to RCCS-3DQ simulated microgravity conditions for 72 h, RNA sequencing identified 25 transcripts within the MAPK signaling pathway that were downregulated (i.e., MAPK3 and MAPK2K6), with 11 transcripts that were increased (i.e., DUSP) [233]. In addition, at the protein level, p-ERK1/2 and p-p38 were reduced, with no change in p-JNK and their total proteins [233].

*Real Microgravity using Cells:* Unstimulated human CD4+ T lymphocytes subjected to real microgravity aboard the MASER-12 mission rocket (~6 min microgravity) elicited reduced p-ERK1/2 activation in the absence of any change in global tyrosine phosphorylation levels [88].

*Simulated Microgravity using Rodent Models:* Macrophages isolated from male C57BL/6 mice were subjected to simulated microgravity with RCCS (and Cytodex 3 microcarrier supplementation) for 24 h [234]. Under these conditions, arginase I, a marker of immunosuppressive activity, was increased at the RNA and protein level with microgravity; further, C/EBPβ (a transcriptional regulator of arginase I) also increased [234]. While no detectable change in p-JNK and p-ERK was identified, p-p38 was increased, although no data for total proteins is shown [234]. While anisomycin and SP600125 combinatorial treatment (independently of microgravity) activated p38 as well as elevated C/EBPβ and arginase I expression, these alterations were reversed with SB202190, a p38 inhibitor; these findings suggest that targeting the p38 pathway could potentially oppose the changes induced by microgravity [234].

Male C57BL/6J mice subjected to hindlimb unloading for 28 days showed alterations in their physiology (i.e., bone loss, tissue abnormalities, reduced white blood cells (WBCs)) and bone marrow features including reduced hematopoietic stem cell apoptosis, elevated expression of the Ki67 proliferation marker, and reduced hematopoiesis markers (i.e., HOXA9, Bmi, and Yap1) [235]. Further, RNA sequencing uncovered enriched expression of MAPK signaling components [235]. However, addition of hypoxanthine, identified to be reduced via untargeted metabolomics under these microgravity conditions, could recover these MAPK signaling mediators as well as hematopoietic differentiation in the simulated microgravity-treated mice [235].

Across the above studies using immune cells, reduced p-p38 levels were identified, while cells isolated from rodent models show increased levels of p-p38 or increased expression of MAPK signaling components. Altogether, the contribution of MAPK to the reported cellular outcomes could be addressed using genetic manipulation strategies.

##### Cardiovascular System

*Simulated Microgravity using Cells:* Conditioned media collected from human endothelial progenitor cells that were subjected to simulated microgravity via Gravite for 24 h was applied to HUVEC cells (4 h to 5 days) [236]. Changes in HUVEC function included increased proliferation, elevated nitric oxide (NO) levels, elevated migration, and formation of tubes [236]. Further, p-ERK1/2 levels were elevated after 4 h of treatment; however, an MAPK inhibitor, PD98059, not only reduced the p-ERK1/2 levels but also reduced markers of angiogenesis (i.e., VEGF RNA) and proliferation (i.e., Ki67) [236]. In addition, the conditioned media from the progenitor cells improved tibial bone fracture regeneration in a Sprague Dawley rat model, with evidence of increased capillaries and angiogenic features [236]. These results suggest that MAPK contributes to the functional outcomes observed; further, factors present in the cell culture media may improve regenerative responses. The factors present in the conditioned media could also be defined in future studies.

*Simulated Microgravity using Rodent Models:* C57BL/6 male mice subjected to simulated microgravity via tail suspension for 14 or 28 days displayed reduced muscle mass along with increased calpain activity, reduced heart mass, heart function, and size of cardiomyocytes [237]. Further, at 14 days, increased p-ERK1/2 and p-p38 were noted, which reduced at 28 days (their total proteins were unchanged, however) in the mice hearts [237]. Calpain knockout mice (specifically in cardiomyocytes) did not display these heart tissue alterations [237]. In cardiomyocyte cultures, isolated from murine neonates, that were subjected to simulated microgravity using the RCCS-4DQ device, the cells displayed increased p-ERK1/2 at 6 h and sustained elevated p-p38 levels up to 24 h (no change in their total proteins); however, inhibition of calpain using calpain inhibitor-III hindered these changes [237]. In both studies (cells and rodent model), targeting calpain reverted the microgravity-induced effects on MAPK. These findings are relevant to skin health, as it has been reported that inhibition of calpain mediates protection against ultraviolet (UV) radiation-induced breakdown of proteins at the dermis–epidermis junction in skin tissue [238].

##### Nervous System

*Simulated Microgravity using Rodent Models:* Sprague Dawley male rats subjected to a stress composite of tail suspension, isolation rearing, steady-state noise, and circadian rhythm alterations for 6 weeks displayed behavioral changes (i.e., reduced pleasure and increased immobility) and hippocampal neuronal damage [239]. Further, the rat hippocampal tissue showed increased p-ERK1/2 and p-p38 levels in the absence of p-JNK alterations; in addition, reduced p-PI3K, p-AKT, and p-mTOR were noted, with no change in their total protein levels [239]. However, Baoyuan Jieyu Formula (BYJYF, a Chinese medicine) increased rat pleasure and immobility behaviors, improved hippocampal neuronal health, and opposed these signaling alterations [239]. Thus, this treatment may be of benefit to astronauts who venture on spaceflights. It is unknown whether this agent could improve the health of other tissues including skin.

##### Skeletal System

*Simulated Microgravity using Cells:* Murine MC3T3-E1 osteoblast-like cells elicited reduced growth when subjected to simulated microgravity (15 to 80 min) via clinorotation [240]. Aboard the TR-1A6 sounding rocket, these cells did not show a change in p-ERK protein levels following EGF stimulation, although c-fos RNA levels were reduced [240]. However, while no change in p-ERK levels was identified, the protein level of c-fos, a downstream mediator, is unclear.

While human NHOst normal osteoblast cells subjected to 3D clinostat up to 21 days did not show differences in cellular viability or formation of spheroids (i.e., bone nodules), the signaling mediator that was altered included reduced p-p38 in the absence of changes in p-ERK1/2, p-SAPK, or their total proteins across an array of time points (10, 30 min, 1, 3, 5 h, and 7, 14, 21 days) [241]. The contribution of the p38 pathway to the functional outcomes in these cells remains unclear.

##### Urinary System

*Simulated Microgravity using Rodent Models:* C57BL/6 mice subjected to tail suspension for 4 weeks displayed changes in kidney function [242]. These changes were noted after 1 week of simulated microgravity and included elevated serum copeptin (a precursor of vasopressin) levels and kidney aquaporin-2 (AQP2) protein with reduced p-AQP2 levels [242]. After 4 weeks of simulated microgravity, reduced levels of copeptin and AQP2 RNA/protein were observed [242]. These alterations were accompanied by elevated p-p38 levels (with no change in its total protein), which may contribute to the altered AQP2 expression [242]. Indeed, inhibition of p38 via SB203580 inhibitor restored AQP2 protein levels along with diminished p-AQP2 levels [242].

While AQP2 is not a main aquaporin expressed in skin tissues (instead AQP3 [243]), aquaporins support water and glycerol transport [243]. Indeed, knockout AQP3 mice show loss of skin barrier integrity along with reduced hydration of the stratum corneum [243]. Furthermore, altered expression of AQP3 has been reported in various skin dermatoses [243]. Thus, further studies may be beneficial to examine changes in AQP in skin tissue and the contribution of the MAPK pathway to AQP regulation.

##### Reproductive System

*Simulated Microgravity using Cells:* Human breast CRL-2351 cancer cells subjected to RPM for 5 days displayed formation of spheroids in the absence of changes in cellular viability [147]. Actin filaments were disorganized along with increased vimentin RNA and protein levels; further, the RNA and protein levels of MAPK1 were increased in both adherent and spheroids [147]. These findings suggest that the MAPK pathway may contribute to regulation of the cytoskeleton under conditions of stimulated microgravity.

*Real Microgravity using Cells:* Under real microgravity via parabolic flight, human breast cancer MDA-MB-231 cells displayed increased RNA expression of ERK1 (at P1) and ERK2 (at P31); however, JNK1 (MAPK8) RNA was reduced at P1 and unchanged at P31 while RAF1 RNA was unchanged [244]. It is unclear whether these RNA-level changes would result in protein-level alterations.

#### 4.3.6. PI3K–AKT

The PI3K–AKT signaling cascade is critical for maintenance of skin barrier function and promotes wound healing [245]. In various skin dermatoses such as psoriasis and atopic dermatitis, this pathway is dysregulated [245]. Thus, microgravity-induced changes in the PI3K–AKT signaling network were reviewed across various model systems.

##### Stem Cells

*Simulated Microgravity using Cells:* Bone marrow stem cells derived from male Sprague Dawley rats were subjected to simulated microgravity via the clinostat for 1 to 4 days [246]. Cellular changes included reduced proliferative capacity with increased percentage of cells in the G0/G1 phase of the cell cycle as well as diminished cellular response to growth factors (i.e., EGF, bFGF, and IGF-1) [246]. In addition, cytoskeletal alterations (i.e., reduced actin a1, ARP3, cofilin1) and signaling changes (i.e., RASA1, RAF1, RAB9, RAB10, RAB13) were noted at the RNA level; reduced p-AKT and p-ERK proteins were also uncovered, although no data for their total proteins was presented [246].

In another study, human Isl-1+ cardiac stem cells, isolated from the right atrium of neonates and adults, were subjected to 2D clinostat for 6 to 7 days followed by an assessment of functional outcomes [247]. While no change in migratory capacity was observed, proliferation of neonatal cells and telomerase reverse transcriptase (TERT) RNA expression were increased [247]. Further, although changes in p-ERK or p-AKT were absent, markers of stemness were increased (i.e., MESP1, OCT-4, Brachyury) along with increased differentiation (i.e., tube formation) in adult cells [247].

In human embryonic stem cells subjected to simulated microgravity with RPM for 4 and 7 days, elevated RNA expression of mediators involved in the PI3K/AKT signaling cascade were identified via RNA sequencing, which correlated with cellular differentiation to hematopoietic progenitors [248].

Human cardiovascular progenitors, isolated from neonatal atrial tissue, were subjected to 2D clinostat for 6 to 7 days and analyzed for changes in signaling mediators [249]. RNA-level alterations noted under microgravity conditions included decreased RhoA, Cdc42, and CTNNB1 along with elevated WNT5a, WNT9a, PKC, MAPK3, MAPK8, MAPK9, AKT1, AKT3, and PIK3CA [249].

*Real Microgravity using Cells:* Human cardiovascular progenitors were also exposed to real microgravity aboard the ISS for 12 days, which uncovered signaling pathway alterations including increased transcript levels of PKC, PIK3CA, GRB2, and RAF1; increased protein-level changes included p-PKCα and p-AKT [249].

It is unclear whether the RNA-level changes across the above studies would translate into similar changes at the protein level. Across these studies using diverse stem cells, the changes in p-AKT varied and could be due to the diverse range of stem cells and the differences in the simulated microgravity conditions.

##### Immune System

*Simulated Microgravity using Cells:* Human promyelocytic HL-60 leukemia cells subjected to simulated microgravity via RCCS (HARV) for 72 h displayed reduced cell numbers, which coincided with increased apoptosis, elevated DNA damage, and increased ROS levels [250]. Further, these cellular outcomes occurred with reduced p-AKT and p-ERK1/2 levels in the absence of change in total AKT and ERK proteins [250]. The diminished level of p-AKT correlates with increased apoptosis, per the role of PI3K–AKT as a mediator of cell survival signaling.

##### Cardiovascular System

*Simulated Microgravity using Cells:* In human endothelial HUVEC-C cells exposed to 2D clinostat for 24 h, increased migration and tube lengths with reduced cell surface caveolae were uncovered [251]. These changes were associated with increased p-AKT levels (no change in total AKT) as well as eNOS RNA/protein expression [251]. However, an eNOS inhibitor, L-NAME, opposed the microgravity-induced changes in migration and tube formation [251]. Further, LY294002, a PI3K inhibitor, reduced p-AKT, eNOS, and p-eNOS proteins under these conditions [251]. However, human pulmonary microvascular endothelial cells exposed to simulated microgravity with the clinostat for 72 h displayed reduced cell size, chromatin condensation, apoptotic features, and disrupted cell–cell junctions [252]. The cytoskeleton was disrupted (i.e., F-actin breakdown) and p-AKT as well as PI3K were reduced without change in total AKT [252]. The differences in outcomes could be due to the different source of endothelial cells and the exposure period to the microgravity.

*Real Microgravity using Cells:* Via RNA sequencing and transcriptional network analysis, human aortic smooth muscle cells exposed to real microgravity aboard the ISS for 3 days displayed upregulated STAT3 and downregulated PI3K/AKT signaling components, which were associated with reduced cell surface receptor expression (i.e., IL1R1, IL7R, IL13RA2, IL15RA, and ITGA2) [103]. Further studies could be performed to assess whether the changes in the PI3K/AKT network impact any functional change induced by the microgravity.

*Simulated Microgravity using Rodents:* In another study, male Sprague Dawley rats subjected to hindlimb unloading for up to 28 days displayed increased ER stress markers (i.e., CHOP, GRP78, ATF4, p-IRE1, p-eIF2α, and XBP1) and transition to a synthetic phenotype (i.e., reduced α-SMA, calponin, SM-MHC, and caldesmon) in cerebral vascular smooth muscle cells from arteries (VSMCs) [253]. In addition, signaling pathways were altered, including elevated protein levels of p-PI3K, p-AKT, p-mTOR; however, no data for their total proteins was shown [253]. Recovery from these simulated microgravity-induced changes occurred via the antioxidant MitoTEMPO or the ER stress inhibitor 4-PBA [253]. In the absence of total protein data for p-PI3K and p-AKT, it is challenging to assess whether there are increased phospho-levels.

##### Muscular System

*Simulated Microgravity using Rodent Models:* Female C57BL/6 mice subjected to hindlimb suspension for 5 days displayed reduced p-AKT (T308 and S473) protein levels in addition to reduced pTSC2 (T1462) and p70S6K (T389) levels in gastrocnemius muscles; their total proteins remained unchanged [254]. However, with irradiation, the protein levels of pAKT (T308 and S473) and p-TSC2 (T1462) were restored to control levels [254]. In an independent study, male C57BL/6J mice exposed to hindlimb unloading for 14 days displayed reduced muscle mass, reduced overall body weight, and reduced strength [255]. While AKT activation and downstream signaling mediators (i.e., p70S6K) were unchanged in the tibialis anterior muscles when subjected to hindlimb unloading, the physiological changes were partially restored with anti-myostatin antibody [255]. In the latter study, with the longer simulated microgravity exposure, adaptation may have occurred, leading to the absence of change in the AKT pathway.

Male Sprague Dawley rats subjected to hindlimb unloading for 28 days were characterized by elevated p-AKT (S473) protein levels in soleus muscles, which diminished following reloading (28 days); total AKT levels were, however, not assessed [256]. Male Sprague Dawley rats subjected to tail suspension for 30 days displayed reduced muscle weight and cross-sectional area of soleus muscle fibers [257]. Signaling responses were also altered under these conditions, including reduced PI3K and p-AKT proteins with elevated GRP78, CHOP, p-JNK, and caspase-12 protein [257]. However, these alterations were partially recovered with the Chinese medicinal agent Xuefuzhuyu (XFZY) [257]. Overall, there appears to be recovery with reloading and different outcomes depending on the length of stimulated microgravity exposure.

*Real Microgravity using Rodent Models:* When C57BL/6 female mice were subjected to real microgravity aboard the STS-135 mission for 13 days, they displayed reduced p-AKT protein levels in the tibialis anterior muscles, which also were characterized by decreased muscle mass; these outcomes contrasted with masticatory muscles, which did not display a change in muscle mass [258]. This study suggests that muscles from different sites may respond to microgravity in unique ways.

##### Respiratory System

*Simulated Microgravity using Cells:* When human lung cancer CRL-5889 cells were subjected to RPM for 72 h, the microgravity outcomes included increased roundedness and spheroid formation with apoptotic features and enrichment of F-actin at the cell surface [259]. However, these cellular outcomes were not accompanied by alterations in transcript levels of AKT3, PIK3CA, or NFE2L2 [259]. The protein levels were not assessed in this study, and, thus, the AKT changes, if any induced by the microgravity, remain unclear.

##### Digestive and Endocrine System

*Simulated Microgravity using Cells:* Human colorectal cancer cell lines (i.e., DLD1, HCT116, and SW620) were subjected to simulated microgravity conditions using RCCS (HARV) for 48 h [260]. Functional outcomes included reduced cellular viability with elevated apoptosis features [260]. With DLD1 cells, signaling pathways were altered, including reduced AKT and p-AKT (T308) proteins along with elevated PTEN protein [260]. With respect to the autophagy pathway, LC3-II and p62 proteins were elevated; further, RNA levels of various autophagic markers were also altered (i.e., ATG4B, ATG7, ATG16L1, Beclin1, and LC3B were increased, while ATG5 and ATG12 were reduced) [260]. The association of increased apoptosis with reduced p-AKT is as expected based on the role of PI3K-AKT as a cell survival network. While changes in the autophagy were also noted, further studies could address changes in ER stress.

When human pancreatic cancer cells (i.e., PaCa-44, AsPC-1, CFPAC-1) were subjected to simulated microgravity via RPM for up to 9 days, cellular outcomes included aggregate formation characterized by a necrotic core [261]. Under these simulated microgravity conditions, proteomic analysis uncovered cytoskeletal alterations (i.e., increased Cdc42, tubulin, and actin with reduced lamin A/B proteins) as well as increased PI3K/AKT and autophagy signaling components [261]. Further studies could address the connection between the PI3K–AKT and autophagy network to the necrotic core of the aggregates induced by microgravity exposure.

##### Nervous and Sensory System

*Simulated Microgravity using Cells:* When RCCS (HARV) was applied to human CHME3 microglial as well as glioblastoma U87MG and A172 cells for 72 h, cellular outcomes included increased spheroid formation with reduced viability associated with apoptotic features and DNA damage [262]. These changes coincided with increased p-ERK1/2 and p-AKT levels without alterations in their total proteins [262]. In murine HT22 hippocampal neurons exposed to RPM for 48 h, cell viability was also reduced with elevated ROS levels and reduced AKT protein; however, restoration of these outcomes to the levels of controls was attained using Trolox and/or r-Irisin treatments [263]. The outcomes to the microgravity exposure appear to be similar across the microglial, glioblastoma, and hippocampal neuronal cells. While total AKT protein was assessed, the activation status remains unclear.

*Simulated Microgravity using Rodents:* Male Sprague Dawley rats subjected to tail suspension for 8 weeks displayed reduced retinal thickness of the outer nuclear layer, reduced inner and outer segment layers of the photoreceptors, as well as reduced cone and rod nuclei numbers [264]. These changes were associated with reduced p-PI3K and p-AKT levels, with no change in their total proteins [264]. Hydrogen-rich water (HRW) not only improved eye health of the rats but also restored the PI3K/AKT signaling cascade [264].

*Simulated Microgravity using Rodents:* ICR male mice subjected to hindlimb suspension for 28 days displayed diminished memory capacity and swimming distance as well as increased oxidative stress features (i.e., MDA and NO levels) [265]. These alterations were associated with reduced p-AKT in the absence of changes in the total AKT level [265]. However, the Chinese medicinal agent Gastrodia Elata Blume (GEB) restored all these features to the levels of controls [265].

In an independent study, ICR mice subjected to hindlimb unloading for 28 days demonstrated reduced weight and cognitive functions along with neuronal damage [266]. These physiological alterations were accompanied by reduced superoxide dismutase (SOD) protein expression along with increased GSSG, MDA, and inflammatory cytokines in the hippocampus (i.e., TNF-α, IL-6, and IFN-γ) [266]. Further, reduced protein levels of total AKT, PI3K, and mTOR were uncovered under simulated microgravity [266]. Restoration of these alterations to those of controls could also be attained with Gastrodia Elata Blume (GEB) [266].

Male Sprague Dawley rats subjected to tail suspension up to 28 days showed increased TUNEL-positive apoptotic cells in the posterior parietal complex (PPC) and lateral geniculate nucleus (LGN) brain regions at 7 days, with recovery at 14 and 28 days [267]. From a signaling perspective, in the LGN region, reduced p-PI3K and p-AKT were noted at 7 days, but these reductions were restored to control levels at 14 and 28 days [267].

Male Sprague Dawley rats exposed to tail suspension for 4 weeks elicited reduced cognitive function in the absence of reduced body weight [268]. Further, along with neuronal apoptosis features and ER stress, reduced p-PI3K and p-AKT (S473) levels with increased p-p38 protein levels were observed in the absence of change in their total proteins [268]. However, berberine could improve cognition and recover changes in the AKT signaling cascade [268].

Wistar male rats exposed to hindlimb unloading for 4 weeks were characterized by altered behavior (i.e., increased time to reach platform in the Morris Water Maze), reduced superoxide dismutase (SOD) and glutathione peroxidase activities, along with increased MDA, increased hydrogen peroxide, and reduced p-AKT protein expression (total AKT remained unchanged) in the cortex [269]. Ginsenosides, namely Rb1 and Rg1, opposed the behavioral outcome as well as changes in reactive oxygen species (ROS) and p-AKT levels [269].

Across the above studies, restoration of responses could be attained with various compounds (GEB, berberine, Trolox, r-Irisin, HRW, and Ginsenosides) or with longer periods of microgravity exposure, which may indicate an adaptation response to the microgravity.

##### Skeletal System

*Simulated Microgravity using Cells:* When murine MC3T3-E1 osteoblast-like cells were subjected to simulated microgravity via the HARV device for 5 days, they displayed loss of osteogenic phenotype that was characterized by the loss of RUNX2, OCN, and COL I RNA expression [270]. These changes coincided with increased apoptotic features, reduced p-AKT, and reduced total AKT proteins [270].

Calvarial osteoblasts isolated from newborn Sprague Dawley rats subjected to simulated microgravity with the 2D clinostat for 3 days also elicited loss of osteogenic features including reduced RNA levels of RUNX2, OPN, and ALP as well as reduced ALP activity and protein [271]. However, while frizzled 9 (Fzd9) was reduced at the RNA and protein level under these microgravity conditions, its overexpression restored the osteogenic phenotype [271]. Other simulated microgravity alterations in the calvarial osteoblasts included cytoskeletal alterations (i.e., reduced F-actin and increased G-actin coinciding with reduced paxillin); these alterations could be recovered by Fzd9 overexpression [271]. In terms of signaling alterations, nuclear movement of β-catenin was reduced and p-GSK3 levels were increased [271]. Other signaling pathway changes included reduced YAP nuclear translocation as well as reduced p-ERK and p-AKT levels (total levels, however, were not assessed) [271]. The YAP localization and ERK/AKT signaling could be recovered by Fzd9 overexpression [271]. Jasplakinolide, a stabilizer of actin, could also recover YAP localization and actin organization [271].

*Simulated Microgravity using Rodents:* Male C57BL/6 mice exposed to a combination of tail suspension with irradiation for 14 days elicited reduced bone mineral density (BMD) as well as increased senescence and reactive oxygen species (ROS) in their bone marrow-derived stem cells [272]. Further, RNA sequencing uncovered elevated PI3K/AKT signaling components with increased p-AKT and p-FOXO3 proteins as well as reduced FOXO3 nuclear localization [272]. However, the Chinese medicine cordycepin, extracted from *Cordyceps sinensis*, hindered these changes by improving bone health and opposing the PI3K/AKT signaling outcomes [272].

*Human Participants:* When healthy human subjects were subjected to dry immersion, a form of simulated microgravity, for 5 days, they displayed reduced body weight and oxygen uptake along with elevated heart rate and decreased knee extension [178]. Biopsy of a skeletal muscle showed reduced protein expression of signaling mediators (i.e., p-4EBP1, p-RPS6, and total RPS6) and reduced RNA expression of autophagic mediators (i.e., ATG5, GABARAPL1, and BNIP3) [178].

In some studies, RNA-level changes in AKT were only measured rather than protein expression. However, across the above studies, the changes in the p-AKT pathway correlated with the functional outcome (i.e., reduced viability). Additional countermeasures to simulated microgravity that were effective were identified, including cordycepin and jasplakinolide.

##### Reproductive System

*Simulated Microgravity using Cells:* Human MDA-MB-231 breast cancer cells exposed to simulated microgravity via the RPM device for 24 and 72 h displayed increased cell roundedness and the generation of multicellular spheroids (MCSs) [273]. Further, cellular viability was reduced and was associated with apoptotic features [273]. The actin network was disorganized, and vimentin became aggregated as well as perinuclear in its localization in the MCS fraction; in addition, reduced p-AKT and p-ERK levels with no change in total AKT and ERK proteins were observed [273]. Human MCF-7 breast cancer cells subjected to RPM for 24 and 72 h also led to the generation of spheroids characterized by cell death features [274]. Cytoskeletal alterations included a fragmented F-actin network that was enriched at the perinuclear region, disorganized tubulin that was aggregated at the nuclear region, and reduced vinculin expression [274]. However, these changes were instead associated with increased p-AKT and reduced p-ERK levels when normalized to their respective total protein levels [274]. The varied response in p-AKT to the simulated microgravity across these two studies may be due to the different cell types utilized. This could be confirmed by analyzing the response of multiple breast cancer cell lines to simulated microgravity exposure concurrently.

Human endometrial stromal cells exposed to simulated microgravity via the 3D clinostat up to 36 h displayed reduced cell growth in the absence of elevated death and cell migration [275]. These cellular outcomes were accompanied by reduced p-AKT (T308 and S473, in the absence of change in total AKT), reduced p-FOXO3a (and total FOXO3a), and reduced autophagy markers (i.e., p62, LC3B-II, beclin1, and UVRAG), with no change in p-S6K1 or p-4EBP1 proteins [275]. Altogether, as expected, reduced viability is associated with reduced p-AKT in these cells.

#### 4.3.7. PKC

Protein Kinase C (PKC) comprises a family of members which contribute to cellular growth and differentiation across a wide array of cell types including those in skin, as well as a role in melanogenesis in melanocytes [276]. PKCs have recently been shown to be associated with desmosomal components and to elicit a function as a structural regulator [277]. Thus, microgravity-induced changes in PKC signaling were reviewed across various model systems.

##### Immune System

*Simulated Microgravity using Cells:* Human peripheral blood monocytic cells (PBMCs) were subjected to simulated microgravity via the RWV device for 3 days under phytohemagglutinin (PHA) stimulation [278]. Under these conditions, these cells elicited reduced proliferative capacity associated with reduced generation of lymphokines with elevated monokines [278]. However, when treated with phorbol myristate acetate (PMA, a PKC activator), the proliferative capacity could be recovered [278]. In an independent study, PBMCs subjected to clinostat for 48 h displayed decreased S phase population under PHA or Leu4 stimulation [279]. Further, while reduced cell surface expression of CD25 was noted at 24 h, recovery of its expression by PMA stimulation occurred in the T cell population [279]. In another study, PBMCs subjected to RWV up to 72 h displayed reduced cell movement [280]. However, PMA treatment was able to restore the migratory capacity [280]. Furthermore, reduced expression of calcium-independent PKC isoforms (i.e., PKC-δ and PKC-ε) at the RNA and protein levels were noted in PBMCs following simulated microgravity with RWV up to 96 h [281].

Wistar rat pulmonary lymphocytes subjected to RWV (HARV) for 72 h elicited reduced proliferative capacity, reduced cell surface expression of CD25 and CD71, and reduced PKC activity as well as p-ERK1/2 proteins in response to concanavalin A (Con A) or Con A/leptin activation [282]. PMA stimulation could recover these responses to some degree [282].

*Real Microgravity using Cells:* Under real microgravity aboard the Airbus A300 ZERO-G during parabolic flight, there was no change in platelet shape, cytoplasmic granules, or phosphorylation of a 40K band with PMA treatment [283]. Further, there was no change in human pro-monocytic U937 cell growth, translocation of PKC, or in protein expression of PKC-δ and PKC-βII in response to phorbol ester aboard a 9-day real microgravity STS-76 mission [284]. However, aboard the STS-81 mission for 10 days, human U937 cells showed reduced movement of PKC-δ protein to the membrane fraction from the cytosol upon phorbol ester treatment [285].

Across these studies using immune cells, additional research could be performed to examine how simulated microgravity alters the activity as well as localization of the various members of the PKC family (i.e., conventional, atypical, and novel).

##### Respiratory System

*Simulated Microgravity using Cells:* Tracheal epithelial explant cultures derived from rabbits were subjected to simulated microgravity using HARV between 3 and 6 days [286]. While there was no change in cellular viability or calcium waves, TPA (a phorbol ester) stimulation reduced the calcium oscillations in the air–liquid interface and sheet cultures, supporting a role for PKC under simulated microgravity [286]. While no direct evidence was shown for PKC, the PKC isoform involved in this process could be investigated.

##### Endocrine System

*Simulated Microgravity using Cells:* When cultured under simulated microgravity using RPM for 7 and 14 days, human thyroid normal Nthy-ori 3-1 and cancer FTC-133 cells developed into multicellular spheroids (MCSs) as well as adherent monolayers (ADs) [287]. Not only did both cell lines display disorganized F-actin structures in MCS cultures, but the RNA levels of beta-actin and tubulin beta (TUBB) were elevated in both AD and MCS structures at 14 days [287]. Further, the RNA levels of signaling molecules (i.e., PKC, AKT, and ERK1/2) were significantly elevated in the normal Nthy-ori 3-1 cells under simulated microgravity [287]. While RNA-level changes were noted in PKC, it is unclear whether these translate into protein-level changes.

#### 4.3.8. Mixed Pathways

A subset of articles investigated changes across multiple signaling pathways in response to microgravity exposure. These articles were reviewed, and key elements are discussed below.

##### Stem Cells

*Simulated Microgravity using Cells:* Human mesenchymal stem cells subjected to simulated microgravity via the RCCS (HARV) device for 7 days displayed reduced osteoblastic cell features, including diminished RNA levels of RUNX2, ALP, and Col 1α2, along with increased protein levels of integrin α2 and β1 [288]. Further, signaling mediators were reduced at the protein level, including p-FAK, p-PYK2, activated Ras, and p-ERK in the absence of changes in p-AKT as well as total ERK and AKT proteins [288].

*Real Microgravity using Cells:* Aboard the SJ-10 satellite, the mesenchymal stem cells subjected to real microgravity for 2 days also showed reduced osteoblast features, including reduced RNA levels of ALP, RUNX2, and BMP2 [289]. Under these conditions, reduced p-FAK along with increased p-AKT and p-p38 were noted at the protein level in the absence of change in total AKT or p38 [289].

##### Immune System

*Simulated Microgravity using Cells:* When human T cells isolated from leukocytes were subjected to simulated microgravity via the RPM device, p-CREB levels were reduced along with unchanged levels of p-PI3K and p-PKC levels when stimulated with concanavalin A (Con A) and CD28 antibody for 30 min [290]. Using human HDLM-2 Hodgkin’s lymphoma cells subjected to 3D clinostat for 2 days, elevated RNA levels of autophagy markers (i.e., LC3, BECN1, ULK1, ATG14) were observed [291]. Further, protein levels of p-ULK1, ATF4, BECN1, and lipidated LC3 as well as signaling pathway mediators such as p-PTEN, p-ERK, p-JNK were increased with reduced p-mTOR, p-AKT (S473), p-p70S6K (T389) levels and no change in their total protein levels [291].

##### Cardiovascular System

*Real Microgravity using Rodents:* Female C57BL/6J mice subjected to real microgravity aboard the ISS for 30 days showed increased RNA expression of signaling mediators in heart tissue, including those within the ERK–MAPK, PI3K–AKT, GPCR–cAMP pathways, as determined by RNA sequencing [292].

##### Nervous System

*Real Microgravity using Rodent Models:* Male Sprague Dawley rats were subjected to simulated spaceflight conditions (SLSE), which included tail suspension, isolation rearing, steady-state noise, and altered light–dark cycles for 48 days [293]. SLSE negatively impacted the rodent’s memory and cognitive abilities [293]. Specifically, neurotransmitter levels such as 5-HT, Ach, and NE were reduced in the cortex and hippocampus along with reduced neuronal numbers [293]. Signaling mediators were also affected by SLSE in these tissues, including reduced levels of p-PI3K, p-AKT, p-CREB, and p-mTOR levels [293]. In the hippocampus, PI3K, AKT, p-JNK, p-p38, and p38 were elevated [293]. However, a compound extracted from *P. ginseng*, namely dammarane sapogenins (DS), improved the memory and cognitive function of the rats subjected to SLSE; further, the compound restored neurotransmitter levels, neuronal numbers, and signaling events in hippocampi and cortices [293].

##### Skeletal System

*Real Microgravity using Cells:* Rat osteoblasts isolated from femurs were also subjected to real microgravity on a space shuttle for up to 5 days and were noted to express elevated RNA levels of signaling mediators including FAK, PLC-γ1/2, SOS, Rho-GAP, Raf-A, ERK-2, PLC-β1/3/4, G-β, and G-α1 [294].

##### Reproductive System

*Simulated Microgravity using Cells:* Human breast cancer cell lines (MCF-7 and MDA-MB-231) subjected to RPM for 14 days displayed increased multicellular spheroid (MCS) formation [295]. In MCF-7 cells, MAPK14 was elevated at both the RNA and protein levels, including p-MAPK14 protein levels [295]. In MDA-MB-231 cells, elevated p-ERK1/2 and total ERK1/2 protein were noted along with reduced p-MAPK14 protein with no change in total MAPK14 [295].

When human PC3 prostate cancer cells were subjected to simulated microgravity via the RPM device for up to 5 days, functional alterations included elevated multicellular spheroid (MCS) formation, which lacked apoptotic or necrotic features [296]. However, signaling pathway alterations were uncovered, including increased AKT, mTOR, ERK1, and ERK2 RNAs with no change in RICTOR and MAPK2K1 transcripts [296].

## 5. Relevant Findings from the Extended Literature Coverage

As discussed in Section 2, the findings presented in Section 3 favors Western European and American research groups. As such, secondary PubMed searches were performed to expand the comprehensiveness of this review.

### 5.1. Cytoskeleton

#### 5.1.1. Integumentary System

*Real Microgravity using Rodents:* Mast cells are present in skin tissue and contribute to its remodeling. It is well established that mast cells contribute to the remodeling process of the extracellular matrix (ECM) and are also involved in alterations in the fibrotic tissue defined as fibrillogenesis [297]. A real microgravity study, via the Rodent Research-4 mission aboard the ISS, was conducted using C57BL/6J mice (9–12 weeks old) [297]. Skin sections were analyzed via immunohistochemical staining [297]. Dermis isolated from control mice was characterized by the proximity of mast cells to fibroblasts and dominance of type I collagen in fiber bundles with some type III collagen at its edges [297]. Dermis from mice subjected to spaceflight presented changes in the skin microenvironment [297]. This included elevated levels of type III collagen, along with increased mast cell area, mast cell secretion with small-sized granules within the extracellular matrix, and reduced mast cell proximity to fibroblasts [297]. To uncover the mechanisms underlying these changes, further work is needed, including defining the contribution of matrix metalloproteinases (MMPs) and signaling events.

#### 5.1.2. Cardiovascular System

*Simulated Microgravity using Cells:* To broaden our understanding of how functional changes (e.g., migration) are associated with cytoskeletal alterations, simulated microgravity using clinorotation (1 h to 3 days) using primary human umbilical vein endothelial cells (ECs) was performed [298]. Wound healing occurred under microgravity (1–4 h) but was absent in controls; this finding was associated with altered actin filament structure (thinning) and organization (re-localization to cell edges) noted up to 24 h post-microgravity simulation [298]. While visual changes were noted in the cytoskeleton, further effort is needed to uncover the changes in signaling events that lead to the increased migratory capacity.

In an independent study, TNF-α-activated primary human umbilical vein endothelial cells were subjected to simulated microgravity using the RPM device (6 to 24 h) [299]. Compared to control cells (increased actin with stress bundle formation with filopodia at cell–cell contacts), microgravity caused reduced cellular flattening and filopodia, which coincided with increased actin fibers at the cell–cell regions [299]. These observations were consistent with VE-cadherin staining patterns, which were reduced with microgravity [299], thus supporting the altered connections between the cytoskeleton to cell–cell contact regions.

*Simulated Microgravity using Rodents:* When Wistar rats were subjected to antiorthostatic suspension (1 to 14 days), transversal stiffness of the left ventricular myocytes increased [300]. However, this returned to control levels with reloading (3, 7, and 14 days) [300]. Examination of key cytoskeletal components in the cardiomyocytes uncovered increased protein levels of DES (desmin), ACTN4 (actinin-4, in cytoplasm), and ACTG (gamma-actin, in membranes), with reduced levels of ACTN1 (actinin-1, in cytoplasm) [300]. These changes returned to control levels upon reloading [300].

*Human Participants:* Simulated microgravity can also cause muscular atrophy, such as head-down bed rest (HDBR). To further understand cardiovascular alterations, a HDBR study was conducted for a length of 21 days using healthy males (24–41 years of age) followed by venous blood collection for proteomic analyses of plasma proteins [301]. The LC-MS/MS analysis uncovered 25 significantly altered proteins, of which 17 were elevated (involved in fibrinolysis, blood coagulation, platelet degranulation) and 8 were decreased (involved in remodeling of the cytoskeleton amongst some other pathways) [301]. In an independent HDBR study for a length of 19 days, proteomic analysis of blood via LC-MS/MS was performed after a passive tilt test [302]. This experimental approach uncovered 17 significantly altered proteins that were elevated, including ACTA1 in addition to other focal adhesion proteins, with 6 proteins that were reduced (involved in transport and complement components) [302]. It is unclear how these changes relate to the broader physiological alterations observed in response to spaceflight.

#### 5.1.3. Pulmonary System

*Human Participants:* The collection and storage of blood and urine is challenging to perform on spaceflight missions. The collection of exhaled breath condensate (EBC) may be a biological material that is more feasible to collect and store [303]. A study was conducted to analyze the EBC from astronauts aboard a 169–199-day spaceflight mission; samples were collected 1-month prior to flight, upon landing, and 7 days post-landing [303]. These specimens were then subjected to LC-MS/MS proteomic profiling [303]. Across these groups, 164 proteins were detected, with several as part of the cytoskeleton (i.e., intermediate filaments including keratin) [303]. Further work is needed in this area to determine how changes in EBC reflect broader physiological alterations or, specifically, how they relate to changes in skin health due to the detection of keratins.

#### 5.1.4. Muscular System

*Simulated Microgravity using Rodent Models:* Muscle atrophy is observed in those subjected to spaceflight missions. Although there are noted changes in muscle structure, the underlying mechanisms that lead to these outcomes remain unclear. Towards this goal, Wistar rats subjected to antiorthostatic suspension (6 h) to assess stiffness of soleus muscle fibers and assess changes in gene expression at the RNA and protein level [304]. While there was no alteration in transversal stiffness with no change in RNA levels of ACTB, ACTG, TUBB2B, ARPC3, TMOD1, SVIL, and LCP1, reduced RNA levels of ACTN1 and ACTN4 were measured in the soleus fibers [304]. At the protein level, ACTN4 was increased (cytoplasm) with suspension compared to controls [304]. The discordance between the RNA and protein level may be due to regulatory mechanisms such as ubiquitination.

*Real Microgravity using Rodent Models:* Murine studies (C57BL/6) were conducted aboard the BION-M1 biosatellite (30-day mission) to analyze the stiffness of cardiomyocytes and muscle fibers (i.e., soleus and tibialis anterior) as well as assess changes to cytoskeletal elements [305]. While there were no changes in transversal stiffness in the cortical cytoskeletal in cardiomyocytes and soleus muscle fibers, there were reduced RNA and protein levels for ACTB (cytoplasm) and ACTN4 (membrane) of cardiomyocytes, with reduced RNA and protein for ACTB (cytoplasm) and ACTN1 (membrane) in soleus muscle fibers [305]. However, in the tibialis anterior muscle fibers, the stiffness of the cortical cytoskeleton was increased along with elevated protein levels of ACTB (membrane, although RNA levels were reduced) with reduced levels of ACTG [305]. These findings suggest that the different muscle sites may respond differently to spaceflight environment, and, furthermore, the changes in protein are not consistent with RNA-level changes, implicating regulation at each of these levels of expression. Further studies are needed to determine how changes in the muscle layer in skin fascia impact skin health.

C57BL/6N male mice were subjected to real microgravity aboard the BION-M1 biosatellite for 30 days to investigate alterations in thick and thin filament proteins in their heart and skeletal muscles [306]. While the total weights of the mice were unchanged, the gastrocnemius and tibialis muscles were reduced in weight under microgravity conditions [306]. While there was no change in myosin heavy chain (MyHC) in cardiac muscles, the slow form (IIA) was reduced with increased fast form (IIB) in the tibialis muscles [306]. The expression levels of giant sarcomeric cytoskeletal proteins, namely titin and nebulin, were also assessed [306]. While titin RNA increased in both heart and skeletal muscles, the protein level was unchanged in the cardiac muscle but reduced in the tibialis anterior muscle [306]. While nebulin also increased in the tibialis muscle at the RNA level, it was reduced at the protein level [306]. It is suggested that the ubiquitination pathway may be involved in the discordance between the RNA and protein levels of these cytoskeletal proteins in these muscle tissues [306].

In another study using C57BL/6 mice subjected to microgravity on the BION-M1 biosatellite mission (30-days), DES (desmin, an intermediate filament) was analyzed in ventricular cardiomyocytes and skeletal muscles [307]. While there was no difference in the stiffness of the cardiomyocytes or soleus fibers, increased stiffness was noted for tibialis anterior muscles with microgravity [307]. Further, desmin content remained unchanged, when measurable [307]. It is suggested that tissue readaptation to the microgravity environment occurred during the analysis period [307].

#### 5.1.5. Digestive System

*Simulated and Real Microgravity using Rodent Models:* Data collected from male C57BL/6N mice (19–20 weeks old) aboard the BION-M1 biosatellite mission (30-day) was compared to mice subjected to simulated microgravity (antiorthostatic suspension) and to Mongolian gerbils (4–4.5 months) on the Foton-M3 mission (12-days) to study the effect of microgravity on the stomach tissues [308]. Spaceflight on the BION-M1 mission uncovered changes in the gastric gland architecture, with shedding of epitheliocytes in addition to changes in the arrangement of the smooth muscle cells [308]. Further, there was increased staining (though weak) for alpha-smooth muscle actin in the stomach [308]. On the Foton-M3 mission, necrosis was visible along with inflammation and increased connective tissue in the stroma [308]. There were also regions devoid of epithelium in the glands with similar alpha-smooth muscle actin staining to BION-M1 in the stomach tissue [308]. In contrast, there were no morphological abnormalities (and changes in alpha-smooth muscle actin in the stomach) noted under the simulated microgravity study apart from increased flattening of the epithelium [308]. Altogether, spaceflight induces changes that differ to those of simulated microgravity. Further effort is needed to uncover the molecular mechanisms that underlie these structural alterations.

#### 5.1.6. Nervous System

*Real Microgravity using Rodent Models:* The thoracic segment of the spinal cord (T3–T5) from C57BL/6 male mice aboard the BION-M1 biosatellite mission (30-day) were analyzed for morphological changes in neurons of the motor system [309]. The spaceflight was associated with different functional features of the motor neurons [309]. This included larger neurons per area with fewer positively stained neurons for neurofilament (NF) and choline acetyltransferase (ChAT), which coincided with increased neurons positive for calbindin (CAB), neuronal nitric oxide synthase (nNOS), and caspase-3 [309]. Further effort is needed to investigate the impact of these changes on function.

#### 5.1.7. Reproductive System

*Real Microgravity using Rodent Models:* As described in an earlier section, simulated microgravity alters reproductive outcomes. To examine the mechanisms leading to the reduced reproductive success outcomes due to microgravity, RNA and protein coding for cytoskeletal components as well as DNA methylation patterns were assessed in testes and the duct deferens in mice subjected to spaceflight [310]. On the Rodent research-4 (RR-4) mission, C57BL/6J mice (9–12 weeks old) were subjected to 21–24 days of microgravity aboard the animal habitat section on the ISS [310]. No changes were noted in ACTB, ATCG, ACTN1, ACTN4, TUBB2B, and DES at the protein levels in testes and duct deferens [310]. However, the RNA levels for TUBB2B and ACTN1 were reduced compared to controls [310]. Further, genes involved in spermatogenesis were assessed (KDM5B, PRM1, and SPACA3), which did not change at the protein level [310]. At the RNA level, only KDM5B was elevated with spaceflight [310]. To address epigenetic alterations, the RNA and protein expression of enzymes that regulate the methylation process were assessed [310]. While there was no change detected in DNMT1, DNMT3A, HDAC1, or HAT1 at the protein level, the RNA was diminished for DNMT1 (testes) and HDAC1 (testes and deferens), with increased levels of TET2 (testes and deferens) and HAT1 (testes) [310]. Although there were few changes noted over the 3-week timeframe, this may reflect adaptation, but further research in this area would be beneficial [310].

*Simulated Microgravity using Rodent Models:* Since oocytes serve as the foundation for embryo development, the cytoskeletal components in ovarian tissues were examined in BALB/c female mice subjected to hindlimb suspension (96 h) as a form of simulated microgravity [311]. While there were reduced RNAs for ACTN4, no changes were detected in ACTB, ACTG, ACTN1, and ACTN4 proteins [311]. However, alpha-tubulin and the acetylated form of alpha-tubulin proteins were elevated [311]. While there were minimal changes to the cytoskeletal gene expression profile, the cellular respiration (i.e., OXPHOS) was markedly elevated, which coincided with increased RNA expression of mitochondrial respiratory chain components in short-term microgravity conditions [311].

In an independent study, oocyte–cumulus complexes were analyzed from BALB/c female mice subjected to simulated microgravity with the Gravite device (6 h) [312]. Along with reduced numbers of 3-cell stage embryos, reduced protein and altered localization (periphery to cell center) of acetylated alpha-tubulin were noted [312]. No protein changes were noted in F-, B-, or G-actin, although B-actin changed from a submembrane location to the cell center [312]. At the mRNA level, B-actin, G-actin, ARP2, SVIL, ACTN1, ACTN4, alpha-tubulin, and beta-tubulin were elevated, while gamma-tubulin was reduced [312]. LCP1, lamin B2, and vimentin RNAs were unaltered [312]. Altogether, the changes in the oocyte cytoskeleton may contribute to an altered cleavage process under microgravity.

### 5.2. Protein Trafficking

#### 5.2.1. Immune System

*Simulated Microgravity using Rodents:* It is well established that the cytoskeleton, nucleoskeleton, as well as ECM are critical sensors of gravity [313]. They work collaboratively to transmit signals into the nucleus to regulate gene expression [313]. Mechanoregulatory pathways such as Hippo signaling involve a subset of mediators that can be associated with the cytoskeleton and are located at cell–cell contacts [314]. One Hippo pathway mediator, YAP, is defined as a mechanosensor and works with the TAZ coactivator to regulate the commitment of mesenchymal stem cells to osteogenesis via RUNX2 [314]. A recent study demonstrates that YAP trafficking was altered under simulated microgravity conditions using BalbC male mice (6–8 weeks fold) and the Gravite device [315]. Specifically, bone marrow microexplants derived from the murine femur and tibia bones elicited reduced osteogenic potential and proliferative capacity under these conditions [315]. Furthermore, the gene expression of Hippo pathway components was reduced (i.e., YAP and TAZ), and translocation of YAP as well as RUNX2 into the nucleus was diminished [315]. From a skin perspective, YAP levels are reduced in human AD skin specimens and correlated with loss of skin barrier integrity [316]. Thus, the above findings linked to microgravity may occur in the skin tissue of astronauts subjected to spaceflight and may relate specifically to keratinocyte differentiation [317].

#### 5.2.2. Nervous System

*Real Microgravity using Rodent Models:* Molecules that contribute to transport and organellar movement are also altered in response to the effects of microgravity. Specifically, synataxin 1A (STX1A, involved in formation of vesicles, endocytosis, and exocytosis events), dynamin 3 (DNM3, regulates endocytosis and recycling events), myosin VA (MYO VA, involved in organellar transport), dynein light chain LC8-type 2 (DLC2, contributes to mitochondrial transport on neuronal axons), and vacuolar protein sorting ortholog 35 (VPS35, involved in cargo recognition for retrograde transport) were increased in both white and gray matter of brains from C57BL/6 female mice aboard the STS-135 mission (13 days) [318]. While these findings specifically relate to brain function, it is unknown whether the expression of these molecules may also be altered in skin tissues subjected to microgravity.

### 5.3. Signaling

#### 5.3.1. Stem Cells

*Simulated Microgravity using Cells:* During spaceflight, bone homeostasis is altered and is maintained by mesenchymal stem cells (MSCs). MSCs are multipotent and can differentiate into different stromal cells types (e.g., adipocytes, myoblasts, osteoblasts, amongst others) [319]. As discussed above, mechanotransduction supports bone homeostasis, including osteogenesis; however, the mechanisms that are altered with microgravity remain unclear. Thus, MSCs from adipose tissue were subjected to simulated microgravity using the Gravite device for 14 days [320]. An OMICs type of approach using RT^2^-profiler arrays uncovered reduced expression of Wnt signaling pathway components using MSCs [320]. While co-culturing MSCs with hematopoietic stem and progenitor cells (HSPCs) increased expression of Wnt signaling components, these changes were opposed by the effects of microgravity [320]. Thus, the presence of HSPCs may hinder the microgravity-induced effects on the MSC population. Whether similar transcript outcomes or proteomic changes in Wnt signaling may be observed for MSCs from skin tissue under the influence of microgravity is unknown.

#### 5.3.2. Immune System

*Real Microgravity using Rodent Models:* Although altered immune responses are well-established outcomes of spaceflight, the underlying mechanisms that result in these changes remain unclear. Towards this goal, the BION-M1 30-day mission, which housed C57BL/6 male mice (4–5 months of age), served to collect and analyze murine thymus lymphocytes to investigate changes in signaling [321]. In the lymphocytes from spleen, activation of NFκB was increased, while p-SAPK/JNK was reduced along with elevated levels of TLR4 (12 h post-landing) [321]. In the lymphocytes from thymus, p-IRF3 and p-SAPK/JNK were elevated (7 days post-landing), coinciding with increased apoptosis [321]. Cytokines were additionally measured in the blood, and IFN-γ and IL-6 were reduced [321]. Since several skin dermatoses are characterized by inflammation, altered cytokine production and deregulated signaling events may also occur in skin tissue in response to microgravity.

*Human Participants:* With respect to the p53 signaling network (defined as “guardian of the genome” [322]) and identifying changes that may arise under microgravity, a dry-immersion simulated microgravity (DI-SMG, 28 days) study was performed using CD3+ T cells isolated from blood collected from healthy males [323]. RNA was extracted for an OMICS RNA-sequencing approach to examine global changes in gene expression [323]. While p53 was elevated in most of the subjects, network analyses uncovered changes in regulators of apoptosis and senescence, cell cycle, DNA damage, as well as insulin signaling [323]. While there are reports of increased p53 expression in skin subjected to spaceflight [324,325,326,327], it remains unclear how changes in the p53 signaling network may influence the health of skin tissues under these conditions [322].

#### 5.3.3. Cardiovascular System

*Simulated Microgravity using Cells:* While spaceflight is known to exert detrimental effects on the cardiovascular system, the global changes at the transcriptome level remain unclear. Thus, primary human endothelial cells (HUVECs) were subjected to simulated microgravity using RPM for 24 h [328]. Signaling-focused RNA-level changes included those in JAK–STAT, EGF, VEGF, and WNT pathways amongst several others [328]. Since proteins are effectors of function, a global proteomic analysis would be beneficial to validate changes in these pathways at the protein level as well as assess their activation status, particularly in the context of skin tissues.

#### 5.3.4. Muscular System

*Simulated Microgravity using Rodent Models:* It is well established that spaceflight leads to deterioration of muscle function; however, the signaling pathways that lead to this outcome remain unclear. In an animal model using Wistar male rats (1.5–2 months of age) subjected to hindlimb suspension (up to 7 days), reduced p-GSK3β, p-ACC, and p-4EBP (normalized to their total proteins) levels were noted in the soleus muscles compared to controls [329]. While specific signaling molecules were examined, a more comprehensive proteomic analysis may be beneficial to understand the global scope of proteomic alterations under these simulated microgravity conditions. Since the fascia of the skin tissue contains muscles, it is unclear how microgravity-induced changes in this skin muscle layer at various sites affect overall skin health [330].

## 6. OMICS and Skin

A large array of OMICS datasets is available through the NASA GeneLab repository, which is publicly available to researchers [331,332,333]. The goal of this platform is to link scientists together to formulate testable hypotheses for future work. In this repository, the types of factors that were investigated include altered gravity levels, ionizing radiation, changes in pressure, and circadian rhythm alterations [332]. At the current time, the number of datasets available on NASA’s open science data repository is now over 500 [334].

The NASA GeneLab repository was reviewed for skin-relevant datasets; these are presented in Table 4 (accessed 27 July 2026).

The majority of these are transcription profiling studies using animal models (e.g., rats and mice) but also included human specimens. The data can be accessed at the following link: https://osdr.nasa.gov/bio/repo/search?q=skin&data_source=cgene,alsda,esa&data_type= (accessed 27 July 2026). These studies include datasets from the Rodent Research Missions (RR-5 and RR-7) as well as the Mouse Habitat Unit-2 (MHU-2) missions, which have uncovered global molecular changes in gene expression in skin tissues as well as how study design altered the experimental outcomes [335]. The skin tissues included dorsal or femoral skin across the following murine study numbers: OSD-238 (MHU-2, dorsal), OSD-239 (MHU-2, femoral), OSD-240 (RR-5, dorsal), OSD-241 (RR-5, femoral), and OSD-254 (RR-7, dorsal) [335]. Genetic strains and ages differed across these missions (i.e., MHU-2: males, C57BL/6J, 9 weeks old, ISS 30 days, euthanized 1 day post-landing; RR-5: females, BALB/c, 30 weeks old, ISS 30 days, euthanized 30 day post-recovery; and RR-7: females, C57BL/6J and C3H/HeJ, 11 weeks old, ISS for 25 or 75 days, euthanized while in orbit) [335]. Findings included reduced collagen gene expression in the MHU-2 mission (possibly due to hypergravity effects), while there was increased collagen in the RR-5 and RR-7 missions; these differences could be due to differences in the experimental approaches across these missions, including time of collection of the tissues, genetic strain, and age, amongst other differences [335]. Significantly altered genes included those linked to cell cycle, such as in chromosome segregation and DNA damage response pathways [335]. Some of these changes noted in the MHU-2 mission overlapped with data acquired from males/female astronauts on the Inspiration 4 (i4), which was a 3-day low-earth-orbit mission with 1-day post-flight tissue collection [335]. Other changes of interest to note from the i4 mission include reduced levels of epidermal profilaggrin (FLG), a critical mediator in skin barrier integrity [335]. Furthermore, CASP14 was diminished, which normally contributes to FLG breakdown [335]. It is well established that reduced levels of FLG are associated with clinical features of skin dermatoses including atopic dermatitis [335,336]. Other OMIC studies have also focused on alterations in the skin [79,337,338].

For future efforts, it is suggested to integrate artificial intelligence (AI) as well as machine learning algorithms (MLAs) to identify risks to humans and create models for the data that is available [332]. Studies that have analyzed the NASA GeneLab datasets include a systems biology approach to analyze experimental outcomes across multiple datasets to identify consistent alterations [339]. Since spaceflight missions are expensive and infrequent, it is recommended to maximize the findings obtained from one study and, hence, the knowledge gains. Furthermore, such analyses need to be shared with multiple groups around the world in a standardized manner [333].

## 7. Future Missions and Countermeasures

**Figure 7:**
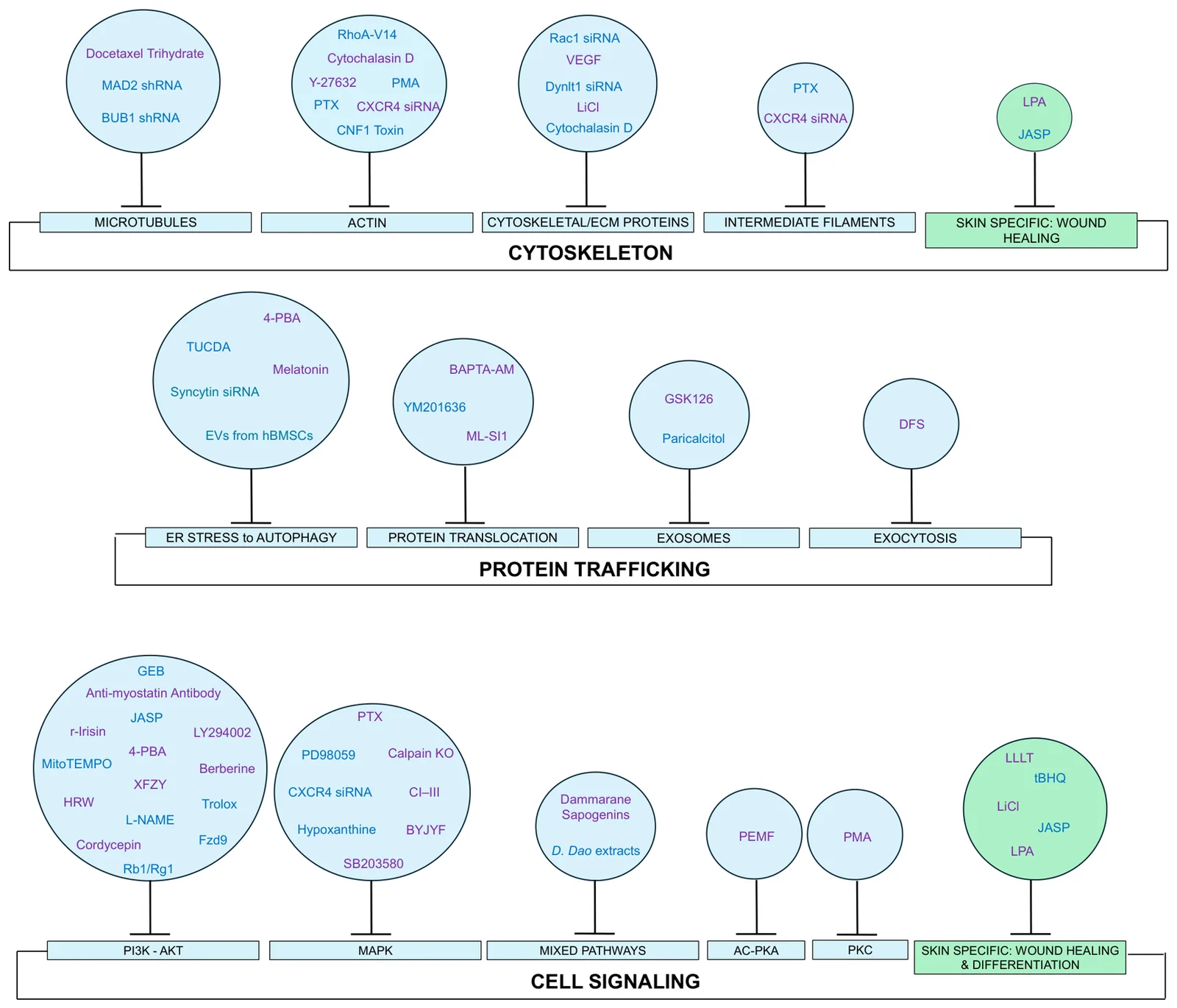
Countermeasures to real and simulated microgravity effects. Countermeasures reported to compensate changes that are induced by conditions of real and simulated microgravity are presented for the cytoskeleton (**top panel**), protein trafficking (**middle panel**), and cell signaling (**bottom panel**). The size of the bubble corresponds to the number of countermeasures identified that contribute to compensation of a particular target or pathway. The bubbles in green color are those relevant to skin model systems.

Upcoming future missions are presented in Supplementary File S17. Towards the goal of deep space exploration and manned space missions that are lengthy, it now becomes critical to develop countermeasures to oppose the detrimental effects of microgravity (and other spaceflight elements) on human health. Through the analyses presented in this manuscript, several strategies have been identified that could be developed to target key pathways and potentially offer benefits to astronauts on long-term missions. These strategies are included in the summaries presented in Supplementary Files S14–S16. As shown in Figure 7, several chemicals were reported to antagonize microgravity-induced changes on the cytoskeleton, protein trafficking events, and signaling cascades.

For the cytoskeleton, these agents included those which are known to target regulators of cytoskeletal organization, including RhoA-V14 [64], Rac1 siRNA [136], Dynlt1 siRNA [133], MAD2 shRNA [130], BUB1 shRNA [130], ROCK inhibitor (Y-27632) [66], PKC activator (PMA) [80], and CXCR4 siRNA [71]. Other agents that were shown to oppose the microgravity-induced changes on the cytoskeleton included pertussis toxin (PTX) [71], CNF1 toxin [58], VEGF [97], cytochalasin D [91,133], docetaxel trihydrate [139], LiCl [133], LPA [56], and JASP [56]. For protein trafficking, well-established inhibitors of ER stress were shown to oppose the negative consequences of microgravity, including 4-PBA [174,175,180] and TUCDA [175]; other agents included melatonin [185], extracellular vesicles (EVs) [177], and syncytin A siRNA [184]. Further, the microgravity-induced changes on protein translocation could be antagonized by BAPTA-AM [204], YM201636 [204], and ML-Sl1 [204], while GSK126 [198] and paricalcitol [198] could hinder vesicular exocytosis. Although dynamic foot stimulation (DFS) [201] could reverse some aspects of microgravity, further effort is needed to investigate how the molecular changes oppose the effects induced by microgravity. With respect to cell signaling, a large array of chemicals opposed the effects of microgravity on the PI3K-AKT signaling pathway, including r-Irisin [263], Trolox [263], L-NAME [251], Rb1 [269], Rg1 [269], cordycepin [272], hydrogen-rich water (HRW) [264], XFZY [257], MitoTEMPO [253], berberine [268], 4-PBA [253], LY294002 [251], JASP [271], GEB [265,266], and anti-myostatin antibody [255]. A subset of agents was also reported to oppose the effects of microgravity on the MAPK pathway, including PTX [71], PD98059 [236], calpain knockout (KO) [237], CI-III [237], BYJYF [239], hypoxanthine [235], SB203580 [242], and CXCR4 siRNA [71]. Other pathways including AC-PKA and PKC could be targeted with dammarane sapogenins (DS) [293], *D. Dao* extracts [213], PMA [278,279,280,282], LLLT [211], tBHQ [210], LiCl [210], JASP [56], LPA [56], and PEMF [226]. Altogether, further efforts are needed in the development of improved drug-based strategies and countermeasures to maintain health under microgravity conditions.

## 8. Limitations in the Research

There has been limited research using skin-specific cell types, although skin-relevant cell types have been assessed in response to microgravity. In this context, it is well established that certain cell lines are more easily adaptable to microgravity studies, particularly those that are used in real environments, compared to other cell types; this could be related to the proliferative capacity of the cells. Other limitations of our study are the sole use of PubMed and search terms that were applied which may have limited the research articles that were extracted. As noted earlier, the literature coverage was incomplete due to the American/Western European biases, accessibility issues, articles available only in foreign languages, as well as search engines issues.

Other limitations include variability in the experimental approaches applied, including the type of simulated microgravity and the time frame of its application. Other challenges include the density of the cultures, the passage number of the cultures, the culture apparatus utilized, hypergravity phases, vibrations, and changes in temperature across various pre-experimental phases. Well-powered studies are currently lacking; indeed, many of the current studies report low sample sizes and hence are associated with challenges in data interpretation. The reproducibility of the applied experimental approaches and resultant data are viewed to be essential to maximize the knowledge gains. Altogether, standard operating procedures (SOPs) may be valuable to support consistency in the applied methodologies and reduce data variability.

Patient diagnosis on Earth can be accomplished within a few days. However, in space, months are generally needed as a turnaround time. Therefore, methods in space need to be developed that are automated. While there have been tremendous advancements in the field of engineering and developing equipment to run aboard missions, further work in this area is essential. These include the Oxford MinION sequencer and MiniPCR^TM^, for example [340]. One also needs to carefully consider controls that will eliminate variables that may confound interpretation of the resultant data obtained (i.e., vibration effects, rotation effects). The lack of liquid nitrogen aboard spaceflight missions hinders flash freezing of collected specimens, and, thus, samples are typically placed at −80 °C, which may lead to changes in expression unrelated to the microgravity environment [333].

## Figures and Tables

**Table 1:** Frequency of microgravity methods utilized in the cytoskeleton studies. The table displays the frequency of simulated and real microgravity methods utilized within the cytoskeleton studies reviewed in addition to the PMID citation. We direct the reader to Supplementary File S5 for methodology details of the duration of the microgravity period.

Simulated Microgravity	Cytoskeleton	PMIDs
RPM	24	11955476, 16237826, 16432709, 16528471, 17180508, 17340622, 19226201, 20058807, 21246756, 24244418, 25140323, 26887372, 27581365, 27842307, 29986550, 31417174, 32240849, 17987657, 24859186, 31177466, 11919168, 29387633, 31606974, 28500304
Clinostat	9	32901501, 33925309, 10750563, 20174953, 27744595, 37795566, 27268042, 29845076, 31565646
Clinorotation	2	19730978, 16380203
Clinochip	1	28725712
RWV	6	18627022, 15308329, 16831295, 24116140, 10037426, 28518000
RCCS/HARV	4	27269634, 16160744, 10611549, 25497331
Gravite	2	31169056, 31588849,
Roller Culture Apparatus	1	23097024
Magnetic Levitation	1	16380203
Hindlimb Unloading/Tail Suspension	2	37795566, 16512669, 14715702
Head Down Bed Rest	1	35957987
Dry Immersion	0	

**Real Microgravity**	**Cytoskeleton**	**PMIDs**
Parabolic Flight	7	21865726, 22024737, 25681461, 31261642, 35887223, 23548630, 26818711
Sounding Rocket	7	23651740, 29985426, 31261642, 11538619, 26818711, 31096581, 11542625
ISS	6	28419128, 38104197, 38548798, 23658927, 26917741, 34714361, 23658927
Spaceflight Missions	4	9434637, 9707173, 17384275, 11481229
Satellites	4	11511518, 24903274, 30108515, 30521385

**Table 2:** Frequency of microgravity methods utilized in the protein trafficking studies. The table displays the frequency of simulated and real microgravity methods utilized within the protein trafficking studies reviewed in addition to the PMID citation. We direct the reader to Supplementary File S6 for methodology details of the duration of the microgravity period.

Simulated Microgravity	Protein Trafficking	PMIDs
RPM	6	26945892, 30071661, 30071661, 32143440, 32143440, 36555738
Clinostat	5	31359205, 32592441, 27070587, 40089547, 24359439
Clinorotation	1	24032680
Clinochip	0	
RWV	2	16240088, 22566481
RCCS/HARV	3	29388695, 31470364, 11409686
Gravite	1	33387250
Roller Culture Apparatus	0	
Magnetic Levitation	0	
Hindlimb Unloading/Tail Suspension	8	17705678, 3740671, 38328826, 39901193, 39456978, 32683580, 26359799, 11439724
Head Down Bed Rest	0	
Dry Immersion	1	39450600

**Real Microgravity**	**Protein Trafficking**	**PMIDs**
Parabolic Flight	1	19473213
Sounding Rocket	1	31096581
ISS	5	38104197, 36830573, 38254665, 23913861, 33669943
Spaceflight Missions	9	10958400, 2153083, 20333478, 11675122, 3041209, 35783863, 38862464, 25970640, 28542224
Satellites	0	

**Table 3:** Frequency of microgravity methods utilized in the cell signaling studies. The table displays the frequency of simulated and real microgravity methods utilized within the cell signaling studies reviewed in addition to the PMID citation. We direct the reader to Supplementary File S7 for methodology details of the duration of the microgravity period.

Simulated Microgravity	Signaling	PMIDs
RPM	13	17987657, 24859186, 31606974, 25215287, 31340547, 33467082, 35083220, 35391557, 38512830, 26576504, 16210397, 32070055, 36674696
Clinostat	13	34639043, 37795566, 12892532, 40011255, 17877609, 21287193, 22808143, 26161778, 29320953, 31431660, 37585277, 10544190, 30279524
Clinorotation	1	11542626
Clinochip	0	
RWV	4	9581798, 11928994, 14966022, 19626337
RCCS/HARV	14	37231921, 22524992, 21971552, 25804385, 27269634, 32918764, 32989050, 15644346, 28729699, 34571251, 38272357, 39193227, 10926674, 15660414
Gravite	2	33419467, 33419467
Roller Culture Apparatus	0	
Magnetic Levitation	0	
Hindlimb Unloading/Tail Suspension	23	37795566, 18511523, 30350869, 38342810, 32989050, 34689948, 40038750, 40303591, 21983076, 27562841, 30219115, 30295679, 32304741, 36646717, 36682818, 37168997, 37205911, 37758017, 39193227, 39722302, 40280637, 28611667, 35783508
Head Down Bed Rest	0	
Dry Immersion	1	39450600

**Real Microgravity**	**Signaling**	**PMIDs**
Parabolic Flight	3	2047470, 31731625, 8314874
Sounding Rocket	3	8440333, 11542626, 23651740
ISS	4	24362480, 29320953, 38548798, 36830740
Spaceflight Missions	12	24796731, 38862494, 10705996, 10949995, 10949995, 3028180, 29984852, 29984852, 25795455, 10352142, 12210720, 17404041
Satellites	1	29533735

**Table 4:** NASA GeneLab repository for skin relevant datasets. The table displays the study number, study title, organism studied, spaceflight factors, and the release date.

Study Number	Study Title	Organism	Factors	Assay Type	Release Date
OSD-6	Transcription profiling of rat keratinocytes exposed to a 56Fe ion beam	*Rattus norvegicus*	Ionizing Radiation	Transcription profiling	29 November 2007
OSD-182	Human skin fibroblast mitochondrial depletion, 0.5 Gy alpha-particle	*Homo sapiens*	Ionizing Radiation	Transcription profiling	4 April 2011
OSD-92	Response of the EPI-200 human 3-D skin model to high and low doses of protons	*Homo sapiens*	Ionizing Radiation, Time of Sample Collection After Treatment, Absorbed Radiation Dose	Transcription profiling	1 July 2011
OSD-368	Biological response to low dose of alpha-particles in a human 3-dimensional skin model, in 1 and 16 h after exposure to ionizing radiation.	*Homo sapiens*	Ionizing Radiation, Time of Sample Collection After Treatment	Transcription profiling	30 August 2011
OSD-369	Bystander responses to 0.5 Gy of alpha-particles in a human 3-dimensional skin model in 4 h after exposure to ionizing radiation	*Homo sapiens*	Ionizing Radiation	Transcription profiling	30 August 2011
OSD-367	Bystander response to 0.5 Gy of alpha-particles in a human 3-dimensional skin model in 16 h after exposure to ionizing radiation	*Homo sapiens*	Ionizing Radiation, Radiation Distance	Transcription profiling	30 August 2011
OSD-151	Bystander response to 2.5 Gy of protons in a human 3-dimensional skin model in 16 h after exposure	*Homo sapiens*	Ionizing Radiation, Radiation Distance, Absorbed Radiation Dose	Transcription profiling	30 August 2011
OSD-61	Effect of a 91 day long stay in weightlessness on the International Space Station on mouse skin physiology	*Mus musculus*	Spaceflight, Genotype	Transcription profiling	31 December 2014
OSD-116	Biological and Metabolic Response in STS-135 Space-flown Mouse Skin	*Mus musculus*	Spaceflight	Metabolite profiling, transcription profiling	28 April 2017
OSD-237	Transcriptomic analysis of skin from mice subjected to chronic low-dose radiation, hindlimb unloading or a combination of both	*Mus musculus*	Ionizing Radiation, Hindlimb Unloading	Transcription profiling	25 June 2019
OSD-238	Transcriptomic analysis of dorsal skin from mice flown on the MHU-2 mission	*Mus musculus*	Spaceflight, Diet, Altered Gravity	Transcription profiling	29 July 2019
OSD-239	Transcriptomic analysis of femoral skin from mice flown on the MHU-2 mission	*Mus musculus*	Spaceflight, Diet, Altered Gravity	Transcription profiling	29 July 2019
OSD-240	Transcriptional analysis of dorsal skin from mice flown on the RR-5 mission	*Mus musculus*	Spaceflight	Transcription profiling	12 August 2019
OSD-241	Transcriptional analysis of femoral skin from mice flown on the RR-5 mission	*Mus musculus*	Spaceflight	Transcription profiling	12 August 2019
OSD-243	Transcriptional analysis of dorsal skin from mice flown on the RR-6 mission	*Mus musculus*	Spaceflight, Duration, Dissection Condition, Euthanasia Location	Transcription profiling	26 August 2019
OSD-254	Transcriptional analysis of dorsal skin from mice flown on the RR-7 mission	*Mus musculus*	Spaceflight, Strain, Duration	Transcription profiling	8 November 2019
OSD-574	SpaceX Inspiration4 Deltoid Skin and Microbiome Data: Spatial Transcriptomics (NanoString GeoMx), Metageomics, and Metatranscriptomics	*Homo sapiens*, Microbiota Not Applicable	Spaceflight, Time	Metagenomic sequencing, transcription profiling, metatranscriptome sequencing assay	14 May 2024
OSD-572	SpaceX Inspiration4 Oral, Nasal, and Skin Metagenomic and Metatranscriptomic Microbial Swabs	Microbiota	Spaceflight, Time, Sample Location	Metagenomic sequencing, metatranscriptome sequencing assay	14 May 2024
OSD-793	Mission SpaceX CRS-16 RRRM-1 space flight induced skin genomic plasticity via an epigenetic trigger	*Mus musculus*	Spaceflight, Euthanasia Location, Dissection Condition, Duration	DNA methylation profiling, targeted transcriptome sequencing	28 November 2024

## Data Availability

Data is contained within the article or Supplementary Material.

## Supplementary Materials

The following supporting information can be downloaded at: https://www.mdpi.com/article/10.3390/ijms27177689/s1, [16,18,19,20,21,22,23,25,26,27,52,53,54,55,56,57,58,61,62,64,65,66,67,68,69,70,71,73,74,75,76,77,79,80,81,82,83,84,85,86,88,89,91,92,93,94,96,97,98,99,100,101,103,104,106,107,110,111,112,113,114,121,122,123,124,125,128,129,130,132,133,136,139,140,142,144,147,148,149,150,151,152,153,154,155,156,157,158,159,160,161,162,163,164,166,167,168,173,174,175,176,177,178,179,180,181,182,183,184,185,189,190,191,192,193,194,196,197,198,199,200,201,202,203,204,206,207,208,209,210,211,212,213,214,215,216,221,222,223,224,225,226,229,231,232,233,234,235,236,237,239,240,241,242,244,246,247,248,249,250,251,252,253,254,255,256,257,258,259,260,261,262,263,264,265,266,267,268,269,270,271,272,273,274,275,276,278,279,280,281,282,283,284,285,286,287,288,289,290,291,292,293,294,295,296,297,298,299,300,301,302,303,304,305,306,307,308,309,310,311,312,315,318,320,321,323,328,329,332,333,338,339,340,341,342,343,344,345,346,347,348,349,350,351,352,353,354,355,356,357,358,359,360,361,362,363,364,365,366,367,368,369,370,371,372,373,374,375,376,377,378,379,380,381,382,383,384].

## Abbreviations

The following abbreviations are used in this manuscript:

3-MA	3-Methyladenine
4-PBA	4-Phenylbutyric Acid
p70S6K	70 kDa Ribosomal Protein S6 Kinase
ACTA1	Actin Alpha 1
ACTA2	Actin Alpha 2
ACTN3	Actin Alpha 3
ACTB1	Actin Beta 1
ACTG2	Actin Gamma 2
ARP2	Actin-Related Protein 2
ARP3	Actin-Related Protein 3
ARPC3	Actin Related Protein 2/3 Complex Subunit 3
ATF-4	Activating Transcription Factor 4
ALB	Adenylyl Cyclase (AC); Albumin
ALP	Alkaline Phosphatase
AFP	Alpha-Fetoprotein
α-SMA	Alpha Smooth Muscle Actin
Als2	Amyotrophic Lateral Sclerosis 2 Protein
AQP2	Anti-CD3 (Leu4); Aquaporin 2
ATG4B	Autophagy-Related 4B
ATG5	Autophagy-Related 5
ATG7	Autophagy-Related 7
ATG14	Autophagy-Related 14
ATG16L1	Autophagy-Related 16 Like 1
BYJYF	Baoyuan Jieyu Formula
BAPTA-AM	BAPTA Acetoxymethyl Ester
bFGF	Basic Fibroblast Growth Factor
BNIP3	BCL2 Interacting Protein 3
BiP	Binding-Immunoglobulin Protein
CTNNB1	Catenin Beta 1
BMD	Bone Mineral Density
BMP2	Bone Morphogenetic Protein 2
BUB1	Budding Uninhibited by Benzimidazoles 1 Yeast Homolog
AD	Adherent
CXCR4	C-X-C Motif Chemokine Receptor 4
CDH1	Cadherin 1
CNN1	Calponin 1
CO_2_	Carbon Dioxide
Cat	Catalase
CAV1	Caveolin 1
CHOP	C/EBP Homologous Protein
CH	Chloral Hydrate
CLDN	Claudin
Col I	Collagen Type I
Col III	Collagen Type III
COL2A1	Collagen Type II Alpha 1 Chain
COL10A1	Collagen Type X Alpha 1 Chain
ConA	Concanavalin A
Cx43	Connexin 43
cAMP	Cyclic Adenosine Monophosphate
CREB	Cyclic AMP-Responsive Element Binding Protein
CHP	Cyclic Hydrostatic Pressure
Cyto D	Cytochalasin D
CK18	Cytokeratin 18
DNA	Deoxyribonucleic Acid
DNMT	DNA Methyltransferase
DES	Desmin
DOC	Docetaxel Trihydrate
DUSP	Dual Specificity Phosphatase
DFS	Dynamic Foot Stimulation
DNAH11	Dynein Axonemal Heavy Chain 11
DYNC2LI1	Dynein Cytoplasmic 2 Light Intermediate Chain 1
Dynlt1	Dynein Light Chain Tctex-Type 1
ER	Endoplasmic Reticulum
eNOS	Endothelial Nitric Oxide Synthase
EZH2	Enhancer of Zeste 2 Polycomb Repressive Complex 2 Subunit
EPEC	Enteropathogenic *E. coli*
EGF	Epidermal Growth Factor
EGFR	Epidermal Growth Factor Receptor
EIF2S2	Eukaryotic Initiation Factor 2 Subunit 2
eIF1a	Eukaryotic Translation Initiation Factor 1a
4EBP1	Eukaryotic Translation Initiation Factor 4E-Binding Protein 1
EVs	Extracellular Vesicles
ECM	Extracellular Matrix
FSP-1	Fibroblast-Specific Protein 1
FLG	Filaggrin
FLNA	Filamin A
FAK	Focal Adhesion Kinase
FOXO3a	Forkhead Box O3
Fzd9	Frizzled Class Receptor 9
GEB	Gastrodia Elata Blume
Rb1	Ginsenoside
Rg1	Ginsenoside
GRP78	Glucose Regulatory Protein 78
GSSG	Glutathione Disulfide
GSK3	Glycogen Synthase Kinase 3
Fmo2	Flavin-Containing Monooxygenase 2
FoxD3	Forkhead Box D3
FHL1	Four and a Half LIM Domains 1
GABARAPL1	GABA Type A Receptor-Associated Protein-Like 1
Gsr	Glutathione Disulfide Reductase
GSTA1	Glutathione S-Transferase Alpha 1
GSTA2	Glutathione S-Transferase Alpha 2
GPCR	G-Protein Coupled Receptor
G-CSF	Granulocyte Colony Stimulating Factor
GFP	Green Fluorescent Protein
GRB2	Growth Factor Receptor-Bound Protein 2
HMOX1	Heme Oxygenase 1
HARV	High-Aspect-Ratio Vessels
KDM	Histone Demethylase
HOXA9	Homeobox A9
HRW	Hydrogen-Rich Water
IRE1	Inositol-Requiring Enzyme Type 1
i4	Inspiration 4
IGF-1	Insulin-Like Growth Factor 1
ITGA2	Integrin Subunit Alpha 2
ITGB1	Integrin Subunit Beta 1
ITGB3	Integrin Subunit Beta 3
ITGB4	Integrin Subunit Beta 4
ITGB5	Integrin Subunit Beta 5
ICAM3	Intercellular Adhesion Molecule 3
IL7R	Interleukin 7 Receptor
IL13RA2	Interleukin 13 Receptor Subunit Alpha 2
IL15RA	Interleukin 15 Receptor Subunit Alpha
ICLAC	International Cell Line Authentication Committee
ISS	International Space Station
IFT88	Intraflagellar Transport 88
JAK	Janus Kinase
JASP	Jasplakinolide
KAT-NAL2	Katanin-Like 2 Proteins
KRT80	Keratin 80
KRAS	Kirsten Rat Sarcoma Viral Oncogene
LC-MS/MS	Liquid Chromatography with Tandem Mass Spectrometry
LGN	Lateral Geniculate Nucleus
LiCl	Lithium Chloride
L-NAME	L-NG-Nitro Arginine Methyl Ester
LLLT	Low-Level Light Therapy
LCP1	Lymphocyte Cytosolic Protein 1
LPA	Lysophosphatidic Acid
LAMP	Lysosomal-Associated Membrane Protein
HLA-DR	Major Histocompatibility Complex DR
MDA	Malondialdehyde
Ki67	Marker of Proliferation
MMP	Matrix Metalloproteinases
mTOR	Mechanistic Target of Rapamycin Kinase
MESP-1	Mesoderm Posterior BHLH Transcription Factor 1
LC3	Microtubule-Associated Protein 1 Light Chain 3
MTOC	Microtubule Organizing Center
MAPK	Mitogen-Activated Protein Kinase
MAPK3	Mitogen Activated Protein Kinase 3
MAPK8	Mitogen Activated Protein Kinase 8
MAPK9	Mitogen Activated Protein Kinase 9
MAPK14	Mitogen-Activated Protein Kinase 14
MAP2K1	Mitogen-Activated Protein Kinase Kinase 1
MAP2K6	Mitogen Activated Protein Kinase Kinase 6
MAD2	Mitotic Arrest Deficient 2
MCSs	Multicellular Spheroids
MYL5	Myosin Light Chain 5
NASA	National Aeronautics and Space Administration
NGF	Nerve Growth Factor
NCAM1	Neural Cell Adhesion Molecular 1
NO	Nitric Oxide
NFE2L2	NFE-Like BZIP Transcription Factor 2
OCLN	Occludin
OCT-4	Octamer-Binding Protein 4
OCN	Osteocalcin
OPN	Osteopontin
PI3K	Phosphatidylinositol-3-Kinase
PRDX2	Peroxiredoxin 2
Prdx6-ps1	Peroxiredoxin 6, Pseudogene 1
PBMCs	Peripheral Blood Mononuclear Cells
PTX	Pertussis Toxin
PDE	Phosphodiesterase
PMA	Phorbol Myristate Acetate
PTEN	Phosphatase and Tensin Homolog
PIK3CA	Phosphatidylinositol-4,5-Bisphosphate 3-Kinase
PLC	Phospholipase C
PHA	Phytohaemagglutinin
PDGF-βR	Platelet-Derived Growth Factor Receptor
PPC	Posterior Parietal Cortex
PSD-95	Post-Synaptic Density Protein 95
Ptgs2	Prostaglandin-Endoperoxide Synthase 2
PKA	Protein Kinase A
AKT	Protein Kinase B
AKT1	Protein Kinase B Alpha
AKT3	Protein Kinase B Gamma
PKC	Protein Kinase C
PERK	Protein Kinase RNA-Like Endoplasmic Reticulum Kinase
PYK2	Protein Tyrosine Kinase 2-Beta
PMID	PubMed Identifier
PEMF	Pulsed Electromagnetic Field
RPM	Random Positioning Machine
RICTOR	Rapamycin-Insensitive Companion of MTOR
RASA1	RAS p21 Protein Activator 1
ROS	Reactive Oxygen Species
qPCR	Real-Time Polymerase Chain Reaction
RANKL	Receptor Activator of Nuclear Factor Kappa-B Ligand
ROCK	Rho-Associated Coiled-Coil-Containing Protein Kinase
RNA	Ribonucleic Acid
RPS6	Ribosomal Protein S6
S6K1	Ribosomal Protein S6 Kinase 1
RCCS	Rotary Cell Culture System
RWV	Rotating Wall Vessel
RSOC	Rotation System of Culture
RUNX2	Runt-Related Transcription Factor 2
siRNA	Short Inhibitory RNA
STAT	Signal Transducer and Activator of Transcription
SM-MHC	Smooth Muscle Myosin Heavy Chain
SLC35A1	Solute Carrier Family 35 Member A1
SOS	Son of Sevenless Homolog 1
Shc	Src Homology 2 Domain-Containing Transforming Protein
Sox9	SRY-Box Transcription Factor 9
Sox10	SRY-Box Transcription Factor 10
ST3GAL1	ST3 Beta-Galactoside Alpha-2,3-Sialyltransferase 1
SAPK	Stress-Activated Protein Kinase
SOD	Superoxide Dismutase
SVIL	Supervillin
SUZ12	Suppressor of Zeste 12 Homolog
SCNA	Synuclein Alpha
TLN2	Talin 2
TLR	Toll-Like Receptor
TUDCA	Tauroursodeoxycholic Acid
TERT	Telomerase Reverse Transcriptase
tBHQ	Tert-Butylhydroquinone
3D	Three-Dimensional
TPA	12-*O*-tetradecanoylphorbol-13-acetate
TAZ	Transcriptional Coactivator with PDZ-Binding Motif
TEWL	Transepidermal Water Loss
TGFβ	Transforming Growth Factor Beta
TRPML1	Transient Receptor Potential Channel Mucolipin 1
TGM2	Transglutaminase 2
TEM	Transmission Electron Microscopy
TSC2	Tuberous Sclerosis 2 Protein
TUBA1A	Tubulin Alpha 1a
TUBB	Tubulin Beta
TUBB1	Tubulin Beta 1
TUBB2	Tubulin Beta 2
Tuj1	Tubulin Beta 3 Class III
TUBB4B	Tubulin Beta 4B
TUBB8	Tubulin Beta 8
TSG101	Tumor Susceptibility 101
2D	Two-Dimensional
ULK1	Unc-51-Like Autophagy Activating Kinase 1
UPR	Unfolded Protein Response
UVRAG	UV Radiation Resistance-Associated Gene Protein
VEGFR	Vascular Endothelial Growth Factor Receptor
VAMPA3	Vesicle-Associated Membrane Protein 3
VGLUT	Vesicular Glutamate Transporter
VCL	Vinculin
VDR	Vitamin D Receptor
VIM	Vimentin
WBC	White Blood Cells
WNT5a	Wnt Family Member 5
WNT9a	Wnt Family Member 9a
XBP1	X-Box Binding Protein 1
XFZY	Xuefuzhuyu
YAP	Yes1-Associated Protein
