# Supplementary material for: Impact of Microgravity on Cytoskeletal Dynamics, Protein Transport, and Signaling Networks: Potential Therapeutic Opportunities for Skin Health
Source: Int J Mol Sci. 2026 Aug 27;27(17):7689. doi: 10.3390/ijms27177689 (PMC13565916; doi:10.3390/ijms27177689)
Supplement: Supplementary file 1 [file ijms-27-07689-s001.zip › Supplementary_captions.pdf]

SUPPLEMENTARY FILE LEGENDS

Supplementary File S1. PubMed Extracted Articles using Cytoskeleton Search Terms. This file contains two worksheets, with the first sheet displaying search terms utilized to identify articles relevant to cytoskeletal alterations under microgravity conditions. The first worksheet titled "Cytoskeleton" includes the date the search was performed for each keyword, the total number of hits, and relevant hits after reviewing abstracts. In addition, PMIDs listed in column E are those that were identified as relevant. The second worksheet titled "Cytoskeleton_Rep_Removed" includes a complete list of PMIDs in numerical order and a column in which replicates were removed. Each citation was comprehensively analyzed. Two independent PubMed reviews were performed to identify article corrections and retractions. These independent reviews were conducted two months apart. Column F displays articles that were eliminated following a full text review.[1-85]

Supplementary File S2. PubMed Extracted Articles using Protein Trafficking Search Terms. This file contains two worksheets, with the first sheet displaying search terms utilized to identify articles relevant to protein trafficking under microgravity conditions. The first worksheet titled "Protein Trafficking" includes the date the search was performed for each keyword, the total number of hits, and relevant hits after reviewing abstracts. In addition, PMIDs listed in column E are those that were identified as relevant. The second worksheet titled "Protein_Trafficking_Rep_Removed" includes a complete list of PMIDs in numerical order and a column in which replicates were removed. Each citation was comprehensively analyzed. Two independent PubMed reviews were performed to identify article corrections and retractions. These independent reviews were conducted two months apart. Column F displays articles that were eliminated following a full text review. [67, 83, 86-129]

Supplementary File S3. PubMed Extracted Articles using Cell Signaling Search Terms. This file contains two worksheets, with the first sheet displaying search terms utilized to identify articles relevant to cell signaling under microgravity conditions. The first worksheet titled "Cell Signaling" includes the date the search was performed for each keyword, the total number of hits, and relevant hits after reviewing abstracts. In addition, PMIDs listed in column E are those that were identified as relevant. The second worksheet titled "Cell_Signaling_Rep_Removed" includes a complete list of PMIDs in numerical order and a column in which replicates were removed. Each citation was comprehensively analyzed. Two independent PubMed reviews were performed to identify article corrections and retractions. These independent reviews were conducted two months apart. Column F displays articles that were eliminated following a full text review. [29, 40, 44, 53, 74, 82, 84, 126, 130-208]

Supplementary File S4. PubMed Extracted Articles using Cell Culture and OMICS Relevant Search Terms. This file contains four worksheets, with the first and third sheet displaying search terms utilized to identify articles relevant to cell culture methods and OMICS under microgravity conditions. The first worksheet titled "Data Mining" includes the date the search was performed for each keyword, the total number of hits, and relevant hits after reviewing abstracts. In addition, PMIDs listed in column E are those that were identified as relevant. The second worksheet titled "Data_Mining_Rep_Removed" includes a complete list of PMIDs in numerical order and a column in which replicates were removed. The third worksheet titled "ISS Cell Culture Methods" includes the date the search was performed for each keyword, the total number of hits, and relevant hits after reviewing abstracts. In addition, PMIDs listed in column E are those that were identified as relevant. The second worksheet titled "Culture_Rep_Removed" includes a complete list of PMIDs in numerical order and a column in which replicates were removed. Each citation was comprehensively analyzed. Two independent PubMed reviews were performed to identify article corrections and retractions. These independent reviews were conducted two months apart. Column F displays articles that were eliminated following a full text review. [51, 79, 111, 123, 125, 202, 209-239]

Supplementary File S5. Microgravity Methods Utilized in the Cytoskeleton Studies. Methodological details pertaining to each PMID citation are presented including the model system, type of real or simulated microgravity, and microgravity time frame are presented. The first worksheet presents these elements for simulated microgravity while the second worksheet presents these elements for real microgravity. Please refer to Table 1 for a summary. [2-6, 9-11, 13, 14, 17-19, 21-78, 80-84, 240, 241]

Supplementary File S6. Microgravity Methods Utilized in the Protein Trafficking Studies. Methodological details pertaining to each PMID citation are presented including the model system, type of real or simulated

microgravity, and microgravity time frame are presented. The first worksheet presents these elements for simulated microgravity while the second worksheet presents these elements for real microgravity. Please refer to Table 2 for a summary. [67, 83, 86-91, 94-122, 126-129, 131, 242]

Supplementary File S7. Microgravity Methods Utilized in the Cell Signaling Studies. Methodological details pertaining to each PMID citation are presented including the model system, type of real or simulated microgravity, and microgravity time frame are presented. The first worksheet presents these elements for simulated microgravity while the second worksheet presents these elements for real microgravity. Please refer to Table 3 for a summary. [29, 40, 44, 53, 74, 82, 84, 126, 130, 132-155, 157-180, 182-190, 192-197, 199-208, 243-247]

Supplementary File S8. Location of Real Microgravity Experiments Pertaining to the Cytoskeleton Studies. The affiliation, launch site, and mission information are provided for each PMID citation.[2, 3, 9-11, 28, 36, 37, 39-41, 45, 48, 49, 51, 57, 63, 65-67, 70, 78, 80, 83, 84, 241]

Supplementary File S9. Location of Real Microgravity Experiments Pertaining to the Protein Trafficking Studies. The affiliation, launch site, and mission information are provided for each PMID citation. [67, 83, 86-88, 91, 96, 97, 99, 102, 106, 115, 116, 118, 120, 122, 135]

Supplementary File S10. Location of Real Microgravity Experiments Pertaining to the Cell Signaling Studies. The affiliation, launch site, and mission information are provided for each PMID citation. [40, 84, 130, 132, 133, 135, 137, 139, 140, 142, 148, 157, 158, 160, 167-169, 175, 192, 202, 247]

Supplementary File S11. Secondary PubMed Extracted Articles using Cytoskeleton Search Terms. This file contains two worksheets, with the first sheet displaying search terms utilized to identify articles relevant to cytoskeletal alterations under microgravity conditions. The first worksheet titled "Cytoskeleton" includes the date the search was performed for each keyword, the total number of hits, and relevant hits after reviewing abstracts. In addition, PMIDs listed in column E are those that were identified as relevant. The second worksheet titled "Added_Citations_Rep_Removed" includes a complete list of PMIDs in numerical order and a column in which replicates were removed. Each citation was comprehensively analyzed. [248-268]

Supplementary File S12. Secondary PubMed Extracted Articles using Protein Trafficking Search Terms. This file contains two worksheets, with the first sheet displaying search terms utilized to identify articles relevant to protein trafficking under microgravity conditions. The first worksheet titled "Protein Trafficking" includes the date the search was performed for each keyword, the total number of hits, and relevant hits after reviewing abstracts. In addition, PMIDs listed in column E are those that were identified as relevant. The second worksheet titled "Added_Citations_Rep_Removed" includes a complete list of PMIDs in numerical order and a column in which replicates were removed. Each citation was comprehensively analyzed. [269-271]

Supplementary File S13. Secondary PubMed Extracted Articles using Cell Signaling Search Terms. This file contains two worksheets, with the first sheet displaying search terms utilized to identify articles relevant to cell signaling under microgravity conditions. The first worksheet titled "Cell Signaling" includes the date the search was performed for each keyword, the total number of hits, and relevant hits after reviewing abstracts. In addition, PMIDs listed in column E are those that were identified as relevant. The second worksheet titled "Added_Citations_Rep_Removed" includes a complete list of PMIDs in numerical order and a column in which replicates were removed. Each citation was comprehensively analyzed. [69, 251, 269, 271-277]

Supplementary File S14. Comprehensive Summary of Cytoskeletal Outcomes from the Studies Analyzed. An organized summary of key findings are presented with respect to the cytoskeletal outcomes. The dataset is arranged into the following six worksheets: (a) skin specific, (b) mixed study, (c) actin only, (d) microtubule only, (e) intermediate filament only, and (f) associated cytoskeletal proteins. Each sheet presents details for "PMID", "publication date", "model system", "cell line authenticity", "microgravity (real/simulated)", "microgravity details", "outcome", and "recovery". Cell line authenticity was verified using the ICLAC database. [2-6, 9-11, 13-15, 17-19, 21-45, 47-78, 80-84, 241]

Supplementary File S15. Comprehensive Summary of Protein Trafficking Outcomes from the Studies Analyzed. An organized summary of key findings are presented with respect to the protein trafficking outcomes.

The dataset is arranged into the following seven worksheets: (a) organellar alterations,(b) ER stress and autophagy, (c) trafficking, (d) PTMs (glycosylation), (e) secretion, (f) vesicles, and (g) endocytosis. Each sheet presents details for "PMID", "publication date", "model system", "cell line authenticity", "microgravity (real/simulated)", "microgravity details", "outcome", and "recovery". Cell line authenticity was verified using the ICLAC database. [67, 83, 86-91, 94-122, 125-129]

Supplementary File S16. Comprehensive Summary of Cell Signaling Outcomes from the Studies Analyzed. An organized summary of key findings are presented with respect to the cell signaling outcomes. The dataset is arranged into the following ten worksheets: (a) skin relevant, (b) EGFR, (c) GPCR, (d) PLC, (e) AC-PKA, (f) JAK-STAT, (g) MAPK, (h) PI3K-AKT, (i) PKC, and (j) mixed pathways. Each sheet presents details for "PMID", "publication date", "model system", "cell line authenticity", "microgravity (real/simulated)", "microgravity details", "outcome", and "recovery". Cell line authenticity was verified using the ICLAC database. [29, 40, 44, 53, 74, 82, 84, 126, 130, 132-155, 157-180, 182-190, 192-197, 199-208, 243-245, 247]

Supplementary File 17. Future NASA Spaceflight Missions. Upcoming spaceflight missions are presented as derived from https://www.nasa.gov/mission.

Table S1. Frequency of Model Systems used in the Cytoskeleton Studies. The table displays the frequency of the various model systems reported from the Cytoskeleton studies in addition to the PMID citation. [2-6, 9-11, 13-15, 17-19, 21-78, 80-84, 241]

Table S2. Frequency of Model Systems used in the Protein Trafficking Studies. The table displays the frequency of the various model systems reported from the Protein Trafficking studies in addition to the PMID citation. [67, 83, 86-91, 94-122, 126-129, 134]

Table S3. Frequency of Model Systems used in the Cell Signaling Studies. The table displays the frequency of the various model systems reported from the Cell Signaling studies in addition to the PMID citation. [29, 40, 44, 53, 74, 82, 84, 126, 130, 132-155, 157-180, 182-190, 192-197, 199-208, 243-245, 247]

REFERENCES

1. Rijken, P. J.; de Groot, R. P.; Briegleb, W.; Kruijer, W.; Verkleij, A. J.; Boonstra, J.; de Laat, S. W., Epidermal growth factor-induced cell rounding is sensitive to simulated microgravity. *Aviat Space Environ Med* **1991,** 62, (1), 32-6.

2. Piepmeier, E. H.; Kalns, J. E.; McIntyre, K. M.; Lewis, M. L., Prolonged weightlessness affects promyelocytic multidrug resistance. *Exp Cell Res* **1997,** 237, (2), 410-8.

3. Lewis, M. L.; Reynolds, J. L.; Cubano, L. A.; Hatton, J. P.; Lawless, B. D.; Piepmeier, E. H., Spaceflight alters microtubules and increases apoptosis in human lymphocytes (Jurkat). *FASEB J* **1998,** 12, (11), 1007-18.

4. Margolis, L.; Hatfill, S.; Chuaqui, R.; Vocke, C.; Emmert-Buck, M.; Linehan, W. M.; Duray, P. H., Long term organ culture of human prostate tissue in a NASA-designed rotating wall bioreactor. *J Urol* **1999,** 161, (1), 290-7.

5. Doolin, E. J.; Geldziler, B.; Strande, L.; Kain, M.; Hewitt, C., Effects of microgravity on growing cultured skin constructs. *Tissue Eng* **1999,** 5, (6), 573-82.

6. Sarkar, D.; Nagaya, T.; Koga, K.; Nomura, Y.; Gruener, R.; Seo, H., Culture in vector-averaged gravity under clinostat rotation results in apoptosis of osteoblastic ROS 17/2.8 cells. *J Bone Miner Res* **2000,** 15, (3), 489-98.

7. Papaseit, C.; Pochon, N.; Tabony, J., Microtubule self-organization is gravity-dependent. *Proc Natl Acad Sci U S A* **2000,** 97, (15), 8364-8.

8. Vassy, J.; Portet, S.; Beil, M.; Millot, G.; Fauvel-Lafeve, F.; Karniguian, A.; Gasset, G.; Irinopoulou, T.; Calvo, F.; Rigaut, J. P.; Schoevaert, D., The effect of weightlessness on cytoskeleton architecture and proliferation of human breast cancer cell line MCF-7. *FASEB J* **2001,** 15, (6), 1104-6.

9. Lewis, M. L.; Cubano, L. A.; Zhao, B.; Dinh, H. K.; Pabalan, J. G.; Piepmeier, E. H.; Bowman, P. D., cDNA microarray reveals altered cytoskeletal gene expression in space-flown leukemic T lymphocytes (Jurkat). *FASEB J* **2001,** 15, (10), 1783-5.

10. Guignandon, A.; Lafage-Proust, M. H.; Usson, Y.; Laroche, N.; Caillot-Augusseau, A.; Alexandre, C.; Vico, L., Cell cycling determines integrin-mediated adhesion in osteoblastic ROS 17/2.8 cells exposed to space-related conditions. *FASEB J* **2001,** 15, (11), 2036-8.

11. Sciola, L.; Cogoli-Greuter, M.; Cogoli, A.; Spano, A.; Pippia, P., Influence of microgravity on mitogen binding and cytoskeleton in Jurkat cells. *Adv Space Res* **1999,** 24, (6), 801-5.

12. Tabony, J.; Pochon, N.; Papaseit, C., Microtubule self-organisation depends upon gravity. *Adv Space Res* **2001,** 28, (4), 529-35.

13. Grimm, D.; Bauer, J.; Kossmehl, P.; Shakibaei, M.; Schoberger, J.; Pickenhahn, H.; Schulze-Tanzil, G.; Vetter, R.; Eilles, C.; Paul, M.; Cogoli, A., Simulated microgravity alters differentiation and increases apoptosis in human follicular thyroid carcinoma cells. *FASEB J* **2002,** 16, (6), 604-6.

14. Uva, B. M.; Masini, M. A.; Sturla, M.; Prato, P.; Passalacqua, M.; Giuliani, M.; Tagliafierro, G.; Strollo, F., Clinorotation-induced weightlessness influences the cytoskeleton of glial cells in culture. *Brain Res* **2002,** 934, (2), 132-9.

15. Nikawa, T.; Ishidoh, K.; Hirasaka, K.; Ishihara, I.; Ikemoto, M.; Kano, M.; Kominami, E.; Nonaka, I.; Ogawa, T.; Adams, G. R.; Baldwin, K. M.; Yasui, N.; Kishi, K.; Takeda, S., Skeletal muscle gene expression in space-flown rats. *FASEB J* **2004,** 18, (3), 522-4.

16. Vassy, J.; Portet, S.; Beil, M.; Millot, G.; Fauvel-Lafeve, F.; Gasset, G.; Schoevaert, D., Weightlessness acts on human breast cancer cell line MCF-7. *Adv Space Res* **2003,** 32, (8), 1595-603.

17. Plett, P. A.; Abonour, R.; Frankovitz, S. M.; Orschell, C. M., Impact of modeled microgravity on migration, differentiation, and cell cycle control of primitive human hematopoietic progenitor cells. *Exp Hematol* **2004,** 32, (8), 773-81.

18. Meyers, V. E.; Zayzafoon, M.; Douglas, J. T.; McDonald, J. M., RhoA and cytoskeletal disruption mediate reduced osteoblastogenesis and enhanced adipogenesis of human mesenchymal stem cells in modeled microgravity. *J Bone Miner Res* **2005,** 20, (10), 1858-66.

19. Infanger, M.; Kossmehl, P.; Shakibaei, M.; Schulze-Tanzil, G.; Cogoli, A.; Faramarzi, S.; Bauer, J.; Curcio, F.; Paul, M.; Grimm, D., Longterm conditions of mimicked weightlessness influences the cytoskeleton in thyroid cells. *J Gravit Physiol* **2004,** 11, (2), P169-72.

20. Meloni, M. A.; Galleri, G.; Camboni, M. G.; Pippia, P.; Cogoli, A.; Cogoli-Greuter, M., Modeled microgravity affects motility and cytoskeletal structures. *J Gravit Physiol* **2004,** 11, (2), P197-8.

21. Glade, N.; Beaugnon, E.; Tabony, J., Ground-based methods reproduce space-flight experiments and show that weak vibrations trigger microtubule self-organisation. *Biophys Chem* **2006,** 121, (1), 1-6.

22. Infanger, M.; Kossmehl, P.; Shakibaei, M.; Bauer, J.; Kossmehl-Zorn, S.; Cogoli, A.; Curcio, F.; Oksche, A.; Wehland, M.; Kreutz, R.; Paul, M.; Grimm, D., Simulated weightlessness changes the cytoskeleton and extracellular matrix proteins in papillary thyroid carcinoma cells. *Cell Tissue Res* **2006,** 324, (2), 267-77.

23. Sarkar, P.; Sarkar, S.; Ramesh, V.; Hayes, B. E.; Thomas, R. L.; Wilson, B. L.; Kim, H.; Barnes, S.; Kulkarni, A.; Pellis, N.; Ramesh, G. T., Proteomic analysis of mice hippocampus in simulated microgravity environment. *J Proteome Res* **2006,** 5, (3), 548-53.

24. Infanger, M.; Kossmehl, P.; Shakibaei, M.; Baatout, S.; Witzing, A.; Grosse, J.; Bauer, J.; Cogoli, A.; Faramarzi, S.; Derradji, H.; Neefs, M.; Paul, M.; Grimm, D., Induction of three-dimensional assembly and increase in apoptosis of human endothelial cells by simulated microgravity: impact of vascular endothelial growth factor. *Apoptosis* **2006,** 11, (5), 749-64.

25. Maier, J. A., Impact of simulated microgravity on cell cycle control and cytokine release by U937 cells. *Int J Immunopathol Pharmacol* **2006,** 19, (2), 279-86.

26. Meloni, M. A.; Galleri, G.; Pippia, P.; Cogoli-Greuter, M., Cytoskeleton changes and impaired motility of monocytes at modelled low gravity. *Protoplasma* **2006,** 229, (2-4), 243-9.

27. Infanger, M.; Ulbrich, C.; Baatout, S.; Wehland, M.; Kreutz, R.; Bauer, J.; Grosse, J.; Vadrucci, S.; Cogoli, A.; Derradji, H.; Neefs, M.; Kusters, S.; Spain, M.; Paul, M.; Grimm, D., Modeled gravitational unloading induced downregulation of endothelin-1 in human endothelial cells. *J Cell Biochem* **2007,** 101, (6), 1439-55.

28. Kumei, Y.; Morita, S.; Katano, H.; Akiyama, H.; Hirano, M.; Oyha, K.; Shimokawa, H., Microgravity signal ensnarls cell adhesion, cytoskeleton, and matrix proteins of rat osteoblasts: osteopontin, CD44, osteonectin, and alpha-tubulin. *Ann N Y Acad Sci* **2006,** 1090, 311-7.

29. Loesberg, W. A.; Walboomers, X. F.; van Loon, J. J.; Jansen, J. A., Simulated microgravity activates MAPK pathways in fibroblasts cultured on microgrooved surface topography. *Cell Motil Cytoskeleton* **2008,** 65, (2), 116-29.

30. Clejan, S.; O'Connor, K. C.; Cowger, N. L.; Cheles, M. K.; Haque, S.; Primavera, A. C., Effects of simulated microgravity on DU 145 human prostate carcinoma cells. *Biotechnol Bioeng* **1996,** 50, (5), 587-97.

31. Grimm, D.; Infanger, M.; Westphal, K.; Ulbrich, C.; Pietsch, J.; Kossmehl, P.; Vadrucci, S.; Baatout, S.; Flick, B.; Paul, M.; Bauer, J., A delayed type of three-dimensional growth of human endothelial cells under simulated weightlessness. *Tissue Eng Part A* **2009,** 15, (8), 2267-75.

32. Li, J.; Zhang, S.; Chen, J.; Du, T.; Wang, Y.; Wang, Z., Modeled microgravity causes changes in the cytoskeleton and focal adhesions, and decreases in migration in malignant human MCF-7 cells. *Protoplasma* **2009,** 238, (1-4), 23-33.

33. Gershovich, P. M.; Gershovich Iu, G.; Buravkova, L. B., [Cytoskeleton structures and adhesion properties of human stromal precursors under conditions of simulated microgravity]. *Tsitologiia* **2009,** 51, (11), 896-904.

34. Siamwala, J. H.; Reddy, S. H.; Majumder, S.; Kolluru, G. K.; Muley, A.; Sinha, S.; Chatterjee, S., Simulated microgravity perturbs actin polymerization to promote nitric oxide-associated migration in human immortalized Eahy926 cells. *Protoplasma* **2010,** 242, (1-4), 3-12.

35. Meloni, M. A.; Galleri, G.; Pani, G.; Saba, A.; Pippia, P.; Cogoli-Greuter, M., Space flight affects motility and cytoskeletal structures in human monocyte cell line J-111. *Cytoskeleton (Hoboken)* **2011,** 68, (2), 125-37.

36. Ulbrich, C.; Pietsch, J.; Grosse, J.; Wehland, M.; Schulz, H.; Saar, K.; Hubner, N.; Hauslage, J.; Hemmersbach, R.; Braun, M.; van Loon, J.; Vagt, N.; Egli, M.; Richter, P.; Einspanier, R.; Sharbati, S.; Baltz, T.; Infanger, M.; Ma, X.; Grimm, D., Differential gene regulation under altered gravity conditions in follicular thyroid cancer cells: relationship between the extracellular matrix and the cytoskeleton. *Cell Physiol Biochem* **2011,** 28, (2), 185-98.

37. Grosse, J.; Wehland, M.; Pietsch, J.; Ma, X.; Ulbrich, C.; Schulz, H.; Saar, K.; Hubner, N.; Hauslage, J.; Hemmersbach, R.; Braun, M.; van Loon, J.; Vagt, N.; Infanger, M.; Eilles, C.; Egli, M.; Richter, P.; Baltz, T.; Einspanier, R.; Sharbati, S.; Grimm, D., Short-term weightlessness produced by parabolic flight maneuvers altered gene expression patterns in human endothelial cells. *FASEB J* **2012,** 26, (2), 639-55.

38. Kang, H.; Fan, Y.; Sun, A.; Jia, X.; Deng, X., Simulated microgravity exposure modulates the phenotype of cultured vascular smooth muscle cells. *Cell Biochem Biophys* **2013,** 66, (1), 121-30.

39. Wehland, M.; Ma, X.; Braun, M.; Hauslage, J.; Hemmersbach, R.; Bauer, J.; Grosse, J.; Infanger, M.; Grimm, D., The impact of altered gravity and vibration on endothelial cells during a parabolic flight. *Cell Physiol Biochem* **2013,** 31, (2-3), 432-51.

40. Tauber, S.; Hauschild, S.; Crescio, C.; Secchi, C.; Paulsen, K.; Pantaleo, A.; Saba, A.; Buttron, I.; Thiel, C. S.; Cogoli, A.; Pippia, P.; Ullrich, O., Signal transduction in primary human T lymphocytes in altered gravity - results of the MASER-12 suborbital space flight mission. *Cell Commun Signal* **2013,** 11, (1), 32.

41. Kapitonova, M. Y.; Kuznetsov, S. L.; Froemming, G. R.; Muid, S.; Nor-Ashikin, M. N.; Otman, S.; Shahir, A. R.; Nawawi, H., Effects of space mission factors on the morphology and function of endothelial cells. *Bull Exp Biol Med* **2013,** 154, (6), 796-801.

42. Wei, L.; Diao, Y.; Qi, J.; Khokhlov, A.; Feng, H.; Yan, X.; Li, Y., Effect of change in spindle structure on proliferation inhibition of osteosarcoma cells and osteoblast under simulated microgravity during incubation in rotating bioreactor. *PLoS One* **2013,** 8, (10), e76710.

43. Aleshcheva, G.; Sahana, J.; Ma, X.; Hauslage, J.; Hemmersbach, R.; Egli, M.; Infanger, M.; Bauer, J.; Grimm, D., Changes in morphology, gene expression and protein content in chondrocytes cultured on a random positioning machine. *PLoS One* **2013,** 8, (11), e79057.

44. Beck, M.; Moreels, M.; Quintens, R.; Abou-El-Ardat, K.; El-Saghire, H.; Tabury, K.; Michaux, A.; Janssen, A.; Neefs, M.; Van Oostveldt, P.; De Vos, W. H.; Baatout, S., Chronic exposure to simulated space conditions predominantly affects cytoskeleton remodeling and oxidative stress response in mouse fetal fibroblasts. *Int J Mol Med* **2014,** 34, (2), 606-15.

45. Guignandon, A.; Faure, C.; Neutelings, T.; Rattner, A.; Mineur, P.; Linossier, M. T.; Laroche, N.; Lambert, C.; Deroanne, C.; Nusgens, B.; Demets, R.; Colige, A.; Vico, L., Rac1 GTPase silencing counteracts microgravity-induced effects on osteoblastic cells. *FASEB J* **2014,** 28, (9), 4077-87.

46. Ferranti, F.; Caruso, M.; Cammarota, M.; Masiello, M. G.; Corano Scheri, K.; Fabrizi, C.; Fumagalli, L.; Schiraldi, C.; Cucina, A.; Catizone, A.; Ricci, G., Cytoskeleton modifications and autophagy induction in TCam-2 seminoma cells exposed to simulated microgravity. *Biomed Res Int* **2014,** 2014, 904396.

47. Li, H.; Dai, Y.; Shu, J.; Yu, R.; Guo, Y.; Chen, J., Spheroid cultures promote the stemness of corneal stromal cells. *Tissue Cell* **2015,** 47, (1), 39-48.

48. Aleshcheva, G.; Wehland, M.; Sahana, J.; Bauer, J.; Corydon, T. J.; Hemmersbach, R.; Frett, T.; Egli, M.; Infanger, M.; Grosse, J.; Grimm, D., Moderate alterations of the cytoskeleton in human chondrocytes after short-term microgravity produced by parabolic flight maneuvers could be prevented by up-regulation of BMP-2 and SOX-9. *FASEB J* **2015,** 29, (6), 2303-14.

49. Corydon, T. J.; Kopp, S.; Wehland, M.; Braun, M.; Schutte, A.; Mayer, T.; Hulsing, T.; Oltmann, H.; Schmitz, B.; Hemmersbach, R.; Grimm, D., Alterations of the cytoskeleton in human cells in space proved by life-cell imaging. *Sci Rep* **2016,** 6, 20043.

50. Zhao, T.; Tang, X.; Umeshappa, C. S.; Ma, H.; Gao, H.; Deng, Y.; Freywald, A.; Xiang, J., Simulated Microgravity Promotes Cell Apoptosis Through Suppressing Uev1A/TICAM/TRAF/NF-kappaB-Regulated Anti-Apoptosis and p53/PCNA- and ATM/ATR-Chk1/2-Controlled DNA-Damage Response Pathways. *J Cell Biochem* **2016,** 117, (9), 2138-48.

51. Zhang, Y.; Lu, T.; Wong, M.; Wang, X.; Stodieck, L.; Karouia, F.; Story, M.; Wu, H., Transient gene and microRNA expression profile changes of confluent human fibroblast cells in spaceflight. *FASEB J* **2016,** 30, (6), 2211-24.

52. Wang, X.; Du, J.; Wang, D.; Zeng, F.; Wei, Y.; Wang, F.; Feng, C.; Li, N.; Dai, R.; Deng, Y.; Quan, Z.; Qing, H., Effects of simulated microgravity on human brain nervous tissue. *Neurosci Lett* **2016,** 627, 199-204.

53. Lin, S. C.; Gou, G. H.; Hsia, C. W.; Ho, C. W.; Huang, K. L.; Wu, Y. F.; Lee, S. Y.; Chen, Y. H., Simulated Microgravity Disrupts Cytoskeleton Organization and Increases Apoptosis of Rat Neural Crest Stem Cells Via Upregulating CXCR4 Expression and RhoA-ROCK1-p38 MAPK-p53 Signaling. *Stem Cells Dev* **2016,** 25, (15), 1172-93.

54. Janmaleki, M.; Pachenari, M.; Seyedpour, S. M.; Shahghadami, R.; Sanati-Nezhad, A., Impact of Simulated Microgravity on Cytoskeleton and Viscoelastic Properties of Endothelial Cell. *Sci Rep* **2016,** 6, 32418.

55. Mao, X.; Chen, Z.; Luo, Q.; Zhang, B.; Song, G., Simulated microgravity inhibits the migration of mesenchymal stem cells by remodeling actin cytoskeleton and increasing cell stiffness. *Cytotechnology* **2016,** 68, (6), 2235-2243.

56. Corydon, T. J.; Mann, V.; Slumstrup, L.; Kopp, S.; Sahana, J.; Askou, A. L.; Magnusson, N. E.; Echegoyen, D.; Bek, T.; Sundaresan, A.; Riwaldt, S.; Bauer, J.; Infanger, M.; Grimm, D., Reduced Expression of Cytoskeletal and Extracellular Matrix Genes in Human Adult Retinal Pigment Epithelium Cells Exposed to Simulated Microgravity. *Cell Physiol Biochem* **2016,** 40, (1-2), 1-17.

57. Tauber, S.; Lauber, B. A.; Paulsen, K.; Layer, L. E.; Lehmann, M.; Hauschild, S.; Shepherd, N. R.; Polzer, J.; Segerer, J.; Thiel, C. S.; Ullrich, O., Cytoskeletal stability and metabolic alterations in primary human macrophages in long-term microgravity. *PLoS One* **2017,** 12, (4), e0175599.

58. Shi, W.; Xie, Y.; He, J.; Zhou, J.; Gao, Y.; Wei, W.; Ding, N.; Ma, H.; Xian, C. J.; Chen, K.; Wang, J., Microgravity induces inhibition of osteoblastic differentiation and mineralization through abrogating primary cilia. *Sci Rep* **2017,** 7, (1), 1866.

59. Mellor, L. F.; Steward, A. J.; Nordberg, R. C.; Taylor, M. A.; Loboa, E. G., Comparison of Simulated Microgravity and Hydrostatic Pressure for Chondrogenesis of hASC. *Aerosp Med Hum Perform* **2017,** 88, (4), 377-384.

60. Luna, C.; Yew, A. G.; Hsieh, A. H., Effects of angular frequency during clinorotation on mesenchymal stem cell morphology and migration. *NPJ Microgravity* **2015,** 1, 15007.

61. Kraus, A.; Luetzenberg, R.; Abuagela, N.; Hollenberg, S.; Infanger, M., Spheroid formation and modulation of tenocyte-specific gene expression under simulated microgravity. *Muscles Ligaments Tendons J* **2017,** 7, (3), 411-417.

62. Ebnerasuly, F.; Hajebrahimi, Z.; Tabaie, S. M.; Darbouy, M., Effect of Simulated Microgravity Conditions on Differentiation of Adipose Derived Stem Cells towards Fibroblasts Using Connective Tissue Growth Factor. *Iran J Biotechnol* **2017,** 15, (4), 241-251.

63. Kopp, S.; Kruger, M.; Feldmann, S.; Oltmann, H.; Schutte, A.; Schmitz, B.; Bauer, J.; Schulz, H.; Saar, K.; Huebner, N.; Wehland, M.; Nassef, M. Z.; Melnik, D.; Meltendorf, S.; Infanger, M.; Grimm, D., Thyroid cancer cells in space during the TEXUS-53 sounding rocket mission - The THYROID Project. *Sci Rep* **2018,** 8, (1), 10355.

64. Zhao, T.; Li, R.; Tan, X.; Zhang, J.; Fan, C.; Zhao, Q.; Deng, Y.; Xu, A.; Lukong, K. E.; Genth, H.; Xiang, J., Simulated Microgravity Reduces Focal Adhesions and Alters Cytoskeleton and Nuclear Positioning Leading to Enhanced Apoptosis via Suppressing FAK/RhoA-Mediated mTORC1/NF-kappaB and ERK1/2 Pathways. *Int J Mol Sci* **2018,** 19, (7).

65. Li, N.; Wang, C.; Sun, S.; Zhang, C.; Lu, D.; Chen, Q.; Long, M., Microgravity-Induced Alterations of Inflammation-Related Mechanotransduction in Endothelial Cells on Board SJ-10 Satellite. *Front Physiol* **2018,** 9, 1025.

66. Lu, D.; Sun, S.; Zhang, F.; Luo, C.; Zheng, L.; Wu, Y.; Li, N.; Zhang, C.; Wang, C.; Chen, Q.; Long, M., Microgravity-induced hepatogenic differentiation of rBMSCs on board the SJ-10 satellite. *FASEB J* **2019,** 33, (3), 4273-4286.

67. Thiel, C. S.; Tauber, S.; Lauber, B.; Polzer, J.; Seebacher, C.; Uhl, R.; Neelam, S.; Zhang, Y.; Levine, H.; Ullrich, O., Rapid Morphological and Cytoskeletal Response to Microgravity in Human Primary Macrophages. *Int J Mol Sci* **2019,** 20, (10).

68. Koaykul, C.; Kim, M. H.; Kawahara, Y.; Yuge, L.; Kino-Oka, M., Alterations in Nuclear Lamina and the Cytoskeleton of Bone Marrow-Derived Human Mesenchymal Stem Cells Cultured Under Simulated Microgravity Conditions. *Stem Cells Dev* **2019,** 28, (17), 1167-1176.

69. Kashirina, D. N.; Kononikhin, A. S.; Larina, I. M.; Buravkova, L. B., Secretome of Cultured Human Endothelial Cells in Simulated Microgravity. *Bull Exp Biol Med* **2019,** 167, (1), 35-38.

70. Nassef, M. Z.; Kopp, S.; Wehland, M.; Melnik, D.; Sahana, J.; Kruger, M.; Corydon, T. J.; Oltmann, H.; Schmitz, B.; Schutte, A.; Bauer, T. J.; Infanger, M.; Grimm, D., Real Microgravity Influences the Cytoskeleton and Focal Adhesions in Human Breast Cancer Cells. *Int J Mol Sci* **2019,** 20, (13).

71. Buken, C.; Sahana, J.; Corydon, T. J.; Melnik, D.; Bauer, J.; Wehland, M.; Kruger, M.; Balk, S.; Abuagela, N.; Infanger, M.; Grimm, D., Morphological and Molecular Changes in Juvenile Normal Human Fibroblasts Exposed to Simulated Microgravity. *Sci Rep* **2019,** 9, (1), 11882.

72. Ebnerasuly, F.; Hajebrahimi, Z.; Tabaie, S. M.; Darbouy, M., Simulated Microgravity Condition Alters the Gene Expression of some ECM and Adhesion Molecules in Adipose Derived Stem Cells. *Int J Mol Cell Med* **2018,** 7, (3), 146-157.

73. Koaykul, C.; Kim, M. H.; Kawahara, Y.; Yuge, L.; Kino-Oka, M., Maintenance of Neurogenic Differentiation Potential in Passaged Bone Marrow-Derived Human Mesenchymal Stem Cells Under Simulated Microgravity Conditions. *Stem Cells Dev* **2019,** 28, (23), 1552-1561.

74. Strube, F.; Infanger, M.; Wehland, M.; Delvinioti, X.; Romswinkel, A.; Dietz, C.; Kraus, A., Alteration of Cytoskeleton Morphology and Gene Expression in Human Breast Cancer Cells under Simulated Microgravity. *Cell J* **2020,** 22, (1), 106-114.

75. Shi, W.; Zhang, Y.; Chen, K.; He, J.; Feng, X.; Wei, W.; Hua, J.; Wang, J., Primary cilia act as microgravity sensors by depolymerizing microtubules to inhibit osteoblastic differentiation and mineralization. *Bone* **2020,** 136, 115346.

76. Quynh Chi, H. N.; Nghia Son, H.; Chinh Chung, D.; Huan, L. D.; Hong Diem, T.; Long, L. T., Simulated microgravity reduces proliferation and reorganizes the cytoskeleton of human umbilical cord mesenchymal stem cells. *Physiol Res* **2020,** 69, (5), 897-906.

77. Ho, C. N. Q.; Tran, M. T.; Doan, C. C.; Hoang, S. N.; Tran, D. H.; Le, L. T., Simulated Microgravity Inhibits the Proliferation of Chang Liver Cells by Attenuation of the Major Cell Cycle Regulators and Cytoskeletal Proteins. *Int J Mol Sci* **2021,** 22, (9).

78. Cialdai, F.; Bolognini, D.; Vignali, L.; Iannotti, N.; Cacchione, S.; Magi, A.; Balsamo, M.; Vukich, M.; Neri, G.; Donati, A.; Monici, M.; Capaccioli, S.; Lulli, M., Effect of space flight on the behavior of human retinal pigment epithelial ARPE-19 cells and evaluation of coenzyme Q10 treatment. *Cell Mol Life Sci* **2021,** 78, (23), 7795-7812.

79. Grimm, D.; Schulz, H.; Kruger, M.; Cortes-Sanchez, J. L.; Egli, M.; Kraus, A.; Sahana, J.; Corydon, T. J.; Hemmersbach, R.; Wise, P. M.; Infanger, M.; Wehland, M., The Fight against Cancer by Microgravity: The Multicellular Spheroid as a Metastasis Model. *Int J Mol Sci* **2022,** 23, (6).

80. Schulz, H.; Dietrichs, D.; Wehland, M.; Corydon, T. J.; Hemmersbach, R.; Liemersdorf, C.; Melnik, D.; Hubner, N.; Saar, K.; Infanger, M.; Grimm, D., In Prostate Cancer Cells Cytokines Are Early Responders to Gravitational Changes Occurring in Parabolic Flights. *Int J Mol Sci* **2022,** 23, (14).

81. Wang, J.; Zhen, Z.; Wang, Y.; Wu, R.; Hu, Y.; Fu, Q.; Li, Y.; Xin, B.; Song, J.; Li, J.; Ren, Y.; Feng, L.; Cheng, H.; Wang, A.; Hu, L.; Ling, S.; Li, Y., Non-Invasive Skin Imaging Assessment of Human Stress During Head-Down Bed Rest Using a Portable Handheld Two-Photon Microscope. *Front Physiol* **2022,** 13, 899830.

82. Zhou, Y.; Lv, W.; Peng, X.; Cheng, Y.; Tu, Y.; Song, G.; Luo, Q., Simulated microgravity attenuates skin wound healing by inhibiting dermal fibroblast migration via F-actin/YAP signaling pathway. *J Cell Physiol* **2023,** 238, (12), 2751-2764.

83. Garbacki, N.; Willems, J.; Neutelings, T.; Lambert, C.; Deroanne, C.; Adrian, A.; Franz, M.; Maurer, M.; De Gieter, P.; Nusgens, B.; Colige, A., Microgravity triggers ferroptosis and accelerates senescence in the MG-63 cell model of osteoblastic cells. *NPJ Microgravity* **2023,** 9, (1), 91.

84. Scotti, M. M.; Wilson, B. K.; Bubenik, J. L.; Yu, F.; Swanson, M. S.; Allen, J. B., Spaceflight effects on human vascular smooth muscle cell phenotype and function. *NPJ Microgravity* **2024,** 10, (1), 41.

85. Grimm, D.; Corydon, T. J.; Sahana, J.; Gonzalez-Torres, L. F.; Kraus, A.; Marchal, S.; Wise, P. M.; Simonsen, U.; Kruger, M., Recent studies of the effects of microgravity on cancer cells and the development of 3D multicellular cancer spheroids. *Stem Cells Transl Med* **2025,** 14, (3).

86. Doty, S. B.; Morey-Holton, E. R.; Durnova, G. N.; Kaplansky, A. S., Cosmos 1887: morphology, histochemistry, and vasculature of the growing rat tibia. *FASEB J* **1990,** 4, (1), 16-23.

87. Riley, D. A.; Ellis, S.; Slocum, G. R.; Satyanarayana, T.; Bain, J. L.; Sedlak, F. R., Hypogravity-induced atrophy of rat soleus and extensor digitorum longus muscles. *Muscle Nerve* **1987,** 10, (6), 560-8.

88. Ross, M. D., Changes in ribbon synapses and rough endoplasmic reticulum of rat utricular macular hair cells in weightlessness. *Acta Otolaryngol* **2000,** 120, (4), 490-9.

89. Savary, C. A.; Grazziuti, M. L.; Przepiorka, D.; Tomasovic, S. P.; McIntyre, B. W.; Woodside, D. G.; Pellis, N. R.; Pierson, D. L.; Rex, J. H., Characteristics of human dendritic cells generated in a microgravity analog culture system. *In Vitro Cell Dev Biol Anim* **2001,** 37, (4), 216-22.

90. Smolen, J. E.; Fossett, M. C.; Joe, Y.; Prince, J. E.; Priest, E.; Kanwar, S.; Smith, C. W., Antiorthostatic suspension for 14 days does not diminish the oxidative response of neutrophils in mice. *Aviat Space Environ Med* **2000,** 71, (12), 1239-47.

91. Garcia-Ovejero, D.; Trejo, J. L.; Ciriza, I.; Walton, K. D.; Garcia-Segura, L. M., Space flight affects magnocellular supraoptic neurons of young prepuberal rats: transient and permanent effects. *Brain Res Dev Brain Res* **2001,** 130, (2), 191-205.

92. Kaur, I.; Simons, E. R.; Castro, V. A.; Mark Ott, C.; Pierson, D. L., Changes in neutrophil functions in astronauts. *Brain Behav Immun* **2004,** 18, (5), 443-50.

93. Kaur, I.; Simons, E. R.; Castro, V. A.; Ott, C. M.; Pierson, D. L., Changes in monocyte functions of astronauts. *Brain Behav Immun* **2005,** 19, (6), 547-54.

94. Vincent, L.; Avancena, P.; Cheng, J.; Rafii, S.; Rabbany, S. Y., Simulated microgravity impairs leukemic cell survival through altering VEGFR-2/VEGF-A signaling pathway. *Ann Biomed Eng* **2005,** 33, (10), 1405-10.

95. Ferreira, R.; Neuparth, M. J.; Vitorino, R.; Appell, H. J.; Amado, F.; Duarte, J. A., Evidences of apoptosis during the early phases of soleus muscle atrophy in hindlimb suspended mice. *Physiol Res* **2008,** 57, (4), 601-611.

96. Kaufmann, I.; Schachtner, T.; Feuerecker, M.; Schelling, G.; Thiel, M.; Chouker, A., Parabolic flight primes cytotoxic capabilities of polymorphonuclear leucocytes in humans. *Eur J Clin Invest* **2009,** 39, (8), 723-8.

97. Talbot, N. C.; Caperna, T. J.; Blomberg, L.; Graninger, P. G.; Stodieck, L. S., The effects of space flight and microgravity on the growth and differentiation of PICM-19 pig liver stem cells. *In Vitro Cell Dev Biol Anim* **2010,** 46, (6), 502-15.

98. Puca, A.; Russo, G.; Giordano, A., Properties of mechano-transduction via simulated microgravity and its effects on intracellular trafficking of VEGFR's. *Oncotarget* **2012,** 3, (4), 426-34.

99. Versari, S.; Longinotti, G.; Barenghi, L.; Maier, J. A.; Bradamante, S., The challenging environment on board the International Space Station affects endothelial cell function by triggering oxidative stress through thioredoxin interacting protein overexpression: the ESA-SPHINX experiment. *FASEB J* **2013,** 27, (11), 4466-75.

100. Wang, Y. C.; Lu, D. Y.; Shi, F.; Zhang, S.; Yang, C. B.; Wang, B.; Cao, X. S.; Du, T. Y.; Gao, Y.; Zhao, J. D.; Sun, X. Q., Clinorotation enhances autophagy in vascular endothelial cells. *Biochem Cell Biol* **2013,** 91, (5), 309-14.

101. Adrian, A.; Schoppmann, K.; Sromicki, J.; Brungs, S.; von der Wiesche, M.; Hock, B.; Kolanus, W.; Hemmersbach, R.; Ullrich, O., The oxidative burst reaction in mammalian cells depends on gravity. *Cell Commun Signal* **2013,** 11, 98.

102. Hwang, S. A.; Crucian, B.; Sams, C.; Actor, J. K., Post-Spaceflight (STS-135) Mouse Splenocytes Demonstrate Altered Activation Properties and Surface Molecule Expression. *PLoS One* **2015,** 10, (5), e0124380.

103. Wang, Y.; Iqbal, J.; Liu, Y.; Su, R.; Lu, S.; Peng, G.; Zhang, Y.; Qing, H.; Deng, Y., Effects of simulated microgravity on the expression of presynaptic proteins distorting the GABA/glutamate equilibrium--A proteomics approach. *Proteomics* **2015,** 15, (22), 3883-91.

104. Xu, H.; Liu, R.; Ning, D.; Zhang, J.; Yang, R.; Riquelme, M. A.; Li, J.; Jiang, J. X.; Shang, P., Biological responses of osteocytic connexin 43 hemichannels to simulated microgravity. *J Orthop Res* **2017,** 35, (6), 1195-1202.

105. Yoo, Y. M.; Han, T. Y.; Kim, H. S., Melatonin Suppresses Autophagy Induced by Clinostat in Preosteoblast MC3T3-E1 Cells. *Int J Mol Sci* **2016,** 17, (4), 526.

106. Pecaut, M. J.; Mao, X. W.; Bellinger, D. L.; Jonscher, K. R.; Stodieck, L. S.; Ferguson, V. L.; Bateman, T. A.; Mohney, R. P.; Gridley, D. S., Is spaceflight-induced immune dysfunction linked to systemic changes in metabolism? *PLoS One* **2017,** 12, (5), e0174174.

107. Ethiraj, P.; Link, J. R.; Sinkway, J. M.; Brown, G. D.; Parler, W. A.; Reddy, S. V., Microgravity modulation of syncytin-A expression enhance osteoclast formation. *J Cell Biochem* **2018,** 119, (7), 5696-5703.

108. Bauer, J.; Wehland, M.; Infanger, M.; Grimm, D.; Gombocz, E., Semantic Analysis of Posttranslational Modification of Proteins Accumulated in Thyroid Cancer Cells Exposed to Simulated Microgravity. *Int J Mol Sci* **2018,** 19, (8).

109. Li, C. F.; Pan, Y. K.; Gao, Y.; Shi, F.; Wang, Y. C.; Sun, X. Q., Autophagy protects HUVECs against ER stress-mediated apoptosis under simulated microgravity. *Apoptosis* **2019,** 24, (9-10), 812-825.

110. Ethiraj, P.; Ottinger, A. M.; Singh, T.; Singh, A.; Haire, K. M.; Reddy, S. V., Proteasome inhibition suppress microgravity elevated RANK signaling during osteoclast differentiation. *Cytokine* **2020,** 125, 154821.

111. Bauer, T. J.; Gombocz, E.; Wehland, M.; Bauer, J.; Infanger, M.; Grimm, D., Insight in Adhesion Protein Sialylation and Microgravity Dependent Cell Adhesion-An Omics Network Approach. *Int J Mol Sci* **2020,** 21, (5).

112. Jiang, M.; Wang, H.; Liu, Z.; Lin, L.; Wang, L.; Xie, M.; Li, D.; Zhang, J.; Zhang, R., Endoplasmic reticulum stress-dependent activation of iNOS/NO-NF-kappaB signaling and NLRP3 inflammasome contributes to endothelial inflammation and apoptosis associated with microgravity. *FASEB J* **2020,** 34, (8), 10835-10849.

113. Berezovskaya, A. S.; Tyganov, S. A.; Nikolaeva, S. D.; Naumova, A. A.; Merkulyeva, N. S.; Shenkman, B. S.; Glazova, M. V., Dynamic Foot Stimulations During Short-Term Hindlimb Unloading Prevent Dysregulation of the Neurotransmission in the Hippocampus of Rats. *Cell Mol Neurobiol* **2021,** 41, (7), 1549-1561.

114. Chen, Y.; Xue, F.; Russo, A.; Wan, Y., Proteomic Analysis of Extracellular Vesicles Derived from MDA-MB-231 Cells in Microgravity. *Protein J* **2021,** 40, (1), 108-118.

115. Wise, P. M.; Neviani, P.; Riwaldt, S.; Corydon, T. J.; Wehland, M.; Braun, M.; Kruger, M.; Infanger, M.; Grimm, D., Changes in Exosome Release in Thyroid Cancer Cells after Prolonged Exposure to Real Microgravity in Space. *Int J Mol Sci* **2021,** 22, (4).

116. Bisserier, M.; Brojakowska, A.; Saffran, N.; Rai, A. K.; Lee, B.; Coleman, M.; Sebastian, A.; Evans, A.; Mills, P. J.; Addya, S.; Arakelyan, A.; Garikipati, V. N. S.; Hadri, L.; Goukassian, D. A., Astronauts Plasma-Derived Exosomes Induced Aberrant EZH2-Mediated H3K27me3 Epigenetic Regulation of the Vitamin D Receptor. *Front Cardiovasc Med* **2022,** 9, 855181.

117. Wise, P. M.; Sahana, J.; Neviani, P.; Corydon, T. J.; Schulz, H.; Wehland, M.; Infanger, M.; Grimm, D., Prolonged Exposure to Simulated Microgravity Changes Release of Small Extracellular Vesicle in Breast Cancer Cells. *Int J Mol Sci* **2022,** 23, (24).

118. Tran, V.; Carpo, N.; Cepeda, C.; Espinosa-Jeffrey, A., Oligodendrocyte Progenitors Display Enhanced Proliferation and Autophagy after Space Flight. *Biomolecules* **2023,** 13, (2).

119. Ranade, A.; Khan, A. A.; Gul, M. T.; Suresh, S.; Qaisar, R.; Ahmad, F.; Karim, A., Suppression of endoplasmic reticulum stress reverses hindlimb unloading-induced hepatic cellular processes in mice. *Biochim Biophys Acta Gen Subj* **2023,** 1867, (9), 130422.

120. Carpo, N.; Tran, V.; Biancotti, J. C.; Cepeda, C.; Espinosa-Jeffrey, A., Space Flight Enhances Stress Pathways in Human Neural Stem Cells. *Biomolecules* **2024,** 14, (1).

121. Ohira, T.; Ino, Y.; Kawao, N.; Mizukami, Y.; Okada, K.; Matsuo, O.; Hirano, H.; Kimura, Y.; Kaji, H., Plasminogen deficiency exacerbates skeletal muscle loss during mechanical unloading in developing mice. *J Appl Physiol (1985)* **2024,** 136, (3), 643-658.

122. Houerbi, N.; Kim, J.; Overbey, E. G.; Batra, R.; Schweickart, A.; Patras, L.; Lucotti, S.; Ryon, K. A.; Najjar, D.; Meydan, C.; Damle, N.; Chin, C.; Narayanan, S. A.; Guarnieri, J. W.; Widjaja, G.; Beheshti, A.; Tobias, G.; Vatter, F.; Hirschberg, J. W.; Kleinman, A.; Afshin, E. E.; MacKay, M.; Chen, Q.; Miller, D.; Gajadhar, A. S.; Williamson, L.; Tandel, P.; Yang, Q.; Chu, J.; Benz, R.; Siddiqui, A.; Hornburg, D.; Gross, S.; Shirah, B.; Krumsiek, J.; Mateus, J.; Mao, X.; Matei, I.; Mason, C. E., Secretome profiling reveals acute changes in oxidative stress, brain homeostasis, and coagulation following short-duration spaceflight. *Nat Commun* **2024,** 15, (1), 4862.

123. Overbey, E. G.; Ryon, K.; Kim, J.; Tierney, B. T.; Klotz, R.; Ortiz, V.; Mullane, S.; Schmidt, J. C.; MacKay, M.; Damle, N.; Najjar, D.; Matei, I.; Patras, L.; Garcia Medina, J. S.; Kleinman, A. S.; Wain Hirschberg, J.; Proszynski, J.; Narayanan, S. A.; Schmidt, C. M.; Afshin, E. E.; Innes, L.; Saldarriaga, M. M.; Schmidt, M. A.; Granstein, R. D.; Shirah, B.; Yu, M.; Lyden, D.; Mateus, J.; Mason, C. E., Collection of biospecimens from the inspiration4 mission establishes the standards for the space omics and medical atlas (SOMA). *Nat Commun* **2024,** 15, (1), 4964.

124. Ouchi, T.; Kono, K.; Satou, R.; Kurashima, R.; Yamaguchi, K.; Kimura, M.; Shibukawa, Y., Upregulation of Amy1 in the salivary glands of mice exposed to a lunar gravity environment using the multiple artificial gravity research system. *Front Physiol* **2024,** 15, 1417719.

125. Oommen, A. M.; Stafford, P.; Joshi, L., Profiling muscle transcriptome in mice exposed to microgravity using gene set enrichment analysis. *NPJ Microgravity* **2024,** 10, (1), 94.

126. Velarde, M.; Sempore, M. Y.; Allibert, V.; Montel, V.; Castells, J.; Treffel, L.; Chopard, A.; Brioche, T.; Cochon, L.; Morel, J.; Bastide, B.; Durieux, A. C.; Stevens, L.; Freyssenet, D., Molecular determinants of skeletal muscle force loss in response to 5 days of dry immersion in human. *J Cachexia Sarcopenia Muscle* **2024,** 15, (6), 2323-2337.

127. Li, L. F.; Yu, J.; Li, R.; Li, S. S.; Huang, J. Y.; Wang, M. D.; Jiang, L. N.; Xu, J. H.; Wang, Z., Apoptosis, Mitochondrial Autophagy, Fission, and Fusion Maintain Mitochondrial Homeostasis in Mouse Liver Under Tail Suspension Conditions. *Int J Mol Sci* **2024,** 25, (20).

128. Chang, M.; Liu, R.; Chen, B.; Xu, J.; Wang, W.; Ji, Y.; Gao, Z.; Liu, B.; Yao, X.; Sun, H.; Xu, F.; Shen, Y., hBMSC-EVs alleviate weightlessness-induced skeletal muscle atrophy by suppressing oxidative stress and inflammation. *Stem Cell Res Ther* **2025,** 16, (1), 46.

129. Fan, H.; Lu, D.; Lu, Z.; Li, H.; Qi, Z.; Sun, S.; Guan, D.; Long, M.; Gao, M.; Liu, S., TRPML1 ion channel promotes HepaRG cell differentiation under simulated microgravity conditions. *NPJ Microgravity* **2025,** 11, (1), 9.

130. Schmitt, D. A.; Malouvier, A.; Holy, X.; Marie, P. J.; Caissard, J. C.; Zerath, E., Receptor-ligand binding on osteoblasts in microgravity obtained by parabolic flight. *Physiologist* **1991,** 34, (1 Suppl), S64-5.

131. Mednieks, M. I.; Hand, A. R., Salivary gland ultrastructure and cyclic AMP-dependent reactions in Spacelab 3 rats. *Am J Physiol* **1987,** 252, (2 Pt 2), R233-9.

132. Schmitt, D. A.; Ohlmann, P.; Gachet, C.; Cazenave, J. P., Platelet shape change and protein phosphorylation induced by ADP and thrombin are not sensitive to short periods of microgravity. *J Cell Sci* **1993,** 104 (Pt 3), 805-10.

133. Rijken, P. J.; de Groot, R. P.; van Belzen, N.; de Laat, S. W.; Boonstra, J.; Verkleij, A. J., Inhibition of EGF-induced signal transduction by microgravity is independent of EGF receptor redistribution in the plasma membrane of human A431 cells. *Exp Cell Res* **1993,** 204, (2), 373-7.

134. Cooper, D.; Pellis, N. R., Suppressed PHA activation of T lymphocytes in simulated microgravity is restored by direct activation of protein kinase C. *J Leukoc Biol* **1998,** 63, (5), 550-62.

135. Hatton, J. P.; Gaubert, F.; Lewis, M. L.; Darsel, Y.; Ohlmann, P.; Cazenave, J. P.; Schmitt, D., The kinetics of translocation and cellular quantity of protein kinase C in human leukocytes are modified during spaceflight. *FASEB J* **1999,** 13 Suppl, S23-33.

136. Hashemi, B. B.; Penkala, J. E.; Vens, C.; Huls, H.; Cubbage, M.; Sams, C. F., T cell activation responses are differentially regulated during clinorotation and in spaceflight. *FASEB J* **1999,** 13, (14), 2071-82.

137. Akiyama, H.; Kanai, S.; Hirano, M.; Shimokawa, H.; Katano, H.; Mukai, C.; Nagaoka, S.; Morita, S.; Kumei, Y., Expression of PDGF-beta receptor, EGF receptor, and receptor adaptor protein Shc in rat osteoblasts during spaceflight. *Mol Cell Biochem* **1999,** 202, (1-2), 63-71.

138. Felix, J. A.; Dirksen, E. R.; Woodruff, M. L., Selected contribution: PKC activation inhibits Ca(2+) signaling in tracheal epithelial cells kept in simulated microgravity. *J Appl Physiol (1985)* **2000,** 89, (2), 855-64; discussion 848.

139. Jessup, J. M.; Frantz, M.; Sonmez-Alpan, E.; Locker, J.; Skena, K.; Waller, H.; Battle, P.; Nachman, A.; Bhatti; Weber, M. E.; Thomas, D. A.; Curbeam, R. L., Jr.; Baker, T. L.; Goodwin, T. J., Microgravity culture reduces apoptosis and increases the differentiation of a human colorectal carcinoma cell line. *In Vitro Cell Dev Biol Anim* **2000,** 36, (6), 367-73.

140. Sato, A.; Hamazaki, T.; Oomura, T.; Osada, H.; Kakeya, M.; Watanabe, M.; Nakamura, T.; Nakamura, Y.; Koshikawa, N.; Yoshizaki, I.; Aizawa, S.; Yoda, S.; Ogiso, A.; Takaoki, M.; Kohno, Y.; Tanaka, H., Effects of microgravity on c-fos gene expression in osteoblast-like MC3T3-E1 cells. *Adv Space Res* **1999,** 24, (6), 807-13.

141. Sundaresan, A.; Risin, D.; Pellis, N. R., Loss of signal transduction and inhibition of lymphocyte locomotion in a ground-based model of microgravity. *In Vitro Cell Dev Biol Anim* **2002,** 38, (2), 118-22.

142. Hatton, J. P.; Gaubert, F.; Cazenave, J. P.; Schmitt, D., Microgravity modifies protein kinase C isoform translocation in the human monocytic cell line U937 and human peripheral blood T-cells. *J Cell Biochem* **2002,** 87, (1), 39-50.

143. Yuge, L.; Hide, I.; Kumagai, T.; Kumei, Y.; Takeda, S.; Kanno, M.; Sugiyama, M.; Kataoka, K., Cell differentiation and p38(MAPK) cascade are inhibited in human osteoblasts cultured in a three-dimensional clinostat. *In Vitro Cell Dev Biol Anim* **2003,** 39, (1-2), 89-97.

144. Sundaresan, A.; Risin, D.; Pellis, N. R., Modeled microgravity-induced protein kinase C isoform expression in human lymphocytes. *J Appl Physiol (1985)* **2004,** 96, (6), 2028-33.

145. Bucaro, M. A.; Fertala, J.; Adams, C. S.; Steinbeck, M.; Ayyaswamy, P.; Mukundakrishnan, K.; Shapiro, I. M.; Risbud, M. V., Bone cell survival in microgravity: evidence that modeled microgravity increases osteoblast sensitivity to apoptogens. *Ann N Y Acad Sci* **2004,** 1027, 64-73.

146. Meyers, V. E.; Zayzafoon, M.; Gonda, S. R.; Gathings, W. E.; McDonald, J. M., Modeled microgravity disrupts collagen I/integrin signaling during osteoblastic differentiation of human mesenchymal stem cells. *J Cell Biochem* **2004,** 93, (4), 697-707.

147. Boonyaratanakornkit, J. B.; Cogoli, A.; Li, C. F.; Schopper, T.; Pippia, P.; Galleri, G.; Meloni, M. A.; Hughes-Fulford, M., Key gravity-sensitive signaling pathways drive T cell activation. *FASEB J* **2005,** 19, (14), 2020-2.

148. Kumei, Y.; Shimokawa, H.; Ohya, K.; Katano, H.; Akiyama, H.; Hirano, M.; Morita, S., Small GTPase Ras and Rho expression in rat osteoblasts during spaceflight. *Ann N Y Acad Sci* **2007,** 1095, 292-9.

149. Yin, W.; Liu, J. C.; Fan, R.; Sun, X. Q.; Ma, J.; Feng, N.; Zhang, Q. Y.; Yin, Z.; Zhang, S. M.; Guo, H. T.; Bi, H.; Wang, Y. M.; Sun, X.; Cheng, L.; Cui, Q.; Yu, S. Q.; Yi, D. H.; Pei, J. M., Modulation of beta-adrenoceptor signaling in the hearts of 4-wk simulated weightlessness rats. *J Appl Physiol (1985)* **2008,** 105, (2), 569-74.

150. Li, X.; Liu, C. T.; Zhou, H., The influence of leptin on the activity of lung lymphocytes under simulated microgravity. *Eur J Appl Physiol* **2009,** 107, (3), 335-44.

151. Kang, C. Y.; Zou, L.; Yuan, M.; Wang, Y.; Li, T. Z.; Zhang, Y.; Wang, J. F.; Li, Y.; Deng, X. W.; Liu, C. T., Impact of simulated microgravity on microvascular endothelial cell apoptosis. *Eur J Appl Physiol* **2011,** 111, (9), 2131-8.

152. Yu, B.; Yu, D.; Cao, L.; Zhao, X.; Long, T.; Liu, G.; Tang, T.; Zhu, Z., Simulated microgravity using a rotary cell culture system promotes chondrogenesis of human adipose-derived mesenchymal stem cells via the p38 MAPK pathway. *Biochem Biophys Res Commun* **2011,** 414, (2), 412-8.

153. Lawler, J. M.; Kwak, H. B.; Kim, J. H.; Lee, Y.; Hord, J. M.; Martinez, D. A., Biphasic stress response in the soleus during reloading after hind limb unloading. *Med Sci Sports Exerc* **2012,** 44, (4), 600-9.

154. Chiang, M. C.; Lin, H.; Cheng, Y. C.; Yen, C. H.; Huang, R. N.; Lin, K. H., beta-adrenoceptor pathway enhances mitochondrial function in human neural stem cells via rotary cell culture system. *J Neurosci Methods* **2012,** 207, (2), 130-6.

155. Shi, F.; Wang, Y. C.; Zhao, T. Z.; Zhang, S.; Du, T. Y.; Yang, C. B.; Li, Y. H.; Sun, X. Q., Effects of simulated microgravity on human umbilical vein endothelial cell angiogenesis and role of the PI3K-Akt-eNOS signal pathway. *PLoS One* **2012,** 7, (7), e40365.

156. Bai, Y.; Tsunematsu, T.; Jiao, Q.; Ohnuki, Y.; Mototani, Y.; Shiozawa, K.; Jin, M.; Cai, W.; Jin, H. L.; Fujita, T.; Ichikawa, Y.; Suita, K.; Kurotani, R.; Yokoyama, U.; Sato, M.; Iwatsubo, K.; Ishikawa, Y.; Okumura, S., Pharmacological stimulation of type 5 adenylyl cyclase stabilizes heart rate under both microgravity and hypergravity induced by parabolic flight. *J Pharmacol Sci* **2012,** 119, (4), 381-9.

157. Muid, S.; Froemming, G. R.; Ali, A. M.; Nawawi, H., Interleukin-6 and intercellular cell adhesion molecule-1 expression remains elevated in revived live endothelial cells following spaceflight. *Malays J Pathol* **2013,** 35, (2), 165-76.

158. Mao, X. W.; Pecaut, M. J.; Stodieck, L. S.; Ferguson, V. L.; Bateman, T. A.; Bouxsein, M. L.; Gridley, D. S., Biological and metabolic response in STS-135 space-flown mouse skin. *Free Radic Res* **2014,** 48, (8), 890-7.

159. Masiello, M. G.; Cucina, A.; Proietti, S.; Palombo, A.; Coluccia, P.; D'Anselmi, F.; Dinicola, S.; Pasqualato, A.; Morini, V.; Bizzarri, M., Phenotypic switch induced by simulated microgravity on MDA-MB-231 breast cancer cells. *Biomed Res Int* **2014,** 2014, 652434.

160. Philippou, A.; Minozzo, F. C.; Spinazzola, J. M.; Smith, L. R.; Lei, H.; Rassier, D. E.; Barton, E. R., Masticatory muscles of mouse do not undergo atrophy in space. *FASEB J* **2015,** 29, (7), 2769-79.

161. Wang, C.; Chen, H.; Luo, H.; Zhu, L.; Zhao, Y.; Tian, H.; Wang, R.; Shang, P.; Zhao, Y., Microgravity activates p38 MAPK-C/EBPbeta pathway to regulate the expression of arginase and inflammatory cytokines in macrophages. *Inflamm Res* **2015,** 64, (5), 303-11.

162. Fuentes, T. I.; Appleby, N.; Raya, M.; Bailey, L.; Hasaniya, N.; Stodieck, L.; Kearns-Jonker, M., Simulated Microgravity Exerts an Age-Dependent Effect on the Differentiation of Cardiovascular Progenitors Isolated from the Human Heart. *PLoS One* **2015,** 10, (7), e0132378.

163. Kopp, S.; Warnke, E.; Wehland, M.; Aleshcheva, G.; Magnusson, N. E.; Hemmersbach, R.; Corydon, T. J.; Bauer, J.; Infanger, M.; Grimm, D., Mechanisms of three-dimensional growth of thyroid cells during long-term simulated microgravity. *Sci Rep* **2015,** 5, 16691.

164. Fix, D. K.; Hardee, J. P.; Bateman, T. A.; Carson, J. A., Effect of irradiation on Akt signaling in atrophying skeletal muscle. *J Appl Physiol (1985)* **2016,** 121, (4), 917-924.

165. Wu, X.; Li, D.; Liu, J.; Diao, L.; Ling, S.; Li, Y.; Gao, J.; Fan, Q.; Sun, W.; Li, Q.; Zhao, D.; Zhong, G.; Cao, D.; Liu, M.; Wang, J.; Zhao, S.; Liu, Y.; Bai, G.; Shi, H.; Xu, Z.; Wang, J.; Xue, C.; Jin, X.; Yuan, X.; Li, H.; Liu, C.; Sun, H.; Li, J.; Li, Y.; Li, Y., Dammarane Sapogenins Ameliorates Neurocognitive Functional Impairment Induced by Simulated Long-Duration Spaceflight. *Front Pharmacol* **2017,** 8, 315.

166. Arun, R. P.; Sivanesan, D.; Vidyasekar, P.; Verma, R. S., PTEN/FOXO3/AKT pathway regulates cell death and mediates morphogenetic differentiation of Colorectal Cancer Cells under Simulated Microgravity. *Sci Rep* **2017,** 7, (1), 5952.

167. Baio, J.; Martinez, A. F.; Bailey, L.; Hasaniya, N.; Pecaut, M. J.; Kearns-Jonker, M., Spaceflight Activates Protein Kinase C Alpha Signaling and Modifies the Developmental Stage of Human Neonatal Cardiovascular Progenitor Cells. *Stem Cells Dev* **2018,** 27, (12), 805-818.

168. Zhang, C.; Li, L.; Jiang, Y.; Wang, C.; Geng, B.; Wang, Y.; Chen, J.; Liu, F.; Qiu, P.; Zhai, G.; Chen, P.; Quan, R.; Wang, J., Space microgravity drives transdifferentiation of human bone marrow-derived mesenchymal stem cells from osteogenesis to adipogenesis. *FASEB J* **2018,** 32, (8), 4444-4458.

169. Dagdeviren, D.; Beallias, J.; Khan, I.; Mednieks, M. I.; Hand, A. R., Response of the mouse sublingual gland to spaceflight. *Eur J Oral Sci* **2018,** 126, (5), 373-381.

170. Zhang, S.; Yuan, M.; Cheng, C.; Xia, D. H.; Wu, S. W., Chinese Herbal Medicine Effects on Muscle Atrophy Induced by Simulated Microgravity. *Aerosp Med Hum Perform* **2018,** 89, (10), 883-888.

171. Jeong, A. J.; Kim, Y. J.; Lim, M. H.; Lee, H.; Noh, K.; Kim, B. H.; Chung, J. W.; Cho, C. H.; Kim, S.; Ye, S. K., Microgravity induces autophagy via mitochondrial dysfunction in human Hodgkin's lymphoma cells. *Sci Rep* **2018,** 8, (1), 14646.

172. Li, W. Y.; Li, X. Y.; Tian, Y. H.; Chen, X. R.; Zhou, J.; Zhu, B. Y.; Xi, H. R.; Gao, Y. H.; Xian, C. J.; Chen, K. M., Pulsed electromagnetic fields prevented the decrease of bone formation in hindlimb-suspended rats by activating sAC/cAMP/PKA/CREB signaling pathway. *Bioelectromagnetics* **2018,** 39, (8), 569-584.

173. Dietz, C.; Infanger, M.; Romswinkel, A.; Strube, F.; Kraus, A., Apoptosis Induction and Alteration of Cell Adherence in Human Lung Cancer Cells under Simulated Microgravity. *Int J Mol Sci* **2019,** 20, (14).

174. Cho, H. J.; Baek, M. O.; Khaliq, S. A.; Chon, S. J.; Son, K. H.; Lee, S. H.; Yoon, M. S., Microgravity inhibits decidualization via decreasing Akt activity and FOXO3a expression in human endometrial stromal cells. *Sci Rep* **2019,** 9, (1), 12094.

175. Nassef, M. Z.; Kopp, S.; Melnik, D.; Corydon, T. J.; Sahana, J.; Kruger, M.; Wehland, M.; Bauer, T. J.; Liemersdorf, C.; Hemmersbach, R.; Infanger, M.; Grimm, D., Short-Term Microgravity Influences Cell Adhesion in Human Breast Cancer Cells. *Int J Mol Sci* **2019,** 20, (22).

176. Zhang, R.; Jiang, M.; Zhang, J.; Qiu, Y.; Li, D.; Li, S.; Liu, J.; Liu, C.; Fang, Z.; Cao, F., Regulation of the cerebrovascular smooth muscle cell phenotype by mitochondrial oxidative injury and endoplasmic reticulum stress in simulated microgravity rats via the PERK-eIF2alpha-ATF4-CHOP pathway. *Biochim Biophys Acta Mol Basis Dis* **2020,** 1866, (8), 165799.

177. Wang, J.; Han, C.; Lu, Z.; Ge, P.; Cui, Y.; Zhao, D.; Yang, X.; Wu, B.; Qiang, L.; Zhang, Y.; Chai, Q.; Lei, Z.; Li, L.; Hua Liu, C.; Zhang, L., Simulated microgravity suppresses MAPK pathway-mediated innate immune response to bacterial infection and induces gut microbiota dysbiosis. *FASEB J* **2020,** 34, (11), 14631-14644.

178. Liang, L.; Li, H.; Cao, T.; Qu, L.; Zhang, L.; Fan, G. C.; Greer, P. A.; Li, J.; Jones, D. L.; Peng, T., Calpain activation mediates microgravity-induced myocardial abnormalities in mice via p38 and ERK1/2 MAPK pathways. *J Biol Chem* **2020,** 295, (49), 16840-16851.

179. Kong, L.; Wang, Y.; Wang, H.; Pan, Q.; Zuo, R.; Bai, S.; Zhang, X.; Lee, W. Y.; Kang, Q.; Li, G., Conditioned media from endothelial progenitor cells cultured in simulated microgravity promote angiogenesis and bone fracture healing. *Stem Cell Res Ther* **2021,** 12, (1), 47.

180. Monti, N.; Masiello, M. G.; Proietti, S.; Catizone, A.; Ricci, G.; Harrath, A. H.; Alwasel, S. H.; Cucina, A.; Bizzarri, M., Survival Pathways Are Differently Affected by Microgravity in Normal and Cancerous Breast Cells. *Int J Mol Sci* **2021,** 22, (2).

181. Riwaldt, S.; Corydon, T. J.; Pantalone, D.; Sahana, J.; Wise, P.; Wehland, M.; Kruger, M.; Melnik, D.; Kopp, S.; Infanger, M.; Grimm, D., Role of Apoptosis in Wound Healing and Apoptosis Alterations in Microgravity. *Front Bioeng Biotechnol* **2021,** 9, 679650.

182. Singh, R.; Rajput, M.; Singh, R. P., Simulated microgravity triggers DNA damage and mitochondria-mediated apoptosis through ROS generation in human promyelocytic leukemic cells. *Mitochondrion* **2021,** 61, 114-124.

183. Cheng, Y.; Zhou, Y.; Lv, W.; Luo, Q.; Song, G., Simulated Microgravity Inhibits Rodent Dermal Fibroblastic Differentiation of Mesenchymal Stem Cells by Suppressing ERK/beta-Catenin Signaling Pathway. *Int J Mol Sci* **2021,** 22, (19).

184. Wu, X.; Yin, Y.; Liu, J.; Zhu, Y.; Fan, Q.; Zhao, S.; Wang, J.; Gao, J.; Liu, Y.; Jiao, L.; Li, Y., Baoyuan jieyu formula ameliorates depression-like behaviour in rats induced by simulated long-term spaceflight composite stress through regulating MAPK and BDNF pathways. *Life Sci Space Res (Amst)* **2021,** 31, 34-42.

185. Ma, C.; Xiong, Y.; Han, P.; Zhang, X.; Cao, Y.; Wang, B.; Zhao, H.; Duan, E.; Zhang, J. V.; Lei, X., Simulated Microgravity Potentiates Hematopoietic Differentiation of Human Pluripotent Stem Cells and Supports Formation of 3D Hematopoietic Cluster. *Front Cell Dev Biol* **2021,** 9, 797060.

186. Masini, M. A.; Bonetto, V.; Manfredi, M.; Pasto, A.; Barberis, E.; Timo, S.; Vanella, V. V.; Robotti, E.; Masetto, F.; Andreoli, F.; Fiore, A.; Tavella, S.; Sica, A.; Donadelli, M.; Marengo, E., Prolonged exposure to simulated microgravity promotes stemness impairing morphological, metabolic and migratory profile of pancreatic cancer cells: a comprehensive proteomic, lipidomic and transcriptomic analysis. *Cell Mol Life Sci* **2022,** 79, (5), 226.

187. Wen, J.; Xu, Z.; Ma, X.; Zhao, Y., Wound Healing Effects of Dracontomelon dao on Bacterial Infection Wounds in Rats and Its Potential Mechanisms under Simulated Space Environment. *Evid Based Complement Alternat Med* **2022,** 2022, 4593201.

188. Hanson, A. M.; Young, M. H.; Harrison, B. C.; Zhou, X.; Han, H. Q.; Stodieck, L. S.; Ferguson, V. L., Inhibiting myostatin signaling partially mitigates structural and functional adaptations to hindlimb suspension in mice. *NPJ Microgravity* **2023,** 9, (1), 2.

189. Sahana, J.; Cortes-Sanchez, J. L.; Sandt, V.; Melnik, D.; Corydon, T. J.; Schulz, H.; Cai, Z.; Evert, K.; Grimm, D.; Wehland, M., Long-Term Simulation of Microgravity Induces Changes in Gene Expression in Breast Cancer Cells. *Int J Mol Sci* **2023,** 24, (2).

190. Chen, F.; Jiang, N.; Zhang, Y. W.; Xie, M. Z.; Liu, X. M., Protective effect of Gastrodia elata blume ameliorates simulated weightlessness-induced cognitive impairment in mice. *Life Sci Space Res (Amst)* **2023,** 36, 1-7.

191. Qiu, M.; Xie, Y.; Tu, L.; Zhao, M.; Yang, M.; Fang, L.; Gu, J., Overexpressed RAD51 promoted osteogenic differentiation by activating IGF1R/PI3K/AKT pathway in osteoblasts. *Acta Biochim Pol* **2023,** 70, (1), 31-36.

192. Veliz, A. L.; Mamoun, L.; Hughes, L.; Vega, R.; Holmes, B.; Monteon, A.; Bray, J.; Pecaut, M. J.; Kearns-Jonker, M., Transcriptomic Effects on the Mouse Heart Following 30 Days on the International Space Station. *Biomolecules* **2023,** 13, (2).

193. Jiang, N.; Lv, J.; Zhang, Y.; Sun, X.; Yao, C.; Wang, Q.; He, Q.; Liu, X., Protective effects of ginsenosides Rg1 and Rb1 against cognitive impairment induced by simulated microgravity in rats. *Front Pharmacol* **2023,** 14, 1167398.

194. Zhang, Y.; Huang, H.; Yao, C.; Sun, X.; He, Q.; Choudharyc, M. I.; Chen, S.; Liu, X.; Jiang, N., Fresh Gastrodia elata Blume alleviates simulated weightlessness-induced cognitive impairment by regulating inflammatory and apoptosis-related pathways. *Front Pharmacol* **2023,** 14, 1173920.

195. Yue, Y.; Yang, J. Q.; Lu, S. Y.; Ge, J.; Nie, H. Y.; Liu, K. G.; Liu, F.; Li, H.; Yan, H. F.; Zhang, T.; Sun, P. M.; Sun, H. W.; Yang, J. W.; Zhou, J. L.; Cui, Y., Simulated microgravity altered the proliferation, apoptosis, and extracellular matrix formation of L929 fibroblasts and the transforming growth factor-beta1/Smad3 signaling pathway. *Skin Res Technol* **2023,** 29, (5), e13341.

196. Shi, Q.; Gui, J.; Sun, L.; Song, Y.; Na, J.; Zhang, J.; Fan, Y.; Zheng, L., Frizzled-9 triggers actin polymerization and activates mechano-transducer YAP to rescue simulated microgravity-induced osteoblast dysfunction. *FASEB J* **2023,** 37, (9), e23147.

197. Li, J.; Xue, C.; Yang, H.; Zhang, J.; Li, G.; Li, J.; Kuang, F.; Chen, J.; Zhang, S.; Gao, F.; Kou, Z.; Zhang, X.; Dong, L., Simulated weightlessness induces hippocampal insulin resistance and cognitive impairment. *Life Sci* **2023,** 333, 122112.

198. Zhang, J.; Wang, X.; Fu, Z.; Xing, C.; Wang, Z.; Yang, H.; Li, J.; Liu, M.; Dong, L.; Zhang, X.; Li, Y.; Wang, J.; Long, J.; Liu, J.; Wang, S.; Li, J.; Gao, F., Long-term simulated microgravity fosters carotid aging-like changes via Piezo1. *Cardiovasc Res* **2024,** 120, (5), 548-559.

199. Singh, R.; Jaiswal, A.; Singh, R. P., Simulated microgravity induces DNA damage concurrent with impairment of DNA repair and activation of cell-type specific DNA damage response in microglial and glioblastoma cells. *Biochim Biophys Acta Mol Cell Res* **2024,** 1871, (3), 119679.

200. Mitrokhin, V. M.; Kamkina, O. V.; Kamkin, A. G.; Rodina, A. S.; Zolotareva, A. D.; Zolotarev, V. I.; Kazansky, V. E.; Gorbacheva, L. R.; Bilichenko, A. S.; Shileiko, S. A.; Mladenov, M. I., Simulated Microgravity and Hypergravity Affect the Expression Level of Soluble Guanylate Cyclase, Adenylate Cyclase, and Phosphodiesterase Genesin Rat Ventricular Cardiomyocytes. *Bull Exp Biol Med* **2024,** 176, (3), 359-362.

201. Bonanni, R.; Cariati, I.; Rinaldi, A. M.; Marini, M.; D'Arcangelo, G.; Tarantino, U.; Tancredi, V., Trolox and recombinant Irisin as a potential strategy to prevent neuronal damage induced by random positioning machine exposure in differentiated HT22 cells. *PLoS One* **2024,** 19, (3), e0300888.

202. Park, J.; Overbey, E. G.; Narayanan, S. A.; Kim, J.; Tierney, B. T.; Damle, N.; Najjar, D.; Ryon, K. A.; Proszynski, J.; Kleinman, A.; Hirschberg, J. W.; MacKay, M.; Afshin, E. E.; Granstein, R.; Gurvitch, J.; Hudson, B. M.; Rininger, A.; Mullane, S.; Church, S. E.; Meydan, C.; Church, G.; Beheshti, A.; Mateus, J.; Mason, C. E., Spatial multi-omics of human skin reveals KRAS and inflammatory responses to spaceflight. *Nat Commun* **2024,** 15, (1), 4773.

203. Ye, R.; He, Y.; Ni, W.; Zhang, Y.; Zhu, Y.; Cao, M.; He, R.; Yao, M., LLLT accelerates experimental wound healing under microgravity conditions via PI3K/AKT-CCR2 signal axis. *Front Bioeng Biotechnol* **2024,** 12, 1387474.

204. Zhai, Y.; Zhang, Y.; Xu, K.; Wang, T.; Zhiqun, B.; Qu, L.; Wu, F.; Hu, Z.; Chang, X.; Li, H.; Zhang, C.; Li, C.; Shi, C., Cordycepin ameliorates spaceflight-induced osteoporosis by preventing BMSCs oxidative stress and senescence via interacting with PI3K p110alpha and regulating PI3K/Akt/FOXO3 signalling. *Free Radic Biol Med* **2025,** 228, 108-125.

205. Hou, H.; Qiu, Z.; Che, J.; Li, Y.; Sun, J.; Zhang, W.; Ma, J.; Zhang, S.; Li, M.; Niu, Y.; He, L., Effects of simulated microgravity on dental pulp stem cell stemness. *J Mol Histol* **2025,** 56, (2), 97.

206. Liu, X.; Zhang, H.; Yan, J.; Ye, P.; Wang, Y.; Zhang, N.; Tian, Z.; Liu, B.; Yang, H., Purine metabolism in bone marrow microenvironment inhibits hematopoietic stem cell differentiation under microgravity. *Stem Cell Res Ther* **2025,** 16, (1), 115.

207. Mu, Y.; Li, W.; Wei, D.; Zhang, X.; Yao, L.; Xu, X.; Wang, X.; Zhang, Z.; Chen, T., The effect of Hydrogen-rich water on retinal degeneration in the outer nuclear layer of simulated weightlessness rats. *Life Sci Space Res (Amst)* **2025,** 45, 158-169.

208. Ranieri, M.; Venneri, M.; Storlino, G.; Ferrulli, A.; D'Agostino, M.; Centrone, M.; Di Mise, A.; Zerlotin, R.; Tamma, G.; Grano, M.; Valenti, G., Alteration of vasopressin-aquaporin system in hindlimb unloading mice. *Front Physiol* **2025,** 16, 1535053.

209. Czeisler, C. A.; Chiasera, A. J.; Duffy, J. F., Research on sleep, circadian rhythms and aging: applications to manned spaceflight. *Exp Gerontol* **1991,** 26, (2-3), 217-32.

210. Schmidt, M. A.; Goodwin, T. J.; Pelligra, R., Incorporation of omics analyses into artificial gravity research for space exploration countermeasure development. *Metabolomics* **2016,** 12, 36.

211. Fujita, S. I.; Rutter, L.; Ong, Q.; Muratani, M., Integrated RNA-seq Analysis Indicates Asynchrony in Clock Genes between Tissues under Spaceflight. *Life (Basel)* **2020,** 10, (9).

212. Urbaniak, C.; Wong, S.; Tighe, S.; Arumugam, A.; Liu, B.; Parker, C. W.; Wood, J. M.; Singh, N. K.; Skorupa, D. J.; Peyton, B. M.; Jenson, R.; Karouia, F.; Dragon, J.; Venkateswaran, K., Validating an Automated Nucleic Acid Extraction Device for Omics in Space Using Whole Cell Microbial Reference Standards. *Front Microbiol* **2020,** 11, 1909.

213. Berrios, D. C.; Galazka, J.; Grigorev, K.; Gebre, S.; Costes, S. V., NASA GeneLab: interfaces for the exploration of space omics data. *Nucleic Acids Res* **2021,** 49, (D1), D1515-D1522.

214. da Silveira, W. A.; Fazelinia, H.; Rosenthal, S. B.; Laiakis, E. C.; Kim, M. S.; Meydan, C.; Kidane, Y.; Rathi, K. S.; Smith, S. M.; Stear, B.; Ying, Y.; Zhang, Y.; Foox, J.; Zanello, S.; Crucian, B.; Wang, D.; Nugent, A.; Costa, H. A.; Zwart, S. R.; Schrepfer, S.; Elworth, R. A. L.; Sapoval, N.; Treangen, T.; MacKay, M.; Gokhale, N. S.; Horner, S. M.; Singh, L. N.; Wallace, D. C.; Willey, J. S.; Schisler, J. C.; Meller, R.; McDonald, J. T.; Fisch, K. M.; Hardiman, G.; Taylor, D.; Mason, C. E.; Costes, S. V.; Beheshti, A., Comprehensive Multi-omics Analysis Reveals Mitochondrial Stress as a Central Biological Hub for Spaceflight Impact. *Cell* **2020,** 183, (5), 1185-1201 e20.

215. Rutter, L.; Barker, R.; Bezdan, D.; Cope, H.; Costes, S. V.; Degoricija, L.; Fisch, K. M.; Gabitto, M. I.; Gebre, S.; Giacomello, S.; Gilroy, S.; Green, S. J.; Mason, C. E.; Reinsch, S. S.; Szewczyk, N. J.; Taylor, D. M.; Galazka, J. M.; Herranz, R.; Muratani, M., A New Era for Space Life Science: International Standards for Space Omics Processing. *Patterns (N Y)* **2020,** 1, (9), 100148.

216. Bezdan, D.; Grigorev, K.; Meydan, C.; Pelissier Vatter, F. A.; Cioffi, M.; Rao, V.; MacKay, M.; Nakahira, K.; Burnham, P.; Afshinnekoo, E.; Westover, C.; Butler, D.; Mozsary, C.; Donahoe, T.; Foox, J.; Mishra, T.; Lucotti, S.; Rana, B. K.; Melnick, A. M.; Zhang, H.; Matei, I.; Kelsen, D.; Yu, K.; Lyden, D. C.; Taylor, L.; Bailey, S. M.; Snyder, M. P.; Garrett-Bakelman, F. E.; Ossowski, S.; De Vlaminck, I.; Mason, C. E., Cell-free DNA (cfDNA) and Exosome Profiling from a Year-Long Human Spaceflight Reveals Circulating Biomarkers. *iScience* **2020,** 23, (12), 101844.

217. Cahill, T.; Cope, H.; Bass, J. J.; Overbey, E. G.; Gilbert, R.; da Silveira, W. A.; Paul, A. M.; Mishra, T.; Herranz, R.; Reinsch, S. S.; Costes, S. V.; Hardiman, G.; Szewczyk, N. J.; Tahimic, C. G. T., Mammalian and Invertebrate Models as Complementary Tools for Gaining Mechanistic Insight on Muscle Responses to Spaceflight. *Int J Mol Sci* **2021,** 22, (17).

218. Schulz, H.; Strauch, S. M.; Richter, P.; Wehland, M.; Kruger, M.; Sahana, J.; Corydon, T. J.; Wise, P.; Baran, R.; Lebert, M.; Grimm, D., Latest knowledge about changes in the proteome in microgravity. *Expert Rev Proteomics* **2022,** 19, (1), 43-59.

219. Pastushkova, L. K.; Goncharov, I. N.; Koloteva, M. I.; Goncharova, A. G.; Kashirina, D. N.; Nosovsky, A. M.; Glebova, T. M.; Kononikhin, A. S.; Borchers, C. H.; Nikolaev, E. N.; Larina, I. M., Characteristics of blood plasma proteome changes associated with the hemorrhagic purpura of cosmonauts on the first day after long-term space missions. *Life Sci Space Res (Amst)* **2022,** 33, 7-12.

220. Overbey, E. G.; Kim, J.; Tierney, B. T.; Park, J.; Houerbi, N.; Lucaci, A. G.; Garcia Medina, S.; Damle, N.; Najjar, D.; Grigorev, K.; Afshin, E. E.; Ryon, K. A.; Sienkiewicz, K.; Patras, L.; Klotz, R.; Ortiz, V.; MacKay, M.; Schweickart, A.; Chin, C. R.; Sierra, M. A.; Valenzuela, M. F.; Dantas, E.; Nelson, T. M.; Cekanaviciute, E.; Deards, G.; Foox, J.; Narayanan, S. A.; Schmidt, C. M.; Schmidt, M. A.; Schmidt, J. C.; Mullane, S.; Tigchelaar, S. S.; Levitte, S.; Westover, C.; Bhattacharya, C.; Lucotti, S.; Wain Hirschberg, J.; Proszynski, J.; Burke, M.; Kleinman, A. S.; Butler, D. J.; Loy, C.; Mzava, O.; Lenz, J.; Paul, D.; Mozsary, C.; Sanders, L. M.; Taylor, L. E.; Patel, C. O.; Khan, S. A.; Suhail Mohamad, M.; Byhaqui, S. G. A.; Aslam, B.; Gajadhar, A. S.; Williamson, L.; Tandel, P.; Yang, Q.; Chu, J.; Benz, R. W.; Siddiqui, A.; Hornburg, D.; Blease, K.; Moreno, J.; Boddicker, A.; Zhao, J.; Lajoie, B.; Scott, R. T.; Gilbert, R. R.; Lai Polo, S. H.; Altomare, A.; Kruglyak, S.; Levy, S.; Ariyapala, I.; Beer, J.; Zhang, B.; Hudson, B. M.; Rininger, A.; Church, S. E.; Beheshti, A.; Church, G. M.; Smith, S. M.; Crucian, B. E.; Zwart, S. R.; Matei, I.; Lyden, D. C.; Garrett-Bakelman, F.; Krumsiek, J.; Chen, Q.; Miller, D.; Shuga, J.; Williams, S.; Nemec, C.; Trudel, G.; Pelchat, M.; Laneuville, O.; De Vlaminck, I.; Gross, S.; Bolton, K. L.; Bailey, S. M.; Granstein, R.; Furman, D.; Melnick, A. M.; Costes, S. V.; Shirah, B.; Yu, M.; Menon, A. S.; Mateus, J.; Meydan, C.; Mason, C. E., The Space Omics and Medical Atlas (SOMA) and international astronaut biobank. *Nature* **2024,** 632, (8027), 1145-1154.

221. Kim, J.; Tierney, B. T.; Overbey, E. G.; Dantas, E.; Fuentealba, M.; Park, J.; Narayanan, S. A.; Wu, F.; Najjar, D.; Chin, C. R.; Meydan, C.; Loy, C.; Mathyk, B.; Klotz, R.; Ortiz, V.; Nguyen, K.; Ryon, K. A.; Damle, N.; Houerbi, N.; Patras, L. I.; Schanzer, N.; Hutchinson, G. A.; Foox, J.; Bhattacharya, C.; Mackay, M.; Afshin, E. E.; Hirschberg, J. W.; Kleinman, A. S.; Schmidt, J. C.; Schmidt, C. M.; Schmidt, M. A.; Beheshti, A.; Matei, I.; Lyden, D.; Mullane, S.; Asadi, A.; Lenz, J. S.; Mzava, O.; Yu, M.; Ganesan, S.; De Vlaminck, I.; Melnick, A. M.; Barisic, D.; Winer, D. A.; Zwart, S. R.; Crucian, B. E.; Smith, S. M.; Mateus, J.; Furman, D.; Mason, C. E., Single-cell multi-ome and immune profiles of the Inspiration4 crew reveal conserved, cell-type, and sex-specific responses to spaceflight. *Nat Commun* **2024,** 15, (1), 4954.

222. Ilangovan, H.; Kothiyal, P.; Hoadley, K. A.; Elgart, R.; Eley, G.; Eslami, P., Harmonizing heterogeneous transcriptomics datasets for machine learning-based analysis to identify spaceflown murine liver-specific changes. *NPJ Microgravity* **2024,** 10, (1), 61.

223. Tierney, B. T.; Kim, J.; Overbey, E. G.; Ryon, K. A.; Foox, J.; Sierra, M. A.; Bhattacharya, C.; Damle, N.; Najjar, D.; Park, J.; Garcia Medina, J. S.; Houerbi, N.; Meydan, C.; Wain Hirschberg, J.; Qiu, J.; Kleinman, A. S.; Al-Ghalith, G. A.; MacKay, M.; Afshin, E. E.; Dhir, R.; Borg, J.; Gatt, C.; Brereton, N.; Readhead, B. P.; Beyaz, S.; Venkateswaran, K. J.; Wiseman, K.; Moreno, J.; Boddicker, A. M.; Zhao, J.; Lajoie, B. R.; Scott, R. T.; Altomare, A.; Kruglyak, S.; Levy, S.; Church, G. M.; Mason, C. E., Longitudinal multi-omics analysis of host microbiome architecture and immune responses during short-term spaceflight. *Nat Microbiol* **2024,** 9, (7), 1661-1675.

224. Abdelfattah, F.; Schulz, H.; Wehland, M.; Corydon, T. J.; Sahana, J.; Kraus, A.; Kruger, M.; Gonzalez-Torres, L. F.; Cortes-Sanchez, J. L.; Wise, P. M.; Mushunuri, A.; Hemmersbach, R.; Liemersdorf, C.; Infanger, M.; Grimm, D., Omics Studies of Specialized Cells and Stem Cells under Microgravity Conditions. *Int J Mol Sci* **2024,** 25, (18).

225. Schulz, H.; Abdelfattah, F.; Heinrich, A.; Melnik, D.; Sandt, V.; Kruger, M.; Wehland, M.; Hoffmann, P.; Cortes-Sanchez, J. L.; Evert, M.; Evert, K.; Grimm, D., Omics Investigations of Prostate Cancer Cells Exposed to Simulated Microgravity Conditions. *Biomolecules* **2025,** 15, (2).

226. Wang, S.; Zhang, N.; Shi, G.; Liu, X.; Zhou, Y.; Yang, H., Genome-wide chromatin accessibility and transcriptome analysis reveal the up-regulation of immunosuppressive genes in macrophages under simulated microgravity. *Life Sci Space Res (Amst)* **2025,** 45, 143-150.

227. Unsworth, B. R.; Lelkes, P. I., Growing tissues in microgravity. *Nat Med* **1998,** 4, (8), 901-7.

228. Navran, S., The application of low shear modeled microgravity to 3-D cell biology and tissue engineering. *Biotechnol Annu Rev* **2008,** 14, 275-96.

229. Becker, J. L.; Souza, G. R., Using space-based investigations to inform cancer research on Earth. *Nat Rev Cancer* **2013,** 13, (5), 315-27.

230. Ulbrich, C.; Wehland, M.; Pietsch, J.; Aleshcheva, G.; Wise, P.; van Loon, J.; Magnusson, N.; Infanger, M.; Grosse, J.; Eilles, C.; Sundaresan, A.; Grimm, D., The impact of simulated and real microgravity on bone cells and mesenchymal stem cells. *Biomed Res Int* **2014,** 2014, 928507.

231. Wuest, S. L.; Richard, S.; Kopp, S.; Grimm, D.; Egli, M., Simulated microgravity: critical review on the use of random positioning machines for mammalian cell culture. *Biomed Res Int* **2015,** 2015, 971474.

232. Koch, C.; Kohn, F. P.; Bauer, J., Preparing normal tissue cells for space flight experiments. *Prep Biochem Biotechnol* **2016,** 46, (2), 208-13.

233. Aleshcheva, G.; Bauer, J.; Hemmersbach, R.; Slumstrup, L.; Wehland, M.; Infanger, M.; Grimm, D., Scaffold-free Tissue Formation Under Real and Simulated Microgravity Conditions. *Basic Clin Pharmacol Toxicol* **2016,** 119 Suppl 3, 26-33.

234. Hammond, T.; Allen, P.; Birdsall, H., Is There a Space-Based Technology Solution to Problems with Preclinical Drug Toxicity Testing? *Pharm Res* **2016,** 33, (7), 1545-51.

235. Chen, Z. Y.; Guo, S.; Li, B. B.; Jiang, N.; Li, A.; Yan, H. F.; Yang, H. M.; Zhou, J. L.; Li, C. L.; Cui, Y., Effect of Weightlessness on the 3D Structure Formation and Physiologic Function of Human Cancer Cells. *Biomed Res Int* **2019,** 2019, 4894083.

236. Swaminathan, V.; Bechtel, G.; Tchantchaleishvili, V., Artificial tissue creation under microgravity conditions: Considerations and future applications. *Artif Organs* **2021,** 45, (12), 1446-1455.

237. Ma, C.; Duan, X.; Lei, X., 3D cell culture model: From ground experiment to microgravity study. *Front Bioeng Biotechnol* **2023,** 11, 1136583.

238. Nie, H. Y.; Ge, J.; Liu, K. G.; Yue, Y.; Li, H.; Lin, H. G.; Yan, H. F.; Zhang, T.; Sun, H. W.; Yang, J. W.; Zhou, J. L.; Cui, Y., The effects of microgravity on stem cells and the new insights it brings to tissue engineering and regenerative medicine. *Life Sci Space Res (Amst)* **2024,** 41, 1-17.

239. Jogdand, A.; Landolina, M.; Chen, Y., Organs in orbit: how tissue chip technology benefits from microgravity, a perspective. *Front Lab Chip Technol* **2024,** 3.

240. Kitajima, Y.; Aoyama, Y.; Seishima, M., Transmembrane signaling for adhesive regulation of desmosomes and hemidesmosomes, and for cell-cell datachment induced by pemphigus IgG in cultured keratinocytes: involvement of protein kinase C. *J Investig Dermatol Symp Proc* **1999,** 4, (2), 137-44.

241. Jongkind, J. F.; Visser, P.; Verkerk, A., Cell fusion in space: plasma membrane fusion in human fibroblasts during short term microgravity. *Adv Space Res* **1996,** 17, (6-7), 21-5.

242. Park, H. Y.; Gilchrest, B. A., Protein kinase C: biochemical characteristics and role in melanocyte biology. *J Dermatol Sci* **1993,** 6, (3), 185-93.

243. Dai, Z. Q.; Wang, R.; Ling, S. K.; Wan, Y. M.; Li, Y. H., Simulated microgravity inhibits the proliferation and osteogenesis of rat bone marrow mesenchymal stem cells. *Cell Prolif* **2007,** 40, (5), 671-84.

244. Huang, W.; Chen, C.; Liu, X., Hindlimb suspension-induced cell apoptosis in the posterior parietal cortex and lateral geniculate nucleus: corresponding changes in c-Fos protein and the PI3K/Akt signaling pathway. *Acta Neurobiol Exp (Wars)* **2018,** 78, (3), 220-230.

245. Hybel, T. E.; Dietrichs, D.; Sahana, J.; Corydon, T. J.; Nassef, M. Z.; Wehland, M.; Kruger, M.; Magnusson, N. E.; Bauer, J.; Utpatel, K.; Infanger, M.; Grimm, D.; Kopp, S., Simulated Microgravity Influences VEGF, MAPK, and PAM Signaling in Prostate Cancer Cells. *Int J Mol Sci* **2020,** 21, (4).

246. Launius, R. D., *Frontiers of space exploration*. Greenwood Press: Westport, Conn., 1998; p xxxi, 204 p.

247. Mednieks, M. I.; Fine, A. S.; Oyama, J.; Philpott, D. E., Cardiac muscle ultrastructure and cyclic AMP reactions to altered gravity conditions. *Am J Physiol* **1987,** 252, (2 Pt 2), R227-32.

248. Buravkova, L. B.; Romanov, Y. A., The role of cytoskeleton in cell changes under condition of simulated microgravity. *Acta Astronaut* **2001,** 48, (5-12), 647-50.

249. Merzlikina, N. V.; Buravkova, L. B.; Romanov, Y. A., The primary effects of clinorotation on cultured human mesenchymal stem cells. *J Gravit Physiol* **2004,** 11, (2), P193-4.

250. Ogneva, I. V.; Mirzoev, T. M.; Biryukov, N. S.; Veselova, O. M.; Larina, I. M., Structure and functional characteristics of rat's left ventricle cardiomyocytes under antiorthostatic suspension of various duration and subsequent reloading. *J Biomed Biotechnol* **2012,** 2012, 659869.

251. Ogneva, I. V.; Maximova, M. V.; Larina, I. M., Structure of cortical cytoskeleton in fibers of mouse muscle cells after being exposed to a 30-day space flight on board the BION-M1 biosatellite. *J Appl Physiol (1985)* **2014,** 116, (10), 1315-23.

252. Ogneva, I. V.; Gnyubkin, V.; Laroche, N.; Maximova, M. V.; Larina, I. M.; Vico, L., Structure of the cortical cytoskeleton in fibers of postural muscles and cardiomyocytes of mice after 30-day 2-g centrifugation. *J Appl Physiol (1985)* **2015,** 118, (5), 613-23.

253. Ulanova, A.; Gritsyna, Y.; Vikhlyantsev, I.; Salmov, N.; Bobylev, A.; Abdusalamova, Z.; Rogachevsky, V.; Shenkman, B.; Podlubnaya, Z., Isoform composition and gene expression of thick and thin filament proteins in striated muscles of mice after 30-day space flight. *Biomed Res Int* **2015,** 2015, 104735.

254. Rudimov, E. G.; Buravkov, S. V.; Andreeva, E. P.; Buravkova, L. B., Effect of proinflammatory activation on F-actin distribution in cultured human endothelial cells under conditions of experimental microgravity. *Bull Exp Biol Med* **2015,** 158, (4), 573-80.

255. Ogneva, I. V.; Maximova, M. V.; Larina, I. M., [Desmin content and transversal stiffness of the left ventricle mouse cardiomyocytes and skeletal muscle fibers after a 30-day space flight on board "BION-M1" biosatellite]. *Biofizika* **2014,** 59, (5), 983-9.

256. Ogneva, I. V.; Biryukov, N. S., Lecithin Prevents Cortical Cytoskeleton Reorganization in Rat Soleus Muscle Fibers under Short-Term Gravitational Disuse. *PLoS One* **2016,** 11, (4), e0153650.

257. Porseva, V. V.; Shilkin, V. V.; Strelkov, A. A.; Krasnov, I. B.; Masliukov, P. M., Changes in the Neurochemical Composition of Motor Neurons of the Spinal Cord in Mice under Conditions of Space Flight. *Bull Exp Biol Med* **2017,** 162, (3), 336-339.

258. Ogneva, I. V.; Loktev, S. S.; Sychev, V. N., Cytoskeleton structure and total methylation of mouse cardiac and lung tissue during space flight. *PLoS One* **2018,** 13, (5), e0192643.

259. Loktev, S. S.; Ogneva, I. V., DNA Methylation of Mouse Testes, Cardiac and Lung Tissue During Long-Term Microgravity Simulation. *Sci Rep* **2019,** 9, (1), 7974.

260. Ogneva, I. V.; Usik, M. A.; Loktev, S. S.; Zhdankina, Y. S.; Biryukov, N. S.; Orlov, O. I.; Sychev, V. N., Testes and duct deferens of mice during space flight: cytoskeleton structure, sperm-specific proteins and epigenetic events. *Sci Rep* **2019,** 9, (1), 9730.

261. Kononikhin, A. S.; Brzhozovskiy, A. G.; Ryabokon, A. M.; Fedorchenko, K.; Zakharova, N. V.; Spasskii, A. I.; Popov, I. A.; Ilyin, V. K.; Solovyova, Z. O.; Pastushkova, L. K.; Polyakov, A. V.; Varfolomeev, S. D.; Larina, I. M.; Nikolaev, E. N., Proteome Profiling of the Exhaled Breath Condensate after Long-Term Spaceflights. *Int J Mol Sci* **2019,** 20, (18).

262. Ogneva, I. V.; Usik, M. A., Mitochondrial Respiration in Drosophila Ovaries after a Full Cycle of Oogenesis under Simulated Microgravity. *Curr Issues Mol Biol* **2021,** 43, (1), 176-186.

263. Shenkman, B. S.; Tsaturyan, A. K.; Vikhlyantsev, I. M.; Kozlovskaya, I. B.; Grigoriev, A. I., Molecular Mechanisms of Muscle Tone Impairment under Conditions of Real and Simulated Space Flight. *Acta Naturae* **2021,** 13, (2), 85-97.

264. Shishkina, V.; Kostin, A.; Volodkin, A.; Samoilova, V.; Buchwalow, I.; Tiemann, M.; Atiakshin, D., The Remodeling of Dermal Collagen Fibrous Structures in Mice under Zero Gravity: The Role of Mast Cells. *Int J Mol Sci* **2023,** 24, (3).

265. Samoilenko, T.; Shishkina, V.; Antakova, L.; Goryushkina, Y.; Kostin, A.; Buchwalow, I.; Tiemann, M.; Atiakshin, D., Smooth Muscle Actin as a Criterion for Gravisensitivity of Stomach and Jejunum in Laboratory Rodents. *Int J Mol Sci* **2023,** 24, (22).

266. Gorbacheva, E. Y.; Sventitskaya, M. A.; Biryukov, N. S.; Ogneva, I. V., The Oxidative Phosphorylation and Cytoskeleton Proteins of Mouse Ovaries after 96 Hours of Hindlimb Suspension. *Life (Basel)* **2023,** 13, (12).

267. Sventitskaya, M. A.; Ogneva, I. V., Reorganization of the mouse oocyte' cytoskeleton after cultivation under simulated weightlessness. *Life Sci Space Res (Amst)* **2024,** 40, 8-18.

268. Kashirina, D. N.; Pastushkova, L. K.; Kononikhin, A. S.; Brzhozovskiy, A. G.; Larina, I. M., Influence of a Passive Tilt Test on the Proteomic Composition of the Blood of Healthy Humans. *Bull Exp Biol Med* **2024,** 176, (3), 394-398.

269. Mao, X. W.; Sandberg, L. B.; Gridley, D. S.; Herrmann, E. C.; Zhang, G.; Raghavan, R.; Zubarev, R. A.; Zhang, B.; Stodieck, L. S.; Ferguson, V. L.; Bateman, T. A.; Pecaut, M. J., Proteomic Analysis of Mouse Brain Subjected to Spaceflight. *Int J Mol Sci* **2018,** 20, (1).

270. Kashirina, D. N.; Pastushkova, L. K.; Goncharova, A. G.; Larina, I. M., Assessment of the effect of 21-day head-down bed rest on the cardiovascular system by blood protein composition. *Front Physiol* **2024,** 15, 1375929.

271. Tyrina, E.; Yakubets, D.; Markina, E.; Buravkova, L., Hippo Signaling Pathway Involvement in Osteopotential Regulation of Murine Bone Marrow Cells Under Simulated Microgravity. *Cells* **2024,** 13, (22).

272. Novoselova, E. G.; Lunin, S. M.; Khrenov, M. O.; Parfenyuk, S. B.; Novoselova, T. V.; Shenkman, B. S.; Fesenko, E. E., Changes in immune cell signalling, apoptosis and stress response functions in mice returned from the BION-M1 mission in space. *Immunobiology* **2015,** 220, (4), 500-9.

273. Rudimov, E. G.; Knjazev, E. N.; Khaustova, N. A.; Grigorieva, O. V.; Buravkova, L. B., Transcriptomic changes in human umbilical cord blood endothelial cells under simulated microgravity. *Dokl Biochem Biophys* **2017,** 472, (1), 1-4.

274. Pastushkova, L. H.; Rusanov, V. B.; Goncharova, A. G.; Brzhozovskiy, A. G.; Kononikhin, A. S.; Chernikova, A. G.; Kashirina, D. N.; Nosovsky, A. M.; Baevsky, R. M.; Nikolaev, E. N.; Larina, I. M., Urine proteome changes associated with autonomic regulation of heart rate in cosmonauts. *BMC Syst Biol* **2019,** 13, (Suppl 1), 17.

275. Ratushnyy, A. Y.; Tyrina, E. A.; Buravkova, L. B., Simulation of Microgravity and Coculturing with Hematopoietic Cells Oppositely Modulate Wnt Signaling in Mesenchymal Stromal Cells. *Dokl Biochem Biophys* **2023,** 510, (1), 95-98.

276. Sergeeva, X. V.; Sharlo, K. A.; Tyganov, S. A.; Kalashnikov, V. E.; Shenkman, B. S., Molecular Signaling Effects behind the Spontaneous Soleus Muscle Activity Induced by 7-Day Rat Hindlimb Suspension. *Int J Mol Sci* **2024,** 25, (15).

277. Kuznetsov, N. V.; Vlasova, D. D.; Kotikova, A. A.; Tomilovskaya, E.; Ljubisavljevic, M., Microgravity Impacts the Expression of Aging-Associated Candidate Gene Targets in the p53 Regulatory Network. *Int J Mol Sci* **2025,** 26, (22).

Disclaimer/Publisher's Note: The statements, opinions and data contained in all publications are solely those of the individual author(s) and contributor(s) and not of MDPI and/or the editor(s). MDPI and/or the editor(s) disclaim responsibility for any injury to people or property resulting from any ideas, methods, instructions or products referred to in the content.
